# Effectiveness and cost-effectiveness of a web-based cardiac rehabilitation programme for people with chronic stable angina: protocol for the ACTIVATE (Angina Controlled Trial Investigating the Value of the ‘Activate your heart’ Therapeutic E-intervention) randomised controlled trial

**DOI:** 10.1136/bmjopen-2024-084509

**Published:** 2024-03-25

**Authors:** Nefyn H Williams, Brendan Collins, Terence J Comerford, Susanna Dodd, Michael Fisher, Ben Hardwick, Sophie Hennessy, Kate Jolly, Ian Jones, Deirdre Lane, Gregory Y H Lip, Erica Morgan, Penelope Ralph, Dick Thijssen, Sally J Singh

**Affiliations:** 1 Department of Primary Care and Mental Health, University of Liverpool, Liverpool, UK; 2 Department of Public Health Policy and Systems, University of Liverpool, Liverpool, UK; 3 Patient and Public Involvement, National Institute for Health and Care Research Applied Research Collaboration North West Coast, Liverpool, UK; 4 Department of Health Data Science, University of Liverpool, Liverpool, UK; 5 Liverpool University Hospitals NHS Foundation Trust, Liverpool, UK; 6 Liverpool Clinical Trials Centre, University of Liverpool, Liverpool, UK; 7 Institute of Applied Health Research, University of Birmingham, Birmingham, UK; 8 School of Nursing and Allied Health, Liverpool John Moores University, Liverpool, UK; 9 Department of Cardiovascular and Metabolic Medicine, University of Liverpool, Liverpool, UK; 10 Liverpool Centre for Cardiovascular Science, University of Liverpool, Liverpool John Moores University and Liverpool Heart and Chest Hospital, Liverpool, UK; 11 School of Sport and Exercise Sciences, Liverpool John Moores University, Liverpool, UK; 12 Department of Respiratory Sciences, University of Leicester, Leicester, UK

**Keywords:** Ischaemic heart disease, Primary Care, REHABILITATION MEDICINE, Randomized Controlled Trial, Adult cardiology, HEALTH ECONOMICS

## Abstract

**Introduction:**

Chronic stable angina is common and disabling. Cardiac rehabilitation is routinely offered to people following myocardial infarction or revascularisation procedures and has the potential to help people with chronic stable angina. However, there is insufficient evidence of effectiveness and cost-effectiveness for its routine use in this patient group. The objectives of this study are to compare the effectiveness and cost-effectiveness of the ‘Activate Your Heart’ cardiac rehabilitation programme for people with chronic stable angina compared with usual care.

**Methods and analysis:**

ACTIVATE is a multicentre, parallel-group, two-arm, superiority, pragmatic randomised controlled trial, with recruitment from primary and secondary care centres in England and Wales and a target sample size of 518 (1:1 allocation; allocation sequence by minimisation programme with built-in random element). The study uses secure web-based allocation concealment. The two treatments will be optimal usual care (control) and optimal usual care plus the ‘Activate Your Heart’ web-based cardiac rehabilitation programme (intervention). Outcome assessment and statistical analysis will be performed blinded; participants will be unblinded. Outcomes will be measured at baseline and at 6 and 12 months’ follow-up. Primary outcome will be the UK version of Seattle Angina Questionnaire (SAQ-UK), physical limitations domain at 12 months’ follow-up. Secondary outcomes will be the remaining two domains of SAQ-UK, dyspnoea, anxiety and depression, health utility, self-efficacy, physical activity and the incremental shuttle walk test. All safety events will be recorded, and serious adverse events assessed to determine whether they are related to the intervention and expected. Concurrent economic evaluation will be cost–utility analysis from health service perspective. An embedded process evaluation will determine the mechanisms and processes that explain the implementation and impacts of the cardiac rehabilitation programme.

**Ethics and dissemination:**

North of Scotland National Health Service Research Ethics Committee approval, reference 21/NS/0115. Participants will provide written informed consent. Results will be disseminated by peer-reviewed publication.

**Trial registration number:**

ISRCTN10054455.

**STRENGTHS AND LIMITATIONS OF THIS STUDY**

- Pragmatic randomised controlled trial of an established cardiac rehabilitation programme for individuals with chronic stable angina, with unblinded participants and rehabilitation providers.
- Concurrent economic evaluation with a health service perspective.
- Embedded process evaluation to determine the mechanisms and processes that explain the implementation and impacts of the enhanced rehabilitation programme.
- The web-based intervention may not be acceptable to some people, but a paper version is available.

## Introduction

Angina is chest pain usually caused by atherosclerosis of the coronary arteries, which restricts blood flow to the myocardium, especially when oxygen demand is increased during exercise. Angina is defined as stable if chest pain occurs predictably on physical exertion, or with emotion, and settles promptly with rest or after taking sublingual nitrate medication. Affected individuals have an increased risk of acute coronary events such as myocardial infarction (MI).^1^ Management consists of behaviour change advice to reduce risk factor profile, drug treatment and revascularisation procedures.^2 3^

Cardiac rehabilitation is routinely offered to patients following MI, or revascularisation procedures, and consists of co-ordinated activities to improve the underlying cause and restore optimal functioning and slow, or reverse, disease progression.^4^ The most prevalent components of cardiac rehabilitation are behaviour change and exercise training, but comprehensive cardiac rehabilitation programmes also include education, interventions to improve mental health, involvement of friends and family members and the development of long-term strategies beyond participation in the cardiac rehabilitation programme. Despite international guidance,^2 5^ such programmes are not offered routinely to people with chronic stable angina.^6^ In the UK, the National Institute for Health and Care Excellence (NICE) does not support cardiac rehabilitation in this group until stronger evidence of effectiveness and cost-effectiveness is available.^3^ Although there is a good evidence for the effectiveness of cardiac rehabilitation for heart failure,^7^ or following MI or revascularisation procedures,^8^ rates of uptake and long-term adherence are low, especially in women, ethnic minority groups, and in areas of high deprivation.^6^

A systematic review of exercise-based cardiac rehabilitation for adults with stable angina,^9^ identified seven randomised controlled trials (RCTs) (n=581) with high risk of bias. It found low-quality evidence that cardiac rehabilitation resulted in a small improvement in exercise capacity compared with usual care with standard mean difference 0.45 (95% CI 0.2 to 0.7), and uncertainty about the effect on all-cause mortality, acute MI, cardiovascular-related hospital admissions, quality of life and cardiac rehabilitation-related adverse events. These RCTs were small in size and recruited mainly white middle-aged men. More evidence is needed from under-represented groups including women, older age groups, ethnic minorities and those with comorbid conditions. Web-based programmes might increase the reach of cardiac rehabilitation, and there has been one small RCT (n=94) of such a programme (‘Activate Your Heart’), which demonstrated that it was possible to provide cardiac rehabilitation to people with chronic stable angina.^10^ Trial methods were feasible in terms of recruitment, retention and outcome measurement. After 6 months, there were statistically significant lower levels of angina frequency, but a larger RCT is needed to test longer-term effectiveness and cost-effectiveness.

‘Activate Your Heart’ has the potential to improve physical and mental health with minimal risk. There may be a risk of injury or provoking an episode of angina when exercising, however, the physical activity goals are carefully graded and the programme has an excellent safety record.^10^

### Aims and objectives

We aim to assess whether a web-based cardiac rehabilitation programme can improve the health of people with chronic stable angina, whether it is good value for money and delivered as intended.

The primary objective is to determine the effectiveness of the ‘Activate Your Heart’ web-based cardiac rehabilitation programme (or a paper version for those who prefer or who do not have internet access or device), for people with chronic stable angina compared with usual care in terms of angina-related health status, at 12 months’ follow-up.

Secondary objectives:

- To estimate the cost-effectiveness of the cardiac rehabilitation programme for people with chronic stable angina compared with usual care in a cost–utility analysis with a health service perspective.
- To investigate the mechanisms and processes that explain the implementation and impacts of the cardiac rehabilitation programme in the intervention group and usual care in the control group.
- To identify the barriers and facilitators of uptake and completion of the rehabilitation programme with a focus on equity.

## Methods and analysis

### Trial design

**Figure 1:**
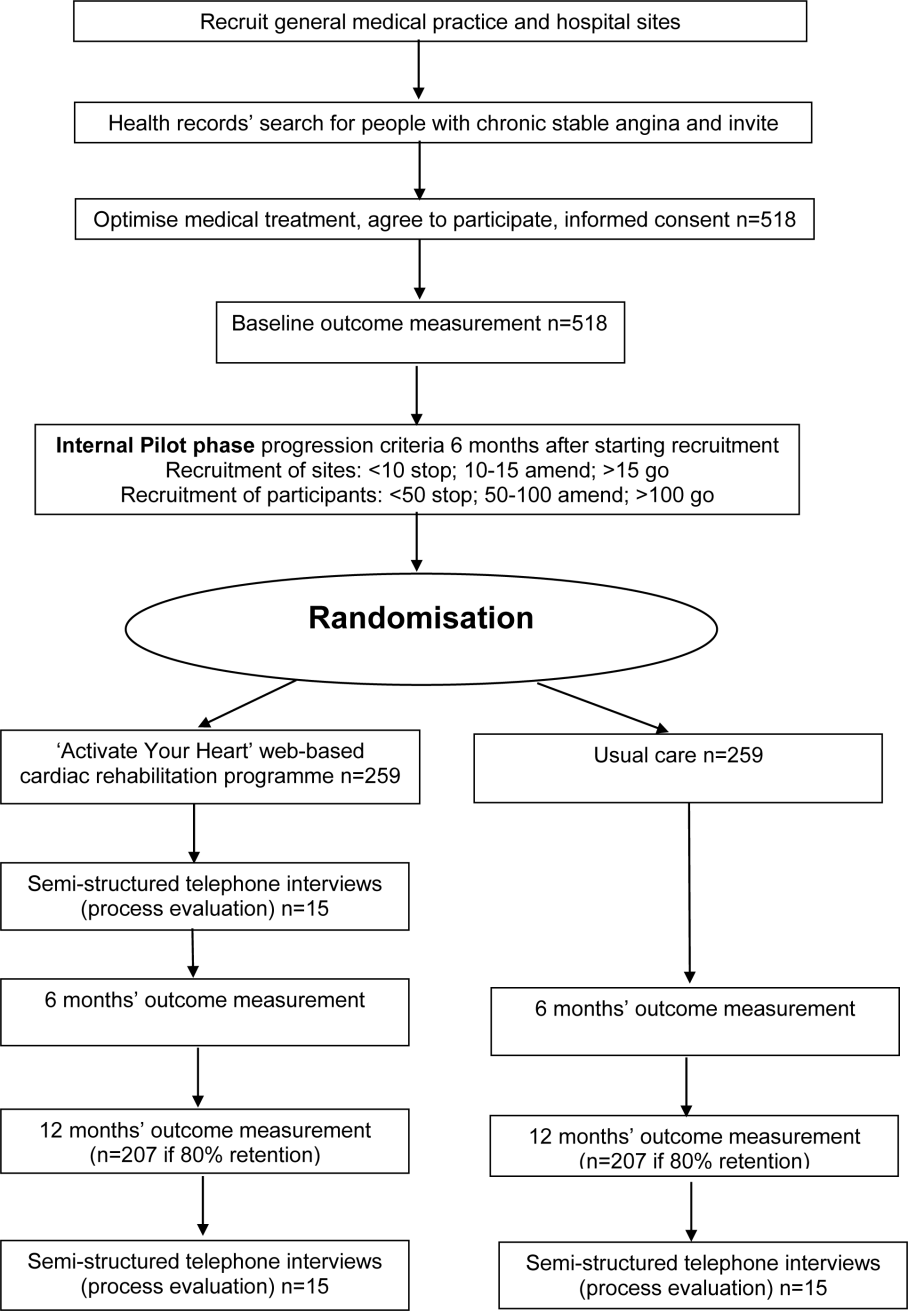
ACTIVATE participant flow chart.

ACTIVATE is a multisite, parallel-group, two-armed, superiority RCT with 1:1 allocation, stratified by gender and recruitment site (figure 1). A pragmatic RCT of effectiveness compared with usual care, with blinded outcome assessment and statistical analysis; unblinded participants and rehabilitation providers. An internal pilot phase will assess feasibility. There will be a concurrent economic evaluation with a health service perspective. An embedded process evaluation will use mixed methods to examine the mechanisms and processes that explain the implementation and impacts of the enhanced rehabilitation programme, including an examination of barriers leading to health inequities. The Health Inequalities Assessment Toolkit (www.hiat.org.uk) informed trial design. The RCT was registered on 16 September 2021 (ISRCTN10054455). Trial registration data can be found in online supplemental appendix 1.

10.1136/bmjopen-2024-084509.supp1 Supplementary data

### Trial setting and selection of sites/clinicians

Participants will be recruited from primary and secondary care in areas of England and Wales with high disease burden and socioeconomic deprivation. Many of these areas include high proportions of ethnic minority groups. The web-based rehabilitation intervention (or paper version) will be delivered in the community.

### Trial population

#### Inclusion criteria

- Adults with chronic stable angina with at least two out of three of the following features: constricting central chest pain, precipitated by exertion or emotional stress, relieved by rest or glyceryl trinitrate spray.
- Evidence of myocardial ischaemia from either a medical history of acute coronary syndrome, MI or revascularisation procedure at least 12 months previously; or from imaging studies such as invasive coronary angiography, CT angiography or myocardial perfusion testing.
- Revascularisation procedures not planned and managed with medical treatments only, including people with previous MI, or previous revascularisation procedure who may have attended cardiac rehabilitation in the past.

#### Exclusion criteria

- History of MI, revascularisation or cardiac rehabilitation participation within the last 12 months.
- Significant comorbidities (as deemed by the person confirming eligibility) that would limit participation in the exercise-based rehabilitation programme.
- Refractory angina on maximal medical therapy.

### Trial treatment/interventions

We plan to compare web-based cardiac rehabilitation in addition to optimal usual care (intervention) with optimal usual care alone.

#### Optimal usual care

Usual care will be optimised in all participants. All participants will be seen by their general practitioner or cardiology team, using a protocol informed by NICE guidance^3^ to ensure that all are receiving optimal care. Considering the pragmatic nature of the proposed RCT, no restrictions will be placed on usual care. NICE guidance^3^ recommends that patients with chronic stable angina receive adequate information and support, lifestyle advice about smoking, exercise, diet and weight control. Episodes of angina should be treated with short-acting nitrate medication. Antianginal drug treatment to prevent episodes of angina consists of a beta-blocker or calcium channel blocker in the first instance; second-line drugs (such as ivabradine, ranolazine) will be used as needed. In addition, drugs for secondary prevention of cardiovascular disease such as MI or stroke, include aspirin, ACE inhibitor (especially if diabetes is present), statins in line with NICE guidance (and treatment targets) on lipid modification^11^ and antihypertensives in line with NICE guidance (and treatment targets) on hypertension.^12^

Concomitant medication will be recorded as part of the health economic data collection. There are no restrictions on any medications. Participants will not have participated in a cardiac rehabilitation programme over the previous 12 months, but other schemes such as smoking cessation, exercise referral and weight management schemes may be available and accessible to participants. Where participants have engaged in one or more of these it will be recorded.

#### Cardiac rehabilitation intervention

Usual care will be optimised as described above. In addition, participants in the intervention arm will be given access to ‘Activate Your Heart’ (www.activateyourheart.org.uk), which is an online interactive, secure and password-protected website designed for participants to use at home. The programme was coproduced with healthcare professionals, patients/members of the public and software designers. The programme aims to deliver the five core components described by the British Association for Cardiovascular Prevention and Rehabilitation: health behaviour change and education, lifestyle risk factor management, medical risk factor management, psychosocial health and long-term management.^4^ The programme uses the following behaviour change techniques: goal setting, self-monitoring, feedback on behaviour, graded tasks, social reward, providing information about health consequences and reducing negative emotions. The programme is tailored to individual need, organised in four stages, which can be completed in 8 weeks, but access to the site and its features continues for 12 months. Before beginning the programme, each participant sees a member of cardiac rehabilitation staff who provides training on the website either face-to-face, or remotely by telephone or video consultation. The participant will complete an online registration form, which records their medical history and cardiac risk factors. A paper-based manual is available for participants who are unable or unwilling to use the online rehabilitation system.^13^ The website generates a tailored plan from this information, consisting of individualised goals for exercise, diet (eating more fruit and vegetables and reducing salt), emotions (managing stress, anxiety and low mood) and smoking. Adherence to these goals is regularly assessed by a short set of questions and feedback on performance. As the participant progresses through the programme, goals become increasingly difficult. Each participant records details of their daily exercise in an online diary, and regular feedback is given. Smokers are provided with feedback about the amount of money they are saving. The programme also contains written information about the health consequences of heart disease and risk factors (exercise, diet, sexual activity, driving, returning to work, hobbies, holidays, benefits, smoking, anxiety and mood). In addition, the programme reduces negative emotions by providing advice about stress reduction and anxiety management skills. There is access to an online discussion forum, and ‘ask the expert’ email facility. Cardiac rehabilitation staff can monitor participants’ progress and respond to questions posted via email. After 12 weeks, the cardiac rehabilitation staff member will see the participant again to discuss progress and local exercise opportunities. Cardiac rehabilitation staff will report any safety events that they become aware of.

### Outcomes

Participants will complete outcome measures at baseline, 6 and 12 months, administered by practice staff blinded to participant allocation, which are from the standardised outcome measurement for patients with coronary artery disease, agreed by the International Consortium for Health Outcomes Measurement^14^ (tables 1 and 2). The primary outcome will be the UK Version of Seattle Angina Questionnaire (SAQ-UK) Physical Limitation domain^15^ assessed at 12 months’ follow-up. Participants completed secondary outcomes will include the remaining two domains of the SAQ-UK,^15^ Medical Research Council (MRC) dyspnoea scale,^16^ Hospital Anxiety and Depression Scale^17^ and General Self-Efficacy Scale.^18^ Physical activity will be measured where possible with the ActivPAL accelerometer^19^ for 7 days, supplemented by the International Physical Activity Questionnaire.^20^ Cardiovascular fitness will be measured with the Incremental Shuttle Walk Test.^21^ Economic measures will be EuroQol-5 Dimensions-5 Levels (EQ-5D-5L)^22^ and Client Service Receipt Inventory (CSRI)^23^ as well as intervention costs. Baseline assessments will also include height, weight, blood pressure and serum cholesterol within the last 12 months.

**Table 1:** ACTIVATE outcome measures

Patient completed measure—primary	Description	Range
UK version of Seattle Angina Questionnaire (SAQ-UK) Physical Limitation domain^15^	Measures how daily activities are limited by angina over the past 4 weeks. Items are weighted equally, summed and transformed to a 0–100 score. Higher score indicates better functioning.	(0–100)
Patient completed measures—secondary		
SAQ-UK Angina Frequency and Perception and Treatment Satisfaction domains^15^	Remaining two domains of SAQ-UK. Items are weighted equally, summed and transformed to a 0–100 score. Higher scores represent higher levels of health or satisfaction with treatment.	(0–100)
MRC dyspnoea scale^16^	Five item questionnaire that assesses the degree of shortness of breath with common activities. One point is assigned to each activity associated with dyspnoea. Higher scores indicates more limitation due to dyspnoea.	(0–5)
Hospital Anxiety and Depression Scale^17^	Measures anxiety (seven items) and depression (seven items) in patients with physical health problems. Two subscales with higher scores indicating greater anxiety or depression.	(0–21)
General Self-Efficacy scale^18^	Measures optimistic self-belief to cope with a variety of difficult demands in life. Higher score indicates greater self-efficacy.	(10–40)
International Physical Activity Questionnaire^20^	This questionnaire supplements the accelerometer data by providing self-reported duration (minutes) and frequency (days) of physical activity in the previous 7 days in the domains: job related, transportation, housework, recreation and sitting.	Minutes of light, moderate and vigorous physical activity
Patient Completed Economic Measures		
EQ-5D-5L^22^	Health utility index with five dimensions (mobility, self-care, usual activities, pain/discomfort, anxiety/depression) and five levels to give health states converted to a utility weight. Also Visual Analogue Score (VAS) for health state today	Health utility weight from 0 (death) to 1.0 (perfect health) also with negative values VAS (0–100)
Client Service Receipt Inventory^23^	Collects information about health service use, including medication, smoking cessation, diet management, weight control programmes and exercise referral schemes.	According to activity
Objective measures of physical activity and function		
Physical activity measured with the ActivPAL accelerometer^19^	The ActivPAL device is a small, slim monitor worn on the thigh. It provides accelerometer-derived information about thigh position and acceleration to determine body posture (sitting, lying or upright), stepping and stepping speed (cadence), from which energy expenditure can be inferred. It will be worn for 7 days to measure step count, energy expenditure, duration of sedentary, light, moderate and vigorous physical activity. Participants will be asked to keep a wear diary documenting working hours, sleep, removal reasons and any other comments.	Step count, energy expenditure, duration of sedentary, light, moderate and vigorous physical activity
Incremental Shuttle Walk Test^21^	Progressive walking test that requires the participant to walk up and down a 10 m course, at a speed dictated by an audio signal. The time between signals is reduced every minute and there are 12 levels. The test ends when the participant either (a) is too breathless to maintain the required speed or (b) fails to complete a shuttle in the time allowed or (c) reaches 85% of the predicted maximal heart rate.	Metres walked

EQ-5D-5L, EuroQol-5 Dimensions-5 Levels; MRC, Medical Research Council.

**Table 2:** ACTIVATE protocol schedule of forms and procedures

Procedures	Screening	Baseline/randomisation	7 days postrandomisation*	Trial intervention†	26 weeks postrandomisation	27 weeks postrandomisation*	52 weeks postrandomisation	53 weeks postrandomisation*
Eligibility screening and consent								
Assessment of eligibility	X							
Informed consent	X							
Confirm consent		X		X	X		X	
Randomisation		X						
Vital signs, height, weight, serum cholesterol		X						
Outcome measurement								
Seattle Angina Questionnaire		X			X		X	
MRC dyspnoea scale		X			X		X	
Hospital Anxiety and Depression Scale		X			X		X	
General Self-Efficacy scale		X			X		X	
EQ-5D-5L		X			X		X	
Client Service Receipt Inventory		X			X		X	
Physical activity measured with ActivPal accelerometer		X	X*		X	X*	X	X*
International Physical Activity Questionnaire			X			X		X
Incremental shuttle walk test		X			X		X	
Safety event reporting								
Monitoring of adverse events			X	X	X	X	X	X
Monitoring of serious adverse events			X	X	X	X	X	X

*Removal of ActivPal after 7 days.

†If randomised to intervention arm, participants will be invited to meet with member of cardiac rehabilitation staff who will train the participant on how to use the intervention.

EQ-5D-5L, EuroQol-5 Dimensions-5 Levels.

Qualitative interviews will take place with a selection of participants at the end of the 12-week cardiac rehabilitation programme among those receiving the intervention, and between months 11 and 12 with the same sample of participants in the intervention group and an additional sample from the control group. These will gather data on trial participation and intervention design (see the ‘Process evaluation’ section).

Routinely collected demographic, clinical and recruitment data will include the number of participants who are eligible, willing to be randomised, withdraw after randomisation, complete outcome measurements, reasons for non-completion, age and gender.

### Sample size

In order to detect the minimum clinically important difference of 8 points for the SAQ-UK physical limitation domain, assuming an SD of 25,^15^ with 90% power at the 5% significance level, 207 participants per group will be required (calculated using nQuery 8). In order to allow for up to 20% loss to follow-up, we aim to recruit 259 participants per group (518 in total). This sample size will give >99% power for secondary outcomes.

### Recruitment and randomisation

#### Screening and consent

Written informed consent will be sought from potential participants who will be approached by the study team and invited to consider participation, following a search of their health record for eligibility. Where possible, potential participants will be introduced to the study during a face-to-face appointment with clinical staff. Potential participants can also be approached by a member of the local research team by written invitation and followed up by a phone call or text message after a week if there has been no response. Potential participants will be invited to attend a screening appointment. If they are eligible, and interested in the trial, the trial team researchers would then recruit participants following the trial’s informed consent process. These assessments will be recorded in a screening log, including any reasons for ineligibility. Copies of the participant information sheet and informed consent form can be found in online supplemental appendix 2.

10.1136/bmjopen-2024-084509.supp2 Supplementary data

#### Randomisation procedures

Participants will be randomised via a secure (24-hour) web-based randomisation system controlled centrally by the clinical trials unit to receive either usual care or tailored cardiac rehabilitation programme (‘Activate Your Heart’) plus usual care. Randomisation will have an allocation ratio of 1:1 and use a minimisation programme with a built-in random element, stratified by site and gender. Randomisation should occur no more than 2 weeks after confirming eligibility, obtaining informed consent and completing baseline assessments. Following allocation, participants will be notified of their allocation as soon as possible and then receive their randomised treatment allocation.

#### Blinding

This is a pragmatic trial comparing the ‘Activate Your Heart’ cardiac rehabilitation intervention with usual care, so it will not be possible to blind participants or their rehabilitation providers to treatment group allocation. However, collection of outcome measures and data analysis will be performed blind to treatment allocation. We will include a perception of allocation form for the person collecting the outcome measures to complete, to monitor the level of blinding achieved for these researchers.

### Statistical analysis

Final analysis will take place once all participants have been followed up for 12 months, and the database has been locked. Analyses will be by ‘intention to treat’ for the primary and secondary outcomes on all randomised participants, in the group to which they were allocated, and for whom the outcomes of interest have been observed or measured. A full and detailed statistical analysis plan (SAP) will be finalised prior to any comparative analysis of the groups; the main features of the SAP are described briefly here.

#### Baseline

Demographic and clinical characteristics will be summarised separately using descriptive statistics for each randomised group to allow assessment of whether balance was achieved between randomised groups. This will include gender, ethnicity, socioeconomic status and other relevant PROGRESS-Plus characteristics.^24^ No statistical testing of differences between groups will be performed.

#### Analysis of effectiveness

Primary and secondary outcomes at baseline, 6 months’ and 12 months’ follow-up will be summarised for each treatment group using descriptive statistics at each time point. If normally distributed, the difference between group means (with 95% CIs) will be reported from an analysis of covariance (ANCOVA) adjusted for baseline score and stratification factors.

#### Missing data and withdrawals

Predictors of missing data will be investigated using regression models (including age, living arrangements and comorbidities) and any significant predictors will be considered for inclusion in the models. In addition, given the two assessment points at 6 and 12 months, a sensitivity analysis will be conducted using a joint modelling approach to check whether there is any difference in outcome, using longitudinal outcome rather than the outcome at 6 months or 12 months alone, between the randomised arms, adjusted for dropouts or missing values.

#### Instrumental variable regression

The causal impact of engagement with the intervention will be assessed in an instrumental variable regression model, using the number of stages completed in the rehabilitation programme, and the number of times that the programme is accessed, as measures of intervention use.

#### Mediation analyses

The hypothesised mechanism of change for the rehabilitation intervention is that participants’ primary outcome (physical limitation) is mediated by self-efficacy and physical activity. If the rehabilitation intervention has a significant effect on the primary outcome (p<0.05) in ANCOVA, causal mediation analysis will be used to determine whether these potential mediators predict change in SAQ-UK Physical Limitation domain at 12 months.

### Economic analysis

The economic evaluation aims to estimate the cost-effectiveness of the ‘Activate Your Heart’ online rehabilitation programme plus optimal usual care when compared with optimal usual care alone. The rehabilitation programme will be fully costed using unit costs from a health service perspective. Unit costs will be obtained from national sources of reference costs and applied to information received from pilot questionnaires, namely salary band of cardiac rehabilitation therapists, time spent with the participant at the initial and final rehabilitation appointments, costs of travel and the costs of registering with the ‘Activate Your Heart’ online cardiac rehabilitation programme. Participants’ health service activity data will be obtained from the CSRI^23^ and unit costs from national sources.^25 26^ The costs of service use and the cost of the intervention will be added together for use in a cost-effectiveness analysis. We will account for the likely skewed nature of cost data in any regression carried out, by using Tweedie regression and/or beta distributions. The EQ-5D-5L scores will be converted into a health utility weight using UK norms. The base-case analysis will be conducted using the Van Hout scoring algorithm,^27^ with sensitivity analysis conducted using the EQ-5D-5L value set for England.^28^ Utility indices will be used to calculate QALYs over the 12-month trial period, using the area under the curve approach, with regression-based adjustment for baseline EQ-5D utility scores.^29 30^ The health economic results will initially be presented as disaggregated tables of costs alongside the health consequences. In these tables, we will also describe the costs and consequences of the rehabilitation intervention in the underserved groups recruited, such as women, ethnic minorities and those of low socioeconomic status.

Costs and consequences will then be combined in a cost–utility analysis, to estimate the cost per additional QALY gained associated with each of the compared options. Deterministic and probabilistic sensitivity analyses will be undertaken to explore the robustness of the obtained results to sample variability and plausible variations in key assumptions and employed analytical methods. Cost-effectiveness acceptability curves will be constructed to show the probability that the intervention is cost-effective at specific thresholds of cost per QALY gained such as NICE threshold range of £20 000–£30 000 per QALY gained.^31^ We will develop a two-stage economic model; a decision tree to reflect the outcomes of the trial (within-trial economic evaluation) and a Markov state transition model to extrapolate the evolution of patient outcome and costs over a lifetime horizon (model-based economic evaluation). Value of information analysis will be conducted after taking into account uncertainty derived from decision-analytical models.

### Process evaluation

The concurrent mixed-methods process evaluation will aim to identify and explain the mechanisms and processes that enabled or acted as a barrier to the implementation of the cardiac rehabilitation intervention.^32 33^ The process evaluation will help build a picture of how the intervention was carried out in reality and what factors shaped it. By conducting a process evaluation, it will be possible to identify if observed impacts are solely due to the cardiac rehabilitation programme, or if these impacts are a result of external and internal variables closely linked to the environment and the context in which the intervention takes place. More specifically, the process evaluation will examine the recruitment of sites and rehabilitation teams, recruitment and reach in participants (from the recruitment log), intervention delivery to individuals (coverage, intensity), participation, retention, response of individuals to the rehabilitation intervention (data collected by the ‘Activate Your Heart’ website), unintended consequences (adverse events and inequities), contextual factors and sustained motivation for behaviour change. The website will be examined for the degree of completion of the programme, the number of occasions that the website was accessed and the extent of the change in behaviour(s) recorded. There will be a particular focus on underserved groups (women, ethnic minorities, low socioeconomic status, other PROGRESS-Plus^24^ characteristics and those without internet access or device).

Semistructured telephone or video interviews of participants (n=30) with purposive sampling to ensure diversity based on age, gender, functional impairment, treatment group, site, ethnicity and mode of delivery. A topic guide will be developed with the assistance of our Public Advisory Group and will ask about the acceptability of the rehabilitation programme and the barriers and facilitators of uptake and completion, such as family support, health inequities, lack of facilities, lack of transport, etc. However, topic guides will be iterative and informed by earlier interviews and adapted accordingly. There will be a particular focus on the acceptability and barriers to implementation in underserved groups (women, ethnic minority and low socioeconomic status). Interviews will take place at the end of the 12-week cardiac rehabilitation programme among those receiving the intervention, and between months 11 and 12 in both groups. We will interview a minimum of 10 cardiac rehabilitation staff at different time points, to gain insight into the early, medium-term and longer-term delivery of the cardiac rehabilitation programme. All of the interviews will be audio recorded and fully transcribed. Participant contact with the online discussion forum, and the ‘ask the expert’ email facility will also be examined. We will also examine data collected routinely by the website for the purposes of the national audit for cardiac rehabilitation. Qualitative data will be analysed using a thematic analysis approach.^34^ Quantitative data (recruitment logs, use of the website, recorded change in behaviours, national audit data) will be analysed using descriptive statistics to allow for the exploration of frequency of responses. All data sets will be synthesised in order to describe the complex nature of the enhanced rehabilitation intervention.

### Patient and public involvement

There has been patient and public involvement (PPI) at all stages including developing the research question, choosing outcomes relevant to patients, commenting on the burden of the intervention and of trial participation. Two PPI coinvestigators are active members of the trial management group and help to convene a public advisory group which meets throughout the trial to advise on trial procedures, especially patient-facing materials, promotional videos in multiple languages and the dissemination plan. Members of the PPI group recorded voice-overs for the promotional video in different languages.

### Ethics and dissemination

NHS research ethics approval was obtained from North of Scotland Research Ethics Committee, reference 21/NS/0116. The current protocol is version 5 (4 May 2023). Participants will provide written informed consent. A trial steering committee is providing overall supervision, and an independent data safety and monitoring committee is responsible for reviewing and assessing recruitment, interim monitoring of safety and effectiveness, trial conduct and external data.

All safety events will be recorded by researchers when they are made aware of the event by the participant, treating clinician or therapist. Adverse event reports and serious adverse events (SAEs) not related to the intervention will be entered on to the remote data entry system. Each SAE will be assessed by the relevant PI to determine whether it is related to the intervention. A related SAE will be assessed by the CI to determine whether it is expected. If the SAE is related and unexpected it will be reported to the research ethics committee and sponsor in an expedited manner.

Reporting of the trial will be consistent with the Consolidated Standards for Reporting Trials (CONSORT) 2010 Statement (patient-reported outcomes and non-pharmacological interventions).^35^ We will submit the final report to a peer-reviewed academic journal, according to our publication strategy and authorship policy. Research data will be available for secondary analysis on reasonable request.

### Trial status

The first participant was recruited in July 2022. At the time of submission this trial had been open in 20 sites, with 10 more sites in preparation and had recruited 68 participants. Updated recruitment information can be found on the trial website (http://activate-trial.org.uk).

## Supplementary Material

Reviewer comments

Author's manuscript
