# Supplementary material for: Effectiveness and cost-effectiveness of a web-based cardiac rehabilitation programme for people with chronic stable angina: protocol for the ACTIVATE (Angina Controlled Trial Investigating the Value of the ‘Activate your heart’ Therapeutic E-intervention) randomised controlled trial
Source: BMJ Open. 2024 Mar 25;14(3):e084509. doi: 10.1136/bmjopen-2024-084509 (PMC10966821; doi:10.1136/bmjopen-2024-084509)
Supplement: Supplementary data [file bmjopen-2024-084509supp001.pdf]

Appendix 1 ACTIVATE Trial Registration Data

Data category	Information
Registry and trial identification no.	ISRCTN10054455
Date of registration	16/09/2021
Funder	NIHR Evaluation, Trials and Studies Co-ordinating Centre (NETSCC); Grant code 131015
Sponsor	University of Liverpool
Contact for public enquiries	email: activate.trial@liverpool.ac.uk
Scientific title	Cardiac rehabilitation for people with chronic stable angina
Acronym	Angina Controlled Trial Investigating the Value of the 'Activate Your Heart' E-intervention (ACTIVATE)
Countries of recruitment	United Kingdom
Health condition	Angina pectoris
Intervention	Intervention comparator: Web-based cardiac rehabilitation ('Activate Your Heart')
	Control comparator: Usual care
Inclusion criteria	Adult patients (age >18 years)
	Chronic stable angina with at least 2 out of 3 of the following features:<br>i) Constricting central chest pain<br>ii) Precipitated by exertion or emotional stress<br>iii) Relieved by rest or glyceryl trinitrate spray
	Evidence of myocardial ischaemia from either a past medical history of acute coronary syndrome, myocardial infarction (MI) or revascularisation procedure at least 12 months in the past; or from imaging studies such as invasive coronary angiography, computerised tomography angiography or myocardial perfusion testing.
	Revascularisation procedures not planned and treated with medical treatments only, including people with previous MI, or previous revascularisation procedure who may have attended cardiac rehabilitation in the past.
Exclusion criteria	History of myocardial infarction (MI) within the last 12 months.
	History of revascularisation procedure within the last 12 months, or planned at the time of study recruitment.
	Participation in a cardiac rehabilitation programme within the last 12 months, or planned at time of study recruitment.

	Significant co-morbidities (as deemed by person confirming eligibility) that would limit participation in the exercise-based rehabilitation programme.
	Refractory angina on maximal medical therapy.
Study design	Interventional
	Randomised controlled trial
	Treatment, education or self-management, psychological and behavioural, complex intervention, cardiac rehabilitation
Recruitment start date	01/12/2021
Target sample size	518
Primary outcome	UK version of Seattle Angina Questionnaire (SAQ-UK), physical limitations domain
Secondary outcomes	SAQ-UK angina frequency and perception and treatment satisfaction domains, MRC dyspnoea scale, Hospital Anxiety and Depression Scale, General Self-Efficacy Scale. Physical activity measured with the ActivPAL accelerometer for 7 days, supplemented by the International Physical Activity Questionnaire. Cardiovascular fitness measured with the Incremental Shuttle Walk Test. Economic measures EuroQol EQ-5D-5L and Client Service Receipt Inventory.
