# Supplementary material for: Effectiveness and cost-effectiveness of a web-based cardiac rehabilitation programme for people with chronic stable angina: protocol for the ACTIVATE (Angina Controlled Trial Investigating the Value of the ‘Activate your heart’ Therapeutic E-intervention) randomised controlled trial
Source: BMJ Open. 2024 Mar 25;14(3):e084509. doi: 10.1136/bmjopen-2024-084509 (PMC10966821; doi:10.1136/bmjopen-2024-084509)
Supplement: Supplementary data [file bmjopen-2024-084509supp002.pdf]

<Trust/Site address 1>
<Trust/Site address 2>
<Trust/Site address 3>
<postcode>
Tel: <telephone number>

Participant Information Sheet for ACTIVATE

- You have been invited to take part in a research study. Before you decide, it is important that you understand why the research is being done and what it will involve.
- Please take time to read the following information carefully. **Part 1** tells you the purpose of the study and what will happen to you if you take part. **Part 2** gives you more detailed information about the conduct of the study.
- You can ask a member of your clinical team if there is anything that is not clear, or if you would like more information.
- If you wish you can discuss it with friends, relatives or get independent advice from your local Patient Advice and Liaison Service (PALS) or equivalent.
- **Taking part is voluntary**. You do not need to take part and you do not need to give a reason.
- ACTIVATE is a study to look at cardiac rehabilitation for people with long-term stable angina (chest pain that happens with exertion) to see if a cardiac rehabilitation programme is effective.

How to contact the study team:

If you have any questions about this study please talk to your research team:

- **Principal investigator name and Tel number**
- **Research Nurse name and Tel Number**

Contents:

Part 1

- Why are we doing the ACTIVATE study?
- Do I have to take part?
- What will happen to me if I take part?
- What will I have to do if I take part?
- How will I know which treatment I'm going to have?
- What are the benefits and risks of taking part?
- What are the alternatives for treatment?
- What happens if I change my mind?
- What if new information becomes available?
- What happens when the study stops?
- What if there is a problem?
- Will my taking part in the study be kept confidential?

Part 2

- Who is running the study?
- How will my information be collected and handled?
- What are my choices about how my information is used?
- Information sharing for other research
- Where can I find out more about how my information is used?
- What if there is a problem?

Williams NH, *et al. BMJ Open* 2024; 14:e084509. doi: 10.1136/bmjopen-2024-084509

PART 1: Purpose of the study and what will happen if you take part

Why are we doing the ACTIVATE study?

The aim of the study is to improve the health of people with long-term angina which limits daily activities. We want to see whether a rehabilitation programme combined with usual heart care is better than usual heart care alone.

Angina is chest pain brought on by physical activity or stress and is caused by narrowed blood vessels restricting the flow of blood to the heart muscle. It can severely limit day to day activities. Cardiac rehabilitation programmes promote the physical and mental health of people with heart disease by increasing their understanding and improving their physical fitness levels. Cardiac rehabilitation is normally recommended after heart attacks or heart surgery, but not for people with stable angina who do not require an operation. Although cardiac rehabilitation might also benefit people with stable angina, more evidence is needed before it can be used in standard care.

Participants will be split into two groups randomly. Group A will receive usual care from your healthcare provider plus a cardiac rehabilitation programme called 'Activate Your Heart'. Group B will receive usual care from your healthcare provider. 'Activate Your Heart' is an interactive website for you to use at home. The programme was developed by a team of health care professionals, people with angina and software designers. The programme helps you to set goals, monitor progress and gives you feedback on how you are doing. It encourages you to gradually increase your activity level. A paper manual will be available for participants who are unable to use the online version or for those who feel they would prefer a paper version. We would also like participants to complete some questionnaires whilst taking part in the study and also wear a small device called an ActivPAL device which looks out how active you are.

We want to recruit 518 participants aged 18+ years with long-term stable angina. Each participant will be asked to take part in the study for 12 months.

Do I have to take part?

No, **taking part is voluntary**. It is up to you to decide whether or not you want to take part.

If you decide not to take part then you will still receive the usual treatment your healthcare provider offers.

If you decide to take part you can also choose to stop at any time without giving a reason. The decision you make on whether to take part or not will not affect the standard of care you receive now or in the future.

What will happen to me if I take part?

A member of the clinical team can talk to you in more detail about this study and you will be able to ask any questions that you have. If you have had all of your questions answered and are happy to take part then you will be asked to sign a consent form to confirm you want to take part.

Whichever group you are allocated, we will ask you to attend the visits shown in the flowchart on page 3.

What will I have to do if I take part?

Once you have signed the consent form, we will check and confirm that this study is suitable for you and you will be asked to follow the study plan (see study timeline on page 3). You will have to:

- Attend your GP surgery or hospital for follow-up visits over a 12-month period. It may be possible to do these over the telephone or video call.
- At each follow-up visit we will ask you to complete a set of questionnaires.
- If you are randomly allocated to receive the 'Activate Your Heart' intervention you will be invited to meet with someone who will explain how the system works. A member of the research team will contact you to arrange a suitable date to meet and give you instructions on where to meet.

Williams NH, *et al. BMJ Open* 2024; 14:e084509. doi: 10.1136/bmjopen-2024-084509

Study Timeline

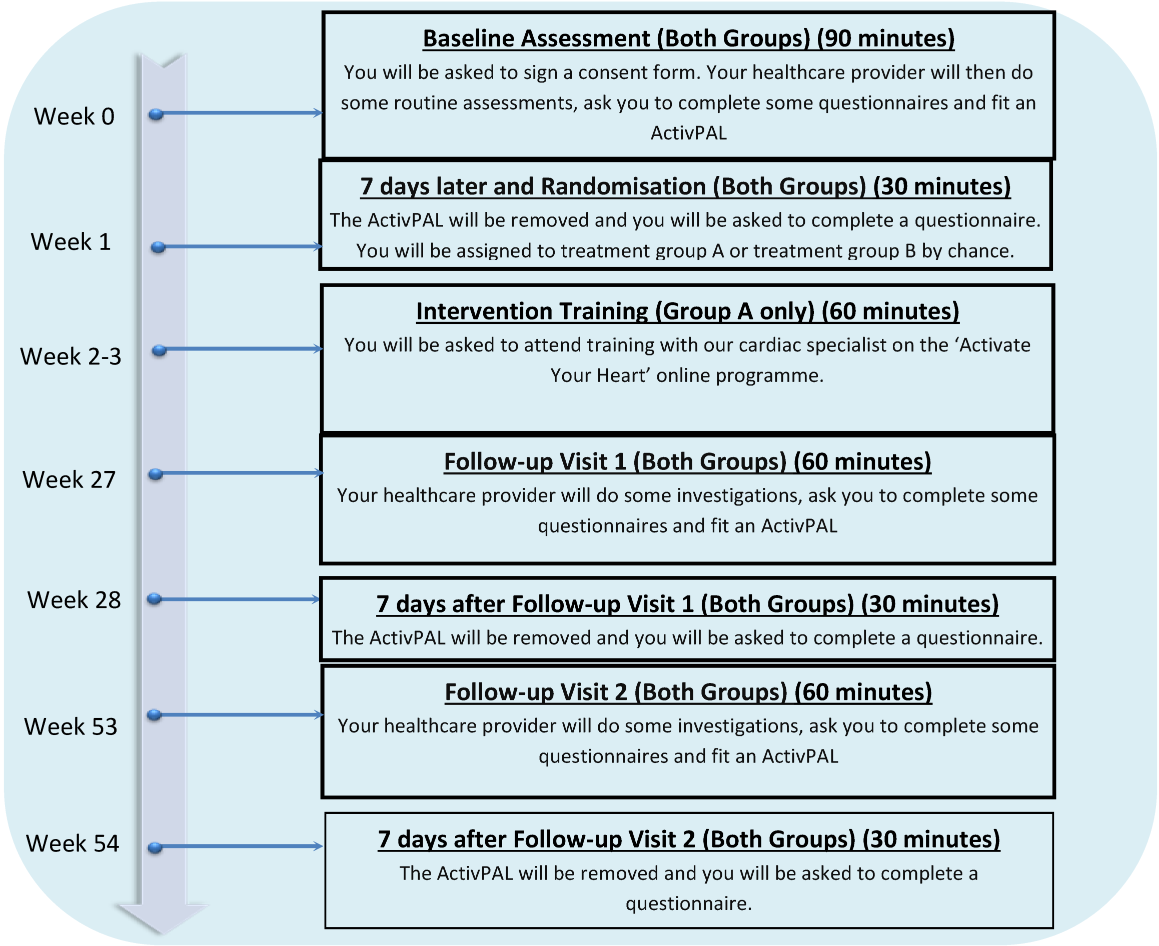

**Procedure**	**Description**	**Research Treatment or Standard of care**
Activate your heart	This is a secure, interactive website designed for you to use at home. The programme is tailored to individual need and is in four stages, which can be completed in 8 weeks, but your access to the site and its features continue for 12 months. A paper version is also available for any participant who does not want to access the website.	**Research Treatment**
Questionnaires	We will ask you to complete a set of questionnaires about your health, and use of health services, when you start taking part, again after 6 months and then a final set of questionnaires at 12 months.	**Research Treatment**
Physical activity measured with the ActivPAL accelerometer	The ActivPAL device is a small, slim monitor worn on the thigh. We will ask you to wear it for 7 days when you first take part, for another 7 days after 6 months and then a final 7 days after 12 months. The device will look at how physically active you are. We will also ask you to keep a diary documenting working hours, sleep, reasons for not wearing it, and any other comments	**Research Treatment**

Williams NH, *et al. BMJ Open* 2024; 14:e084509. doi: 10.1136/bmjopen-2024-084509

Procedure	Description	Research Treatment or Standard of care
Incremental Shuttle Walk Test	We will ask you to complete this test when you start taking part, again after 6 months and then finally at 12 months. For this walking test we will ask you to walk back and forth along a 10-metre track at a comfortable pace, every minute the pace will increase. The distance you can walk will give us an idea of your exercise capacity.	**Research Treatment**
Height, weight, blood pressure and Serum Cholesterol	These are standard tests that you will have done when you visit the Doctor, we would like to collect this information for our study.	**Standard of Care**

Optional telephone interviews

We will interview a selection of participants over the telephone to ask about your experience of being in the study. These will last up to 1 hour and will be recorded. We will offer a £30 shopping voucher for your time.

How will I know which treatment I get?

In the ACTIVATE study participants will be split into two treatment groups. Your healthcare provider will let you know what group you are in. We use a computer programme that puts participants into groups by chance:

- One group will receive standard of care treatment plus have access to the online 'Activate Your Heart' rehabilitation programme.
- The other group will receive standard of care treatment only.

Are there any risks of taking part?

Experience from using the 'Activate Your Heart' programme for cardiac rehabilitation has shown that it is very safe. There may be a small risk of injury or provoking an episode of angina when exercising, but to prevent this, the physical activity goals are carefully set. You can contact the cardiac rehabilitation therapists for advice and support or by joining an online scheduled weekly chat room.

What happens if I change my mind?

If at any point you decide to stop taking part in the study you will still receive your usual treatment from your healthcare provider. If you do decide to stop taking part we will ask you if you would like to: continue to complete follow up visits for the study or stop taking part with no more study visits.

What happens when the study stops?

When you finish taking part in the study your healthcare provider will discuss what treatment is best for you next.

The results of the study will be presented at conferences and published in medical journals. Confidentiality will be ensured at all times and you will not be identified in any publication.

Any information resulting directly or indirectly from this research, as well as any patents developed directly or indirectly as a result of this research may be used for commercial purposes. You have no right to this property or to any share of the profits that may be earned directly or indirectly as a result of this research. However, in signing this form for this research, you do not give up any rights that you would otherwise have as a participant in research.

What if there is a problem?

We don't expect there to be any problems the study has been carefully designed to ensure your safety is the main priority. Any complaint about the way you have been dealt with during the study or any possible harm you might suffer will be addressed. See Part 2 below.

Will my taking part be kept confidential?

Williams NH, *et al. BMJ Open* 2024; 14:e084509. doi: 10.1136/bmjopen-2024-084509

Yes. All the confidential information about your participation in this study will be kept confidential. Detailed information on this is given in Part 2.

Williams NH, *et al. BMJ Open* 2024; 14:e084509. doi: 10.1136/bmjopen-2024-084509

PART 2: Detailed Information about the conduct of the study

Who is running the study?

The University of Liverpool is the Sponsor of this study and is responsible for managing it. They have asked that the day to day running of the study is carried out by a team based at the Liverpool Clinical Trials Centre (LCTC, part of the University of Liverpool) and qualitative and health economic researchers (part of the University of Liverpool).

The study has been reviewed by the Health Research Authority and the North of Scotland Research Ethics Committee (2).

This study is funded by the National Institute for Health Research's Health Technology Assessment Programme (ref: NIHR: 131015).

Your healthcare provider will not receive any payment for including you in this study.

How will my information be collected and handled?

The University of Liverpool is the Data Controller for this study and will need to use information from you and your medical records for this research project.

This information will include your initials, NHS number and name. If you are in the rehabilitation group (Group A of the study), your contact details will be sent to the cardiac rehabilitation team, so that they can contact you to arrange an appointment to explain how to use the 'Activate Your Heart' programme. Your contact details will also be shared with qualitative researchers at The University of Liverpool so they can contact you for telephone/video-call interviews. People will use this information to do the research or to check your records to make sure that the research is being done properly.

Individuals from The University of Liverpool, the LCTC and regulatory organisations may look at your medical and research records to check the accuracy of the research study.

People who do not need to know who you are will not be able to see your name or contact details. Your data will have a code number instead.

Data will be entered into a secure database from your healthcare team and staff members at the LCTC will be able to see this data. The LCTC will share some data with qualitative researchers and health economics researchers. The researchers doing the interviews will contact 30 participants about 3 months after the start of the study and again after 12 months to ask questions about how they felt being part of the study. These interviews can take place on the telephone or video call.

Health economic researchers at The University of Liverpool want to use your health economic data (number of visits to hospital, medication taken etc.) to help calculate the overall costs of care. We will ask you to complete a questionnaire called Client Service Receipt Inventory (CSRI). This is a tool used to collect information on the whole range of services and supports study participants may use. We will share the information you provide in this questionnaire with health economic researchers at The University of Liverpool.

We will keep all information about you safe and secure.

Once we have finished the study, we will keep the data for 10 years, so we can check the results. We will write our reports in a way so that no-one can work out that you took part in the study.

You can find out more about how we use your information at www.activate-trial.org.uk

What are my choices about how my information is used?

You can stop being part of the study at any time, without giving a reason, but we will keep information about you that we already have.

Williams NH, *et al. BMJ Open* 2024; 14:e084509. doi: 10.1136/bmjopen-2024-084509

If you choose to stop taking part in the study, we would like to continue collecting information about your health from central NHS records, your hospital and your GP. If you do not want this to happen, please tell your healthcare provider

If you lose capacity during the study we would still plan to use the information collected from you.

We need to manage your records in specific ways for the research to be reliable. This means that we will not be able to let you see or change the data we hold about you.

Information sharing for other research

When you agree to take part in a research study, the information about your health and care may be beneficial to researchers running other research studies in this organisation and in other organisations. These organisations may be universities, NHS organisations or companies involved in health and care research in this country or abroad. Your information will only be used by organisations and researchers to conduct research in accordance with the UK Policy Framework for Health and Social Care Research, or equivalent standards.

If you agree to take part in this study, you will have the option to take part in future research using your anonymous data saved from this study.

Where can I find out more about how my information is used?

You can find out more about how we use your information:

- at the study website: www.activate-trial.org.uk
- at www.hrs.nhs.uk/information-about-patients
- in the Health Research Authority leaflet available from www.hra.nhs.uk/patientdataandresearch
- by contacting the University of Liverpool Data Protection Officer on LegalServices@liverpool.ac.uk.
- by asking one of the research team

What if there is a problem?

If you have a concern about any aspect of this study, you should ask to speak with one of your research team who will do their best to answer your questions.

If you remain unhappy and wish to complain formally, you can do this by contacting local NHS Patient Advice and Liaison Service (PALS) or equivalent. Members of your local hospital team or GP should be able to provide this information to you.

Every care will be taken in the course of this study. However, in the unlikely event that you are harmed by taking part in this research project, compensation from the study Sponsor (The University of Liverpool) may be available, but you may have to pay your related legal costs. The place where you receive your treatment, has a duty of care to you whether or not you agree to participate in the study. The study Sponsor accepts no liability for negligence on the part of your GP practice or healthcare provider. However, if you are harmed, and this is due to someone's negligence at the GP practice or healthcare provider, then you may have grounds for a legal action for compensation against the NHS organisation where you are being treated but you may have to pay for your legal costs. The normal National Health Service complaints procedures should be available to you.

FUNDED BY NIHR | National Institute for Health Research

Thank you for taking the time to read and consider this information sheet. Should you decide to take part in the study, you will be given a copy of the information sheet and a signed consent form to keep.

ACTIVATE

<Trust/Site address 1>
<Trust/Site address 2>
<Trust/Site address 3>
<postcode>
Tel: <telephone number>

Participant Consent Form

FOR SITE USE ONLY:

Site Name:	
Participant Study Number	
Participant Initials:	Participant DOB: / /

To be completed by the participant:

Once you have read and understood each statement please enter your initials in each box.	Initial
1. I have read and understood the information sheet for this study. I have had the opportunity to ask questions and have had these answered satisfactorily.	
2. I understand that participation is voluntary and that I am free to withdraw from the study at any time, without giving a reason, and without my care or legal rights being affected. However, the study team may need to collect some limited information for safety reasons.	
3. I give permission for a copy of this fully completed consent form to be sent to the LCTC (where it will be kept in a secure location) to allow confirmation that my consent was given.	
4. I understand that relevant sections of my medical notes and any data collected during the study may be looked at by authorised individuals from the central study team and representatives of the Sponsor, regulatory authorities and my healthcare provider. I give permission for these individuals to have access to my records and data.	
5. I agree to my GP being informed of my participation in the study.	
6. I agree for my data collected on the Client Service Receipt Inventory (CSRI) form to be shared with researchers at The University of Liverpool for the purpose of health economic research.	
7. I understand that my data will be kept by the University of Liverpool and at my healthcare provider in a confidential manner for 10 years from the end of the study.	
8. I agree to take part in the above study.	
The statements below are optional (you can still take part in the study even if you do not wish to agree to these):	
9. I agree to allow information or results arising from this study to be used in future healthcare and/or medical research providing my confidentiality is maintained.	
10. I agree to being contacted by a study researcher to conduct a qualitative interview and for the interview to be recorded. (if you agree to this statement provide your details below):	
11. I agree for any of my feedback from the qualitative interviews to be used as quotes in future publications, presentations and on the study website and newsletters	

Telephone number:	
Email address	

Please continue to the next page.

Williams NH, *et al. BMJ Open* 2024; 14:e084509. doi: 10.1136/bmjopen-2024-084509

ACTIVATE

<Trust/Site address 1>
<Trust/Site address 2>
<Trust/Site address 3>
<postcode>
Tel: <telephone number>

Participant Consent Form

FOR SITE USE ONLY:

Site Name:			
Participant Study Number			
Participant Initials:		Participant DOB:	/ /

To be completed by the participant:

Your full name (please print):			
Your signature:		Date:	

To be completed by the Researcher (after participant has completed the form):

Researcher full name (please print):			
Researcher signature:		Date:	

Please file the original wet-ink copy in the ACTIVATE Investigator Site File, and make three copies: one for the participant, one for the medical notes and one to be sent to the LCTC.

Williams NH, *et al. BMJ Open* 2024; 14:e084509. doi: 10.1136/bmjopen-2024-084509
