# Supplementary material for: Effectiveness and cost-effectiveness of a web-based cardiac rehabilitation programme for people with chronic stable angina: protocol for the ACTIVATE (Angina Controlled Trial Investigating the Value of the ‘Activate your heart’ Therapeutic E-intervention) randomised controlled trial
Source: BMJ Open. 2024 Mar 25;14(3):e084509. doi: 10.1136/bmjopen-2024-084509 (PMC10966821; doi:10.1136/bmjopen-2024-084509)
Supplement: Author's manuscript [file bmjopen-2024-084509.draft_revisions.pdf]

BMJ Open is committed to open peer review. As part of this commitment we make the peer review history of every article we publish publicly available.

When an article is published we post the peer reviewers' comments and the authors' responses online. We also post the versions of the paper that were used during peer review. These are the versions that the peer review comments apply to.

The versions of the paper that follow are the versions that were submitted during the peer review process. They are not the versions of record or the final published versions. They should not be cited or distributed as the published version of this manuscript.

BMJ Open is an open access journal and the full, final, typeset and author-corrected version of record of the manuscript is available on our site with no access controls, subscription charges or pay-per-view fees (http://bmjopen.bmj.com).

If you have any questions on BMJ Open's open peer review process please email info.bmjopen@bmj.com

BMJ Open

Protocol for a randomised controlled trial of cardiac rehabilitation for people with chronic stable angina: Angina Controlled Trial Investigating the Value of the 'Activate your heart' Therapeutic E-intervention (ACTIVATE) [ISRCTN10054455]

Journal:	*BMJ Open*
Manuscript ID	bmjopen-2024-084509
Article Type:	Protocol
Date Submitted by the Author:	20-Jan-2024

Complete List of Authors:	Williams, Nefyn; University of Liverpool, Primary Care and Mental Health<br>Collins, Brendan; University of Liverpool, Public Health Policy and Systems<br>Comerford, Terence; National Institute for Health and Care Research Applied Research Collaboration North West Coast, Patient and Public Involvement<br>Dodd, Susanna; University of Liverpool, Biostatistics<br>Fisher, Michael; Liverpool University Hospitals NHS Foundation Trust<br>Hardwick, Ben; University of Liverpool, Liverpool Clinical Trials Centre<br>Hennessy, Sophie; University of Liverpool Faculty of Health and Life Sciences, Liverpool Clinical Trials Centre<br>Jolly, Kate; University of Birmingham, Institute of Applied Health Research<br>Jones, Ian; Liverpool John Moores University,<br>Lane, Deirdre; University of Liverpool,<br>Lip, Gregory; University of Liverpool, Liverpool Centre for Cardiovascular Science<br>Morgan, Erica; National Institute for Health and Care Research Applied Research Collaboration North West Coast, Patient and Public Involvement<br>Ralph, Penelope; University of Liverpool Faculty of Health and Life Sciences, Department of Primary Care and Mental Health<br>Thijssen, Dick; Liverpool John Moores University<br>Singh, Sally; University Hospitals of Leicester NHS Trust, Cardiac/Pulmonary Rehabilitation

Keywords:	Ischaemic heart disease < CARDIOLOGY, Primary Care < Primary Health Care, REHABILITATION MEDICINE, Randomized Controlled Trial, Adult cardiology < CARDIOLOGY, HEALTH ECONOMICS

SCHOLARONE™ Manuscripts

For peer review only - http://bmjopen.bmj.com/site/about/guidelines.xhtml

Protocol for a randomised controlled trial of cardiac rehabilitation for people with chronic stable angina: Angina Controlled Trial Investigating the Value of the 'Activate your heart' Therapeutic E-intervention (ACTIVATE) [ISRCTN10054455]

Nefyn H Williams[1,6]

Email: Nefyn.williams@liverpool.ac.uk

Brendan Collins[1]

Email: brenc@liverpool.ac.uk

Terence Comerford[2]

Email: tjc57b@gmail.com

Susanna Dodd[1]

Email: s.r.dodd@liverpool.ac.uk

Michael Fisher[3,6]

Email: Michael.Fisher@liverpoolft.nhs.uk

Ben Hardwick[1]

Email: ben121@liverpool.ac.uk

Sophie Hennessy[1]

Email: Sophie.hennessy@liverpool.ac.uk

Kate Jolly[4]

Email: c.b.jolly@bham.ac.uk

Ian Jones[5,6]

Email: i.d.jones@ljmu.ac.uk

Deirdre A Lane[1,6]

Email: deirdre.lane@liverpool.ac.uk

Gregory YH Lip[6]

For peer review only

Email: gregory.lip@liverpool.ac.uk

Erica Morgan[2]

Email: ericamorgan@me.com

Penelope Ralph[1]

Email: p.ralph@liverpool.ac.uk

Dick Thijssen[5,6]

Email: D.Thijssen@ljmu.ac.uk

Sally J Singh[7]

Email: sally.singh@uhl-tr.nhs.uk

[1]University of Liverpool, UK

[2]National Institute for Health and Care Research (NIHR), Applied Research Collaboration North West Coast (ARC NWC), Patient and Public Involvement

[3]Liverpool University Hospitals NHS Foundation Trust, UK

[4]University of Birmingham, UK

[5]Liverpool John Moores University, UK

[6]Liverpool Centre for Cardiovascular Science at University of Liverpool, Liverpool John Moores University and Liverpool Heart and Chest Hospital, UK

[7]Department of Respiratory Sciences, University of Leicester, Leicester, UK.

Corresponding author: Professor Nefyn H Williams, Department of Primary Care and Mental Health, University of Liverpool, 1st Floor, Block B, Waterhouse Building, 1-5 Brownlow Street, Liverpool, L69 3GL, UK.

Tel: +44 (0)151 795 5305 e-mail: nefyn.williams@liverpool.ac.uk

For peer review only - http://bmjopen.bmj.com/site/about/guidelines.xhtml

Abstract

Introduction

Chronic stable angina is common, and disabling. Cardiac rehabilitation is routinely offered to people following myocardial infarction or revascularisation procedures, and has the potential to help people with chronic stable angina. However, there is insufficient evidence of effectiveness and cost-effectiveness for its routine use in this patient group.

The objectives are to compare the effectiveness and cost-effectiveness of the 'Activate Your Heart' cardiac rehabilitation programme for people with chronic stable angina compared with usual care.

Methods and analysis

Protocol for multisite, parallel-group, two-armed, superiority, pragmatic randomised controlled trial with 1:1 allocation. Allocation sequence by minimisation programme with a built in random element. Secure web-based allocation concealment. The two treatments will be optimal usual care (control) and optimal usual care plus the 'Activate Your Heart' web-based cardiac rehabilitation programme (intervention). Outcome assessment and statistical analysis will be performed blind; participants will be unblinded. Outcomes will be measured at baseline, 6 and 12 months' follow-up. Primary outcome will be the UK version of Seattle Angina Questionnaire (SAQ-UK), physical limitations domain at 12 months' follow-up. Secondary outcomes will be the remaining two domains of SAQ-UK, dyspnoea, anxiety and depression, health utility, self-efficacy, physical activity and the incremental shuttle walk test. All safety events will be recorded, and serious adverse events will be assessed to determine whether they are related to the intervention and expected.

Concurrent economic evaluation will be a cost-utility analysis from a health service perspective. An embedded process evaluation will determine the mechanisms and

For peer review only - http://bmjopen.bmj.com/site/about/guidelines.xhtml

processes that explain the implementation and impacts of the cardiac rehabilitation programme.

Ethics and dissemination

NHS research ethics approval reference 21/NS/0115. Results will be disseminated by peer-reviewed publication.

Registration details

ISRCTN10054455 registered on 16/09/2021.

Article Summary

Strengths and limitations of this study

- Pragmatic phase III randomised controlled trial of an established cardiac rehabilitation programme for individuals with chronic stable angina.
- Concurrent economic evaluation with a health service perspective.
- Embedded process evaluation to determine the mechanisms and processes that explain the implementation and impacts of the enhanced rehabilitation programme.
- The web-based intervention may not be acceptable to some people, but a paper version is available.

For peer review only - http://bmjopen.bmj.com/site/about/guidelines.xhtml

Introduction

Angina is chest pain usually caused by atherosclerosis of the coronary arteries, which restricts blood flow to the myocardium, especially when oxygen demand is increased during exercise. Angina is defined as stable if chest pain occurs predictably on physical exertion, or with emotion, and settles promptly with rest or after taking sublingual nitrate medication. Affected individuals have an increased risk of acute coronary events such as myocardial infarction (MI) [1]. Management consists of behaviour change advice to reduce risk factor profile, drug treatment and revascularisation procedures [2,3].

Cardiac rehabilitation is routinely offered to patients following MI, or revascularisation procedures, and consists of co-ordinated activities to improve the underlying cause and restore optimal functioning and slow, or reverse, disease progression [4]. The most prevalent components of cardiac rehabilitation are behaviour change and exercise training, but comprehensive cardiac rehabilitation programmes also include education, interventions to improve mental health, involvement of friends and family members and the development of long-term strategies beyond participation in the cardiac rehabilitation programme. Despite international guidance [2,5], such programmes are not offered routinely to people with chronic stable angina [6]. In the UK, the National Institute for Health and Care Excellence (NICE) does not support cardiac rehabilitation in this group until stronger evidence of effectiveness and cost-effectiveness is available [3]. Although there is good evidence for the effectiveness of cardiac rehabilitation for heart failure [7], or following MI or revascularisation procedures [8], rates of uptake and long-term adherence are low, especially in women, ethnic minority groups, and in areas of high deprivation [6].

For peer review only - http://bmjopen.bmj.com/site/about/guidelines.xhtml

A systematic review of exercise-based cardiac rehabilitation for adults with stable angina [9], identified seven randomised controlled trials (RCTs) (n=581) with high risk of bias. It found low quality evidence that cardiac rehabilitation resulted in a small improvement in exercise capacity compared with usual care with standard mean difference 0.45 (95% confidence intervals [CI] 0.2 to 0.7), and uncertainty about the effect on all-cause mortality, acute MI, cardiovascular-related hospital admissions, quality of life (QoL) and cardiac rehabilitation-related adverse events. These RCTs were small in size and recruited mainly white middle-aged men. More evidence is needed from under-represented groups including women, older age-groups, ethnic minorities and those with co-morbid conditions. Web-based programmes might increase the reach of cardiac rehabilitation, and there has been one small RCT (n=94) of such a programme ('Activate Your Heart'), which demonstrated that it was possible to provide cardiac rehabilitation to people with chronic stable angina [10]. Trial methods were feasible in terms of recruitment, retention and outcome measurement. After six months there were statistically significant lower levels of angina frequency, but a larger RCT is needed to test longer-term effectiveness and cost-effectiveness.

‘Activate Your Heart’ has the potential to improve physical and mental health with minimal risk. There may be a risk of injury or provoking an episode of angina when exercising, however the physical activity goals are carefully graded and the programme has an excellent safety record [10].

For peer review only - http://bmjopen.bmj.com/site/about/guidelines.xhtml

Aims and objectives

We aim to assess whether a web-based cardiac rehabilitation programme can improve the health of people with chronic stable angina, whether it is good value for money and delivered as intended.

The primary objective is to determine the effectiveness of the 'Activate Your Heart' web-based cardiac rehabilitation programme (or a paper version for those who prefer or who do not have internet access or device), for people with chronic stable angina compared with usual care in terms of angina-related health status, at 12 months' follow-up.

Secondary Objectives:

- To estimate the cost-effectiveness of the cardiac rehabilitation programme for people with chronic stable angina compared with usual care in a cost-utility analysis with a health service perspective;
- To investigate the mechanisms and processes that explain the implementation and impacts of the cardiac rehabilitation programme in the intervention group and usual care in the control group;
- To identify the barriers and facilitators of uptake and completion of the rehabilitation programme with a focus on equity.

Methods and Analysis

Trial design

For peer review only - http://bmjopen.bmj.com/site/about/guidelines.xhtml

Multisite, parallel-group, two-armed, superiority RCT with 1:1 allocation, stratified by gender and recruitment site (Figure 1). A pragmatic RCT of effectiveness compared with usual care, with blinded outcome assessment and statistical analysis; unblinded participants and rehabilitation providers. An internal pilot phase will assess feasibility. There will be a concurrent economic evaluation with a health service perspective. An embedded process evaluation will use mixed methods to examine the mechanisms and processes that explain the implementation and impacts of the enhanced rehabilitation programme, including an examination of barriers leading to health inequities. The Health Inequalities Assessment Toolkit (www.hiat.org.uk) informed trial design. The RCT was registered on 16 September 2021, ISRCTN10054455. Trial registration data can be found in Appendix 1 in the supplementary file.

For peer review only

Trial Setting and Selection of Sites / Clinicians

Participants will be recruited from primary and secondary care in areas of England and Wales with high disease burden and socio-economic deprivation. Many of these areas include high proportions of ethnic minority groups. The web-based rehabilitation intervention (or paper version) will be delivered in the community.

Trial Population

Inclusion criteria

- Adults with chronic stable angina with at least two out of three of the following features: constricting central chest pain, precipitated by exertion or emotional stress, relieved by rest or glyceryl trinitrate spray.

For peer review only - http://bmjopen.bmj.com/site/about/guidelines.xhtml

- Evidence of myocardial ischaemia from either a past medical history of acute coronary syndrome, myocardial infarction (MI) or revascularisation procedure at least 12 months previously; or from imaging studies such as invasive coronary angiography, computerised tomography angiography or myocardial perfusion testing.
- Revascularisation procedures not planned and managed with medical treatments only, including people with previous MI, or previous revascularisation procedure who may have attended cardiac rehabilitation in the past.

Exclusion criteria

- History of MI, revascularisation or cardiac rehabilitation participation within the last 12 months.
- Significant co-morbidities (as deemed by person confirming eligibility) that would limit participation in the exercise-based rehabilitation programme.
- Refractory angina on maximal medical therapy.

Trial treatment/interventions

We plan to compare web-based cardiac rehabilitation in addition to optimal usual care (intervention) with optimal usual care alone.

Optimal usual care

Usual care will be optimised in all participants. All participants will be seen by their GP or cardiology team, who will use a protocol informed by NICE guidance [3] to ensure that all are receiving optimal care. Considering the pragmatic nature of the proposed RCT, no

For peer review only - http://bmjopen.bmj.com/site/about/guidelines.xhtml

restrictions will be placed on usual care. NICE guidance [3] recommends that patients with chronic stable angina receive adequate information and support, lifestyle advice about smoking, exercise, diet and weight control. Episodes of angina should be treated with short-acting nitrate medication. Anti-anginal drug treatment to prevent episodes of angina consist of a beta-blocker or calcium channel blocker in the first instance; second line drugs (such as ivabradine, ranolazine) will be used as needed. In addition, drugs for secondary prevention of cardiovascular disease such as MI or stroke, include aspirin, angiotensin-converting enzyme inhibitor (especially if diabetes is present), statins in line with NICE guidance (and treatment targets) on lipid modification [11], and anti-hypertensives in line with NICE guidance (and treatment targets) on hypertension [12].

Concomitant medication will be recorded as part of the health economic data collection. There are no restrictions on any medications. Participants will not have participated in a cardiac rehabilitation programme over the previous 12 months, but other schemes such as smoking cessation, exercise referral and weight management schemes may be available and accessible to participants. Where participants have engaged in one or more of these it will be recorded.

Cardiac rehabilitation intervention

Usual care will be optimised as described above. In addition, participants in the intervention arm will be given access to 'Activate Your Heart' (www.activateyourheart.org.uk), which is an online interactive, secure and password-protected website designed for participants to use at home. The programme was co-produced with health care professionals, patients/members of the public and software designers. The programme aims to deliver the

For peer review only

five core components described by the British Association for Cardiovascular Prevention and Rehabilitation (BACPR): health behaviour change and education, lifestyle risk factor management, medical risk factor management, psychosocial health and long-term management [4]. The programme uses the following behaviour change techniques: goal setting, self-monitoring, feedback on behaviour, graded tasks, social reward, providing information about health consequences, and reducing negative emotions. The programme is tailored to individual need, organised in four stages, which can be completed in 8 weeks, but access to the site and its features continues for 12 months. Before beginning the programme, each participant sees a member of cardiac rehabilitation staff who provides training on the website either face-to-face, or remotely by telephone or video consultation. The participant will complete an online registration form, which records their medical history and cardiac risk factors. A paper-based manual is available for participants who are unable or unwilling to use the online rehabilitation system [13]. The website generates a tailored plan from this information, consisting of individualised goals for exercise, diet (eating more fruit and vegetables and reducing salt), emotions (managing stress, anxiety and low mood) and smoking. Adherence with these goals is regularly assessed by a short set of questions and feedback on performance. As the participant progresses through the programme, goals become increasingly difficult. Each participant records details of their daily exercise in an online diary, and regular feedback is given. Smokers are provided with feedback about the amount of money they are saving. The programme also contains written information about the health consequences of heart disease and risk factors (exercise, diet, sexual activity, driving, returning to work, hobbies, holidays, benefits, smoking, anxiety and mood). In addition, the programme reduces negative emotions by providing advice about stress reduction and anxiety management skills. There is access to an online discussion

For peer review only - http://bmjopen.bmj.com/site/about/guidelines.xhtml

forum, and ‘ask the expert’ email facility. Cardiac rehabilitation staff can monitor participants’ progress and respond to questions posted via e-mail. After 12 weeks the cardiac rehabilitation staff member will see the participant again to discuss progress and local exercise opportunities. Cardiac rehabilitation staff will report any safety events that they become aware of.

Outcomes

Participants will complete outcome measures at baseline, 6 and 12 months, administered by practice staff blinded to participant allocation, which are from the standardised outcome measurement for patients with coronary artery disease, agreed by the International Consortium for Health Outcomes Measurement [14] (Tables 1 and 2). The primary outcome will be the UK Version of Seattle Angina Questionnaire (SAQ-UK) Physical Limitation domain [15] assessed at 12 months’ follow-up. Participant completed secondary outcomes will include the remaining two domains of the SAQ-UK [15], MRC dyspnoea scale [16], Hospital Anxiety and Depression Scale (HADS) [17], and General Self-Efficacy Scale [18]. Physical activity will be measured where possible with the ActivPAL accelerometer [19] for 7 days, supplemented by the International Physical Activity Questionnaire [20]. Cardiovascular fitness will be measured with the Incremental Shuttle Walk Test [21]. Economic measures will be EuroQol EQ-5D-5L [22] and Client Service Receipt Inventory (CSRI) [23] as well as intervention costs. Baseline assessments will also include height, weight, blood pressure and serum cholesterol within the last 12 months.

Qualitative interviews will take place with a selection of participants at the end of the 12 week cardiac rehabilitation programme among those receiving the intervention, and

For peer review only - http://bmjopen.bmj.com/site/about/guidelines.xhtml

between months 11 and 12 with the same sample of participants in the intervention group and an additional sample from the control group. These will gather data on trial participation and intervention design (see process evaluation below).

Routinely collected demographic, clinical and recruitment data will include the number of participants who are eligible, willing to be randomised, withdraw after randomisation, complete outcome measurements, reasons for non-completion, age and gender.

Sample size calculation

In order to detect the minimum clinically important difference of 8 points for the SAQ-UK physical limitation domain, assuming a standard deviation of 25 [15], with 90% power at the 5% significance level, 207 participants per group will be required (calculated using nQuery 8). In order to allow for up to 20% loss to follow up, we aim to recruit 259 participants per group (518 in total). This sample size will give >99% power for secondary outcomes.

Recruitment and Randomisation

Screening and Consent

Written informed consent will be sought from potential participants who will be approached by the study team and invited to consider participation, following a search of their health record for eligibility. Where possible, potential participants will be introduced to the study during a face-to-face appointment with clinical staff. Potential participants can also be approached by a member of the local research team by written invitation and followed up by a phone call or text message after a week if there has been no response. Potential participants will be invited to attend a screening appointment. If they are eligible, and

For peer review only - http://bmjopen.bmj.com/site/about/guidelines.xhtml

interested in the trial, the trial team researchers would then recruit participants following the trial's informed consent process. These assessments will be recorded in a screening log, including any reasons for ineligibility. Copies of the participant information sheet and informed consent form can be found in Appendix 2 in the supplementary file.

Randomisation Procedures

Participants will be randomised via a secure (24-hour) web-based randomisation system controlled centrally by the clinical trials unit to receive either usual care or tailored cardiac rehabilitation programme ('Activate Your Heart') plus usual care. Randomisation will have an allocation ratio of 1:1 and use a minimisation programme with a built in random element, stratified by site and gender. Randomisation should occur no more than 2 weeks after confirming eligibility, obtaining informed consent, and completing baseline assessments. Following allocation, participants will be notified of their allocation as soon as possible and then receive their randomised treatment allocation.

Blinding

This is a pragmatic trial comparing the 'Activate Your Heart' cardiac rehabilitation intervention with usual care, so it will not be possible to blind participants or their rehabilitation providers to treatment group allocation. However, collection of outcome measures and data analysis will be performed blind to treatment allocation. We will include a perception of allocation form for the person collecting the outcome measures to complete, to monitor the level of blinding achieved for these researchers.

Statistical Analysis

For peer review only - http://bmjopen.bmj.com/site/about/guidelines.xhtml

Final analysis will take place once all participants have been followed-up for 12 months, and the database has been locked. Analyses will be by 'intention to treat' for the primary and secondary outcomes on all randomised participants, in the group to which they were allocated, and for whom the outcomes of interest have been observed or measured. A full and detailed statistical analysis plan (SAP) will be finalised prior to any comparative analysis of the groups; the main features of the SAP are described briefly here.

For peer review only

Baseline

Demographic and clinical characteristics will be summarised separately using descriptive statistics for each randomised group to allow assessment of whether balance was achieved between randomised groups. This will include gender, ethnicity, socio-economic status and other relevant PROGRESS-Plus characteristics [24]. No statistical testing of differences between groups will be performed.

Analysis of effectiveness

Primary and secondary outcomes at baseline, 6 months' and 12 months' follow-up will be summarised for each treatment group using descriptive statistics at each time point. If normally distributed, the difference between group means (with 95% confidence intervals) will be reported from an analysis of covariance (ANCOVA) adjusted for baseline score and stratification factors.

Missing data and withdrawals

For peer review only - http://bmjopen.bmj.com/site/about/guidelines.xhtml

Predictors of missing data will be investigated using regression models (including age, living arrangements and co-morbidities) and any significant predictors will be considered for inclusion in the models. In addition, given the two assessment points at 6 and 12 months, a sensitivity analysis will be conducted using a joint modelling approach to check whether there is any difference in outcome, using longitudinal outcome rather than the outcome at 6 months or 12 months alone, between the randomised arms, adjusted for dropouts or missing values.

Instrumental variable regression

The causal impact of engagement with the intervention will be assessed in an instrumental variable regression model, using the number of stages completed in the rehabilitation programme, and the number of times that the programme is accessed, as measures of intervention use.

Mediation analyses

The hypothesised mechanism of change for the rehabilitation intervention is that participants' primary outcome (physical limitation) is mediated by self-efficacy and physical activity. If the rehabilitation intervention has a significant effect on the primary outcome ($p<0.05$) in ANCOVA, causal mediation analysis will be used to determine whether these potential mediators predict change in SAQ-UK Physical Limitation domain at 12 months.

Economic analysis

The economic evaluation aims to estimate the cost-effectiveness of the 'Activate Your Heart' online rehabilitation programme plus optimal usual care when compared with optimal usual

For peer review only - http://bmjopen.bmj.com/site/about/guidelines.xhtml

care alone. The rehabilitation programme will be fully costed using unit costs from a health service perspective. Unit costs will be obtained from national sources of reference costs and applied to information received from pilot questionnaires, namely salary band of cardiac rehabilitation therapists, time spent with the participant at the initial and final rehabilitation appointments, costs of travel and the costs of registering with the 'Activate Your Heart' online cardiac rehabilitation programme. Participants' health service activity data will be obtained from the CSRI [23] and unit costs from national sources [25, 26]. The costs of service use and the cost of the intervention will be added together for use in a cost-effectiveness analysis. We will account for the likely skewed nature of cost data in any regression carried out, by using Tweedie regression and/or beta distributions. The EQ-5D-5L scores will be converted into a health utility weight using UK norms. The base-case analysis will be conducted using the Van Hout scoring algorithm [27], with sensitivity analysis conducted using the EQ-5D-5L value set for England [28]. Utility indices will be used to calculate QALYs over the 12 month trial period, using the area under the curve approach, with regression-based adjustment for baseline EQ-5D utility scores [29,30]. The health economic results will initially be presented as disaggregated tables of costs alongside the health consequences. In these tables, we will also describe the costs and consequences of the rehabilitation intervention in the underserved groups recruited, such as women, ethnic minorities and those of low socio-economic status.

Costs and consequences will then be combined in a cost-utility analysis, to estimate the cost per additional QALY gained associated with each of the compared options. Deterministic and probabilistic sensitivity analyses will be undertaken to explore the robustness of the obtained results to sample variability and plausible variations in key assumptions and

For peer review only - http://bmjopen.bmj.com/site/about/guidelines.xhtml

employed analytical methods. Cost-effectiveness acceptability curves will be constructed to show the probability that the intervention is cost-effective at specific thresholds of cost per QALY gained such as NICE threshold range of £20,000 to £30,000 per QALY gained [31]. We will develop a two-stage economic model; a decision tree to reflect the outcomes of the trial (within-trial economic evaluation) and a Markov state transition model to extrapolate the evolution of patient outcome and costs over a lifetime horizon (model-based economic evaluation). Value of information analysis will be conducted after taking into account uncertainty derived from decision-analytic models.

Process Evaluation

The concurrent mixed methods process evaluation will aim to identify and explain the mechanisms and processes that enabled or acted as a barrier to the implementation of the cardiac rehabilitation intervention [32,33]. The process evaluation will help build a picture of how the intervention was carried out in reality and what factors shaped it. By conducting a process evaluation, it will be possible to identify if observed impacts are solely due to the cardiac rehabilitation programme, or if these impacts are a result of external and internal variables closely linked to the environment and the context in which the intervention takes place. More specifically, the process evaluation will examine the recruitment of sites and rehabilitation teams, recruitment and reach in participants (from the recruitment log), intervention delivery to individuals (coverage, intensity), participation, retention, response of individuals to the rehabilitation intervention (data collected by the 'Activate Your Heart' website), unintended consequences (adverse events and inequities), contextual factors and sustained motivation for behaviour change. The website will be examined for the degree of completion of the programme, number of occasions that the website was accessed, and the

For peer review only - http://bmjopen.bmj.com/site/about/guidelines.xhtml

For peer review only

extent of the change in behaviour(s) recorded. There will be a particular focus on underserved groups (women, ethnic minorities, low socio-economic status, other PROGRESS-Plus [24] characteristics and those without internet access or device).

Semi-structured telephone or video interviews of participants (n=30) with purposive sampling to ensure diversity based on age, gender, functional impairment, treatment group, site, ethnicity and mode of delivery. A topic guide will be developed with the assistance of our Public Advisory Group, and will ask about the acceptability of the rehabilitation programme and the barriers and facilitators of uptake and completion, such as family support, health inequities, lack of facilities, lack of transport, etc. However, topic guides will be iterative and informed by earlier interviews and adapted accordingly. There will be a particular focus on the acceptability and barriers to implementation in underserved groups (women, ethnic minority and low socio-economic status). Interviews will take place at the end of the 12 week cardiac rehabilitation programme among those receiving the intervention, and between months 11 and 12 in both groups. We will interview a minimum of 10 cardiac rehabilitation staff at different time-points, to gain insight into the early, medium and longer term delivery of the cardiac rehabilitation programme. All of the interviews will be audio recorded and fully transcribed. Participant contact with the online discussion forum, and the 'ask the expert' email facility will also be examined. We will also examine data collected routinely by the website for the purposes of the national audit for cardiac rehabilitation. Qualitative data will be analysed using a thematic analysis approach [34]. Quantitative data (recruitment logs, use of the website, recorded change in behaviours, national audit data) will be analysed using descriptive statistics to allow for the

For peer review only - http://bmjopen.bmj.com/site/about/guidelines.xhtml
For peer review only

exploration of frequency of responses. All data sets will be synthesised in order to describe the complex nature of the enhanced rehabilitation intervention.

Patient and Public Involvement

There has been patient and public involvement (PPI) at all stages including developing the research question, choosing outcomes relevant to patients, commenting on the burden of the intervention and of trial participation. Two PPI co-investigators are active members of the trial management group, and help to convene a public advisory group which meets throughout the trial to advise on trial procedures, especially patient facing materials, promotional videos in multiple languages and the dissemination plan. Members of the PPI group recorded voice-overs for the promotional video in different languages.

Ethics and Dissemination

NHS research ethics approval was obtained from North of Scotland Research Ethics Committee, reference 21/NS/0116. The current protocol is version 5 (04/05/2023). A Trial Steering Committee is providing overall supervision and an Independent Data Safety and Monitoring Committee is responsible for reviewing and assessing recruitment, interim monitoring of safety and effectiveness, trial conduct and external data.

All safety events will be recorded by researchers when they are made aware of the event by the participant, treating clinician, or therapist. Adverse event reports and serious adverse events (SAEs) not related to the intervention will be entered on to the remote data entry system. Each SAE will be assessed by the relevant PI to determine whether it is related to

For peer review only - http://bmjopen.bmj.com/site/about/guidelines.xhtml

the intervention. A related SAE will be assessed by the CI to determine whether it is expected. If the SAE is related and unexpected (RUSAE) it will be reported to the Research Ethics Committee (REC) and sponsor in an expedited manner.

Reporting of the trial will be consistent with the CONSORT 2010 Statement (patient reported outcomes and non-pharmacological interventions) [35]. We will submit the final report to a peer-reviewed academic journal, according to our publication strategy and authorship policy. Research data will be available for secondary analysis upon reasonable request.

Trial Status

At the time of submission this trial had been open in 20 sites, with 10 more sites in preparation, and had recruited 62 participants. Updated recruitment information will be found on the website http://activate-trial.org.uk

Abbreviations

ANCOVA	Analysis of Covariance
ARC NWC	Applied Research Collaboration North West Coast
BACPR	British Association for Cardiovascular Prevention and Rehabilitation
CI	Chief Investigator
CONSORT	Consolidated Standards of Reporting Trials.
CSRI	Client Service Receipt Inventory

For peer review only - http://bmjopen.bmj.com/site/about/guidelines.xhtml

EQ-5D-5L	EuroQol 5 Dimensions 5 Levels
GP	General Practitioner
HADS	Hospital Anxiety and Depression Scale
IPAQ	International Physical Activity Questionnaire
ISRCTN	International Standard Randomised Controlled Trial Number
IV	Instrumental Variable
LCTC	Liverpool Clinical Trials Centre
MI	Myocardial Infarction
MRC	Medical Research Council
NHS	National Health Service
NICE	National Institute of Health and Clinical Excellence
NIHR	National Institute for Health and Care Research
PCI	Percutaneous Coronary Intervention
PI	Principal Investigator
PPI	Patient and Public Involvement
QALY	Quality Adjusted Life Year
QoL	Quality of Life
RCT	Randomised Controlled Trial
REC	Research Ethics Committee
RUSAE	Related Unexpected Serious Adverse Event
SAE	Serious Adverse Event
SAQ-UK	United Kingdom version of Seattle Angina Questionnaire

For peer review only - http://bmjopen.bmj.com/site/about/guidelines.xhtml

References

1. Eisen A, et al. REACH Registry Investigators. Angina and Future Cardiovascular Events in Stable Patients with Coronary Artery Disease: Insights from the Reduction of Atherothrombosis for Continued Health (REACH) Registry.JAmHeartAssoc 2016 28;5(10).
2. Knuuti J, et al. 2019 ESC Guidelines for the diagnosis and management of chronic coronary syndromes: The Task Force for the diagnosis & management of chronic coronary syndromes of the European Society of Cardiology (ESC).EurHeartJ 2020 41:407–77.
3. National Institute for Health and Clinical Excellence. CG126. Stable angina: management. https://www.nice.org.uk/guidance/cg126/resources/stable-angina-management-pdf-35109453262021 Accessed 15 September 2023.
4. British Association for Cardiovascular Prevention and Rehabilitation (BACPR) Standards and Core Components for Cardiovascular Disease Prevention and Rehabilitation 2023 (4th Edition). https://www.bacpr.org/__data/assets/pdf_file/0021/64236/BACPR-Standards-and-Core-Components-2023.pdf Accessed 15 September 2023.
5. Fihn SD, et al. 2012 ACC/AHA/AATS/PCNA/SCAI/STS Guideline for the Diagnosis and Management of Patients With Stable Ischemic Heart Disease. J Am Coll Cardiol 2012; 60: e44-164.
6. British Heart Foundation. The National Audit of Cardiac Rehabilitation: Quality and Outcomes Report 2019. https://www.bhf.org.uk/informationsupport/publications/statistics/national-audit-of-cardiacrehabilitation-quality-and-outcomes-report-2019. Accessed 03 July 2020.
7. Taylor RS, et al. Exercise-based rehabilitation for heart failure. Cochrane Database of Systematic Reviews 2014, Issue 4. Art. No.: CD003331.
8. Heran BS, et al. Exercise-based cardiac rehabilitation for coronary heart disease. Cochrane Database of Systematic Reviews 2011, Issue 7. Art. No.: CD001800.
9. Long L, et al. Exercise-based cardiac rehabilitation for adults with stable angina. Cochrane Database of Systematic Reviews 2018, Issue 2. Art. No.: CD012786.
10. Devi R, Powell J, Singh S, A Web-Based Program Improves Physical Activity Outcomes in a Primary Care Angina Population: Randomized Controlled Trial J Med Internet Res 2014;16(9):e186 URL: https://www.jmir.org/2014/9/e186, DOI: 10.2196/jmir.3340
11. National Institute for Health and Clinical Excellence. CG181. Cardiovascular disease: risk assessment and reduction, including lipid modification. https://www.nice.org.uk/guidance/cg181/resources/cardiovascular-disease-risk-assessment-and-reduction-including-lipid-modification-pdf-35109807660997 Accessed 15 September 2023.
12. National Institute for Health and Clinical Excellence. CG136. Hypertension in adults: diagnosis and management. https://www.nice.org.uk/guidance/ng136/resources/hypertension-in-adults-diagnosis-and-management-pdf-66141722710213 Accessed 15 September 2023.
13. Warschauer M, Matuchinak T. New technology and digital worlds: Analyzing evidence of equity in access, use, and outcomes. Review of Research in Education 2010; 34: 179-225.
14. McNamara RL, et al. Standardized Outcome Measurement for Patients With Coronary Artery Disease: Consensus From the International Consortium for Health Outcomes Measurement (ICHOM). J Am Heart Assoc 2015; 4: e001767.
15. Garratt A, Hutchinson A, Russell IT. The UK version of the Seattle Angina Questionnaire (SAQ-UK): Reliability, validity and responsiveness. J Clin Epidemiol 2001; 54: 907-15.
16. MRC dyspnoea scale. https://www.ukri.org/councils/mrc/facilities-and-resources/find-an-mrc-facility-or-resource/mrc-dyspnoea-scale/ Accessed 08 April 2023.

For peer review only - http://bmjopen.bmj.com/site/about/guidelines.xhtml

17. Zigmond A, Snaith R. The Hospital Anxiety and Depression Scale. Acta Psychiatr Scand. 983; 67: 1983.
18. Schwarzer R, Jerusalem M. Generalized Self-Efficacy scale, Measures in health psychology: a user's portfolio. Causal and control beliefs. Windsor, UK: NFER-NELSON, 1995.
19. Edwardson CL, Winkler EAH, Bodicoat DH, Yates T, Davies MJ, Dunstan DW, Healy GN. Considerations when using the activPAL monitor in field-based research with adult populations. J Sport Health Sci 2017; 6: 162-178.
20. Craig CL, et al. International physical activity questionnaire: 12-country reliability and validity. Med Sci Sports Exerc 2003; 35: 1381-95.
21. Singh SJ, Morgan MDL, Scott S, Walters D, Hardman AE. Development of a shuttle walking test of disability in patients with chronic airways obstruction. Thorax 1992; 47: 1019-1024.
22. EuroQol Group. EuroQol – a new facility for the measurements of health-related quality of life. Health Policy 1990;16:199-208.
23. Database of Instruments for Resource Use Measurement. Client Service Receipt Inventory (CSRI). http://www.dirum.org/instruments/details/44. Accessed 31 January 2019.
24. O'Neill J, Tabish H, Welch V, Petticrew M, Pottie K, Clarke M, Evans T, Pardo J, Waters E, White H, Tugwell P. Applying an equity lens to interventions: using PROGRESS ensures consideration of socially stratifying factors to illuminate inequities in health. J Clin Epidemiol 2014; 67: 56-64.
25. Department of Health. NHS reference costs 2015 to 2016. https://www.gov.uk/government/publications/nhs-reference-costs-2015-to-2016 Accessed 03 July 2020.
26. Curtis L, Burns A. Unit costs of health and social care 2019. Personal Social Services Research Unit (PSSRU). https://www.pssru.ac.uk/project-pages/unit-costs/unit-costs-2019/ Accessed 03 July 2020.
27. van Hout B, et al. Interim Scoring for the EQ-5D-5L: Mapping the EQ-5D-5L to EQ-5D-3L Value Sets. Value in Health 2012; 15: 708-715.
28. Devlin NJ, Shah KK, Feng Y, Mulhern B, Van Hout B. Valuing health-related quality of life: An EQ-5D-5L value set for England. Health Economics. 2018;27(1):7-22.
29. Glick H, Doshi J, Sonnad S, Polsky D. Economic evaluation in clinical trials. Oxford: Oxford University Press; 2007.
30. Drummond MF, Sculpher MJ, Torrance GW, O'Brien BJ, Stoddart GL. Methods for the economic evaluation of health care programmes. Oxford: Oxford University Press; 2005.
31. National Institute for Health and Care Excellence. Guide to the methods of technology appraisal. http://www.nice.org.uk/article/pmg9/resources/non-guidance-guide-to-themethods-of-technology-appraisal-2013-pdf. 2013. Accessed on 03 July 2020.
32. Oakley, A., Strange V., Bonell C., Allen E, Stephenson J. Process evaluation in randomised controlled trials of complex interventions. Health Services Research 2006; 332: 413-416.
33. Moore GF, Audrey S, Barker M, Bond L, Bonell C, Hardeman W, Moore L, O'Cathain A, Tinati T, Wight D, Baird J. Process evaluation of complex interventions: Medical Research Council guidance. BMJ 2015; 350: h1258.
34. Braun V, Clarke V. Using thematic analysis in psychology. Qualitative Research in Psychology 2006; 3: 77-101.
35. Schulz KF, Altman DG , Moher D , et al. CONSORT 2010 statement: updated guidelines for reporting parallel group randomised trials. BMC Med 2010;8:18

For peer review only - http://bmjopen.bmj.com/site/about/guidelines.xhtml

Author Statement

NHW is the chief investigator and grant holder, and SS is the joint lead investigator and they are responsible for study design, conduct and analysis, have overall responsibility for the study and act as guarantors. SH is the trial co-ordinator and BH the senior trial co-ordinator overseeing day-to-day conduct, and providing methodological input. SDod will write the statistical analysis plan. BC will write the health economic analysis plan. DL and PR will write the process evaluation analysis plan with NHW. EM and TJC are Patient and Public Involvement co-investigators. GL, MF, TJ and KJ are co-investigators responsible for study design, methodological oversight and provide cardiology (GL, MF), exercise science (TJ) and primary care (NHW, KJ) expertise. All authors were involved in drafting, revising and approving this manuscript.

Acknowledgements

The authors would like to thank all of the principal investigators, research nurses, hospital staff, cardiac rehabilitation teams, leisure centre and exercise referral scheme staff: Dr Timothy Smith (Pendle Medical Partnership,) Lisa Robson (Pendle View Medical Centre,) Dr Tanveer Ahmed Dr Eve Frost (Shifa Surgery,) Vishal Mashru (Billesdon Surgery, Croft Medical Centre, South Leicestershire Medical Group,) Dr Selena Harris, Maisie Wrightson (Clarence House, Dr Jenny Liddell (Corwen Medical Centre,) Dr Joanna Le Noury (Plas Menai Surgery), Dr Helen Alefounder, Dr Jessica Deacon, Lucie Parrie, Chantal Stewart (West End Medical Centre,) Dr Nadja Van Ginneken, Theresa Nuttall, Jessica Keane (Brownlow Group,) Dr Stephen Forster (Heswall and Pensby Group Practice,) Dr David Lewis, Ann Kent, Carol Burgess, Carrie Geldhart (Vauxhall Primary Care Health Centre), Dr Helen Steiner, Stella Oldham (The Village Practice,) Dr Rebecca Sinfield, Hannah Mcgowan (St George's Medical

For peer review only - http://bmjopen.bmj.com/site/about/guidelines.xhtml

For peer review only

Centre,) Dr Gareth Powell, Michelle Morgan, Shirley Salter (Llan Healthcare,) Dr Michael Fisher, Laura Pennington, Anthony Burns, Daniel Evans, Ahmed Elamin (Liverpool University Hospitals NHS Foundation Trust), Professor Sally Singh, George Hudson, Donna Alexander (Leicester Hospitals NHS Trust.)

Special thanks to the LCTC head of data management and monitoring Clare Jackson, LCTC information systems team Sharon Keane, Janet Harrison, Keith Kennedy, Tim Charter, trial co-ordinator assistant Eleanor MacDonald and quality assurance managers Katie Neville and Claire Johnson. Also the North West Coast Clinical research Network.

We very much appreciate the work of the Trial Steering Committee Clare Wilkinson (chair), Robert Copeland (Independent Member) Rod Taylor (Independent Member), Katie Pike (Independent Statistician), Tracy Kitto (Independent Member), Keith Wilson (PPI Representative); Independent Data and Safety Monitoring Committee Umesh Chauhan (chair), Catriona Keerie (Independent Statistician), Ahmet Fuat (Independent Member), Adrian Taylor (Independent Member); PPI team Terrence Comerford, Ray Martin, Erica Morgan, Hameed Khan, Naheed Tahir, Robert Smart and Lynn Hedgecoe.

For peer review only - http://bmjopen.bmj.com/site/about/guidelines.xhtml

Funding

This work was funded by the National Institute for Health Research (NIHR) Health Technology Assessment Programme, grant number 131015. It was supported by the NIHR Applied Research Collaboration North West Coast, NIHR Clinical Research Network North West Coat and NIHR Leicester Biomedical Research Centre. SS and GL are NIHR Senior investigators. KJ is part-funded by the NIHR Applied Research Collaboration West Midlands. The views and opinions expressed therein are those of the authors and do not necessarily reflect those of the HTA, NIHR, NHS or the Department of Health.

Trial Sponsor

University of Liverpool: sponsor’s reference: UoL001635. Contact: Karen Wilding, Senior Clinical Research Governance Manager, University of Liverpool. The University has appropriate clinical trials and professional indemnity insurance.

Conflicts of Interest

NHW reports additional grants from NIHR HTA outside the submitted work and deputy chair of the NIHR HTA programme funding committee (commissioned research). DAL reports additional grants from the NIHR outside the submitted work and investigator-initiated educational grants from BMS and Pfizer. DAL has been a speaker for Bayer, Boehringer Ingelheim, and BMS/Pfizer and has consulted for BMS, and Boehringer Ingelheim; all outside the submitted work. GHYL has been a consultant and speaker for BMS/Pfizer, Medtronic, Boehringer Ingelheim and Daiichi-Sankyo. No fees were directly received personally. BC works part-time for Welsh Government outside of the submitted work. KJ was sub-

For peer review only

committee chair of the NIHR Programme Grants for Applied Research until 2023. BH reports additional grants from the NIHR outside the submitted work and investigator-initiated grant from Novartis

Data Statement

Technical appendix, statistical code, and dataset will be made available.

For peer review only

For peer review only - http://bmjopen.bmj.com/site/about/guidelines.xhtml

Table 1 ACTIVATE Outcome measures

**Patient Completed Measure - Primary**	**Description**	**Range**
UK version of Seattle Angina Questionnaire (SAQ-UK) Physical Limitation domain [15]	Measures how daily activities are limited by angina over the past 4 weeks. Items are weighted equally, summed and transformed to a 0-100 score. Higher score indicates better functioning.	(0-100)
**Patient Completed Measures - Secondary**		
UK version of Seattle Angina Questionnaire (SAQ-UK) Angina Frequency and Perception and Treatment Satisfaction domains [15]	Remaining two domains of SAQ-UK. Items are weighted equally, summed and transformed to a 0-100 score. Higher scores represent higher levels of health or satisfaction with treatment.	(0-100)
MRC dyspnoea scale [16]	Five item questionnaire that assesses the degree of shortness of breath with common activities. One point is assigned to each activity associated with dyspnoea. Higher scores indicates more limitation due to dyspnoea.	(0-5)
Hospital Anxiety and Depression Scale (HADS) [17]	Measures anxiety (7 items) and depression (7 items) in patients with physical health problems. Two sub-scales with higher scores indicating greater anxiety or depression.	(0-21)
General Self-Efficacy scale [18]	Measures optimistic self-belief to cope with a variety of difficult demands in life. Higher score indicates greater self-efficacy.	(10-40)
International Physical Activity Questionnaire (IPAQ) [20]	This questionnaire supplements the accelerometer data by providing self-reported duration (minutes) and frequency (days) of physical activity in the previous 7 days in the domains: job-related, transportation, housework, recreation, and sitting.	Minutes of light, moderate and vigorous physical activity

For peer review only - http://bmjopen.bmj.com/site/about/guidelines.xhtml

**Patient Completed Economic Measures**		
EuroQol EQ-5D-5L [22]	Health utility index with five dimensions (mobility, self-care, usual activities, pain/discomfort, anxiety/depression) and five levels to give health states converted to a utility weight. Also Visual Analogue Score (VAS) for health state today	Health utility weight from 0 (death) to 1.0 (perfect health) also with negative values VAS (0-100)
Client Service Receipt Inventory (CSRI) [23]	Collects information about health service use, including medication, smoking cessation, diet management, weight control programmes and exercise referral schemes.	According to activity
**Objective measures of physical activity and function**		
Physical activity measured with the ActivPAL accelerometer [19]	The ActivPAL device is a small, slim monitor worn on the thigh. It provides accelerometer-derived information about thigh position and acceleration to determine body posture (sitting, lying or upright), stepping, and stepping speed (cadence), from which energy expenditure can be inferred. It will be worn for 7 days to measure step count, energy expenditure, duration of sedentary, light, moderate and vigorous physical activity. Participants will be asked to keep a wear diary documenting working hours, sleep, removal reasons and any other comments.	Step count, energy expenditure, duration of sedentary, light, moderate and vigorous physical activity
Incremental Shuttle Walk Test [21]	Progressive walking test that requires the participant to walk up and down a 10 m course, at a speed dictated by an audio signal. The time between signals is reduced every minute and there are 12 levels. The test ends when the participant either (a) is too breathless to maintain the required speed or (b) fails to complete a shuttle in the time allowed or (c) reaches 85% of the predicted maximal heart rate.	Metres walked

For peer review only - http://bmjopen.bmj.com/site/about/guidelines.xhtml

Table 2 ACTIVATE protocol schedule of forms and procedures

Procedures	Screening	Baseline / Randomisation	7 days post randomisation*	Trial intervention**	26 weeks post randomisation	27 weeks post randomisation*	52weeks post randomisation	53 weeks post randomisation*
**Eligibility screening and consent**								
Assessment of eligibility	X							
Informed consent	X							
Confirm consent		X		X	X		X	
Randomisation		X						
Vital signs, height, weight, serum cholesterol		X						
**Outcome measurement**								
Seattle Angina Questionnaire		X			X		X	
MRC dyspnoea scale		X			X		X	
Hospital Anxiety and Depression Scale		X			X		X	
General Self-Efficacy scale		X			X		X	
EuroQol EQ-5D-5L		X			X		X	
Client Service Receipt Inventory		X			X		X	
Physical activity measured with ActivPal accelerometer		X	X*		X	X*	X	X*
International Physical Activity Questionnaire			X			X		X
Incremental shuttle walk test		X			X		X	
**Safety Event Reporting**								
Monitoring of Adverse Events			X	X	X	X	X	X
Monitoring of Serious Adverse Events			X	X	X	X	X	X

* Removal of ActivPal after 7 days

** If randomised to intervention arm, participants will be invited to meet with member of cardiac rehabilitation staff who will train the participant on how to use the intervention

For peer review only - http://bmjopen.bmj.com/site/about/guidelines.xhtml

Figure Legend

Figure 1 **Participant Flowchart for ACTIVATE**

For peer review only

For peer review only - http://bmjopen.bmj.com/site/about/guidelines.xhtml

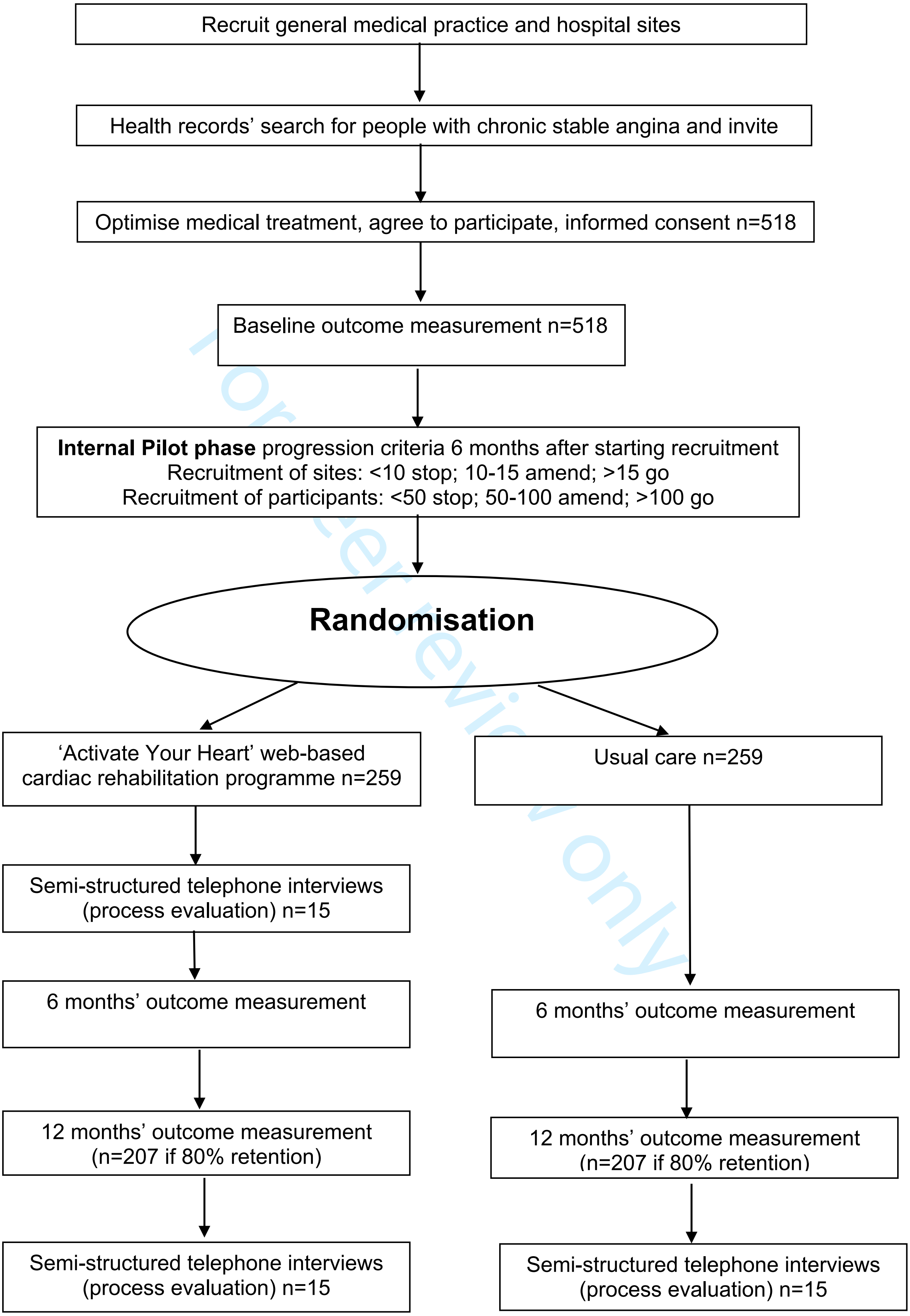

For peer review only

Appendix 1 ACTIVATE Trial Registration Data

**Data category**	**Information**
Registry and trial identification no.	ISRCTN10054455
Date of registration	16/09/2021
Funder	NIHR Evaluation, Trials and Studies Co-ordinating Centre (NETSCC); Grant code 131015
Sponsor	University of Liverpool
Contact for public enquiries	email: activate.trial@liverpool.ac.uk
Scientific title	Cardiac rehabilitation for people with chronic stable angina
Acronym	Angina Controlled Trial Investigating the Value of the 'Activate Your Heart' E-intervention (ACTIVATE)
Countries of recruitment	United Kingdom
Health condition	Angina pectoris
Intervention	Intervention comparator: Web-based cardiac rehabilitation ('Activate Your Heart')
	Control comparator: Usual care
Inclusion criteria	Adult patients (age >18 years)
	Chronic stable angina with at least 2 out of 3 of the following features:<br>i) Constricting central chest pain<br>ii) Precipitated by exertion or emotional stress<br>iii) Relieved by rest or glyceryl trinitrate spray
	Evidence of myocardial ischaemia from either a past medical history of acute coronary syndrome, myocardial infarction (MI) or revascularisation procedure at least 12 months in the past; or from imaging studies such as invasive coronary angiography, computerised tomography angiography or myocardial perfusion testing.
	Revascularisation procedures not planned and treated with medical treatments only, including people with previous MI, or previous revascularisation procedure who may have attended cardiac rehabilitation in the past.
Exclusion criteria	History of myocardial infarction (MI) within the last 12 months.
	History of revascularisation procedure within the last 12 months, or planned at the time of study recruitment.
	Participation in a cardiac rehabilitation programme within the last 12 months, or planned at time of study recruitment.

For peer review only

	Significant co-morbidities (as deemed by person confirming eligibility) that would limit participation in the exercise-based rehabilitation programme.
	Refractory angina on maximal medical therapy.
Study design	Interventional
	Randomised controlled trial
	Treatment, education or self-management, psychological and behavioural, complex intervention, cardiac rehabilitation
Recruitment start date	01/12/2021
Target sample size	518
Primary outcome	UK version of Seattle Angina Questionnaire (SAQ-UK), physical limitations domain
Secondary outcomes	SAQ-UK angina frequency and perception and treatment satisfaction domains, MRC dyspnoea scale, Hospital Anxiety and Depression Scale, General Self-Efficacy Scale. Physical activity measured with the ActivPAL accelerometer for 7 days, supplemented by the International Physical Activity Questionnaire. Cardiovascular fitness measured with the Incremental Shuttle Walk Test. Economic measures EuroQol EQ-5D-5L and Client Service Receipt Inventory.

For peer review only

<Trust/Site address 1>
<Trust/Site address 2>
<Trust/Site address 3>
<postcode>
Tel: <telephone number>

Participant Information Sheet for ACTIVATE

- You have been invited to take part in a research study. Before you decide, it is important that you understand why the research is being done and what it will involve.
- Please take time to read the following information carefully. **Part 1** tells you the purpose of the study and what will happen to you if you take part. **Part 2** gives you more detailed information about the conduct of the study.
- You can ask a member of your clinical team if there is anything that is not clear, or if you would like more information.
- If you wish you can discuss it with friends, relatives or get independent advice from your local Patient Advice and Liaison Service (PALS) or equivalent.
- **Taking part is voluntary**. You do not need to take part and you do not need to give a reason.
- ACTIVATE is a study to look at cardiac rehabilitation for people with long-term stable angina (chest pain that happens with exertion) to see if a cardiac rehabilitation programme is effective.

How to contact the study team:

If you have any questions about this study please talk to your research team:

- **Principal investigator name and Tel number**
- **Research Nurse name and Tel Number**

Contents:

Part 1

- Why are we doing the ACTIVATE study?
- Do I have to take part?
- What will happen to me if I take part?
- What will I have to do if I take part?
- How will I know which treatment I'm going to have?
- What are the benefits and risks of taking part?
- What are the alternatives for treatment?
- What happens if I change my mind?
- What if new information becomes available?
- What happens when the study stops?
- What if there is a problem?
- Will my taking part in the study be kept confidential?

Part 2

- Who is running the study?
- How will my information be collected and handled?
- What are my choices about how my information is used?
- Information sharing for other research
- Where can I find out more about how my information is used?
- What if there is a problem?

For peer review only - http://bmjopen.bmj.com/site/about/guidelines.xhtml

PART 1: Purpose of the study and what will happen if you take part

Why are we doing the ACTIVATE study?

The aim of the study is to improve the health of people with long-term angina which limits daily activities. We want to see whether a rehabilitation programme combined with usual heart care is better than usual heart care alone.

Angina is chest pain brought on by physical activity or stress and is caused by narrowed blood vessels restricting the flow of blood to the heart muscle. It can severely limit day to day activities. Cardiac rehabilitation programmes promote the physical and mental health of people with heart disease by increasing their understanding and improving their physical fitness levels. Cardiac rehabilitation is normally recommended after heart attacks or heart surgery, but not for people with stable angina who do not require an operation. Although cardiac rehabilitation might also benefit people with stable angina, more evidence is needed before it can be used in standard care.

Participants will be split into two groups randomly. Group A will receive usual care from your healthcare provider plus a cardiac rehabilitation programme called 'Activate Your Heart'. Group B will receive usual care from your healthcare provider. 'Activate Your Heart' is an interactive website for you to use at home. The programme was developed by a team of health care professionals, people with angina and software designers. The programme helps you to set goals, monitor progress and gives you feedback on how you are doing. It encourages you to gradually increase your activity level. A paper manual will be available for participants who are unable to use the online version or for those who feel they would prefer a paper version. We would also like participants to complete some questionnaires whilst taking part in the study and also wear a small device called an ActivPAL device which looks out how active you are.

We want to recruit 518 participants aged 18+ years with long-term stable angina. Each participant will be asked to take part in the study for 12 months.

Do I have to take part?

No, **taking part is voluntary**. It is up to you to decide whether or not you want to take part.

If you decide not to take part then you will still receive the usual treatment your healthcare provider offers.

If you decide to take part you can also choose to stop at any time without giving a reason. The decision you make on whether to take part or not will not affect the standard of care you receive now or in the future.

What will happen to me if I take part?

A member of the clinical team can talk to you in more detail about this study and you will be able to ask any questions that you have. If you have had all of your questions answered and are happy to take part then you will be asked to sign a consent form to confirm you want to take part.

Whichever group you are allocated, we will ask you to attend the visits shown in the flowchart on page 3.

What will I have to do if I take part?

Once you have signed the consent form, we will check and confirm that this study is suitable for you and you will be asked to follow the study plan (see study timeline on page 3). You will have to:

- Attend your GP surgery or hospital for follow-up visits over a 12-month period. It may be possible to do these over the telephone or video call.
- At each follow-up visit we will ask you to complete a set of questionnaires.
- If you are randomly allocated to receive the 'Activate Your Heart' intervention you will be invited to meet with someone who will explain how the system works. A member of the research team will contact you to arrange a suitable date to meet and give you instructions on where to meet.

For peer review only

For peer review only - http://bmjopen.bmj.com/site/about/guidelines.xhtml

Study Timeline

Week 0

Baseline Assessment (Both Groups) (90 minutes)
You will be asked to sign a consent form. Your healthcare provider will then do some routine assessments, ask you to complete some questionnaires and fit an ActivPAL

Week 1

7 days later and Randomisation (Both Groups) (30 minutes)
The ActivPAL will be removed and you will be asked to complete a questionnaire. You will be assigned to treatment group A or treatment group B by chance.

Week 2-3

Intervention Training (Group A only) (60 minutes)
You will be asked to attend training with our cardiac specialist on the 'Activate Your Heart' online programme.

Week 27

Follow-up Visit 1 (Both Groups) (60 minutes)
Your healthcare provider will do some investigations, ask you to complete some questionnaires and fit an ActivPAL

Week 28

7 days after Follow-up Visit 1 (Both Groups) (30 minutes)
The ActivPAL will be removed and you will be asked to complete a questionnaire.

Week 53

Follow-up Visit 2 (Both Groups) (60 minutes)
Your healthcare provider will do some investigations, ask you to complete some questionnaires and fit an ActivPAL

Week 54

7 days after Follow-up Visit 2 (Both Groups) (30 minutes)
The ActivPAL will be removed and you will be asked to complete a questionnaire.

**Procedure**	**Description**	**Research Treatment or Standard of care**
Activate your heart	This is a secure, interactive website designed for you to use at home. The programme is tailored to individual need and is in four stages, which can be completed in 8 weeks, but your access to the site and its features continue for 12 months. A paper version is also available for any participant who does not want to access the website.	**Research Treatment**
Questionnaires	We will ask you to complete a set of questionnaires about your health, and use of health services, when you start taking part, again after 6 months and then a final set of questionnaires at 12 months.	**Research Treatment**
Physical activity measured with the ActivPAL accelerometer	The ActivPAL device is a small, slim monitor worn on the thigh. We will ask you to wear it for 7 days when you first take part, for another 7 days after 6 months and then a final 7 days after 12 months. The device will look at how physically active you are. We will also ask you to keep a diary documenting working hours, sleep, reasons for not wearing it, and any other comments	**Research Treatment**

For peer review only - http://bmjopen.bmj.com/site/about/guidelines.xhtml

Procedure	Description	Research Treatment or Standard of care
Incremental Shuttle Walk Test	We will ask you to complete this test when you start taking part, again after 6 months and then finally at 12 months. For this walking test we will ask you to walk back and forth along a 10-metre track at a comfortable pace, every minute the pace will increase. The distance you can walk will give us an idea of your exercise capacity.	Research Treatment
Height, weight, blood pressure and Serum Cholesterol	These are standard tests that you will have done when you visit the Doctor, we would like to collect this information for our study.	Standard of Care

Optional telephone interviews

We will interview a selection of participants over the telephone to ask about your experience of being in the study. These will last up to 1 hour and will be recorded. We will offer a £30 shopping voucher for your time.

How will I know which treatment I get?

In the ACTIVATE study participants will be split into two treatment groups. Your healthcare provider will let you know what group you are in. We use a computer programme that puts participants into groups by chance:

- One group will receive standard of care treatment plus have access to the online 'Activate Your Heart' rehabilitation programme.
- The other group will receive standard of care treatment only.

Are there any risks of taking part?

Experience from using the 'Activate Your Heart' programme for cardiac rehabilitation has shown that it is very safe. There may be a small risk of injury or provoking an episode of angina when exercising, but to prevent this, the physical activity goals are carefully set. You can contact the cardiac rehabilitation therapists for advice and support or by joining an online scheduled weekly chat room.

What happens if I change my mind?

If at any point you decide to stop taking part in the study you will still receive your usual treatment from your healthcare provider. If you do decide to stop taking part we will ask you if you would like to: continue to complete follow up visits for the study or stop taking part with no more study visits.

What happens when the study stops?

When you finish taking part in the study your healthcare provider will discuss what treatment is best for you next.

The results of the study will be presented at conferences and published in medical journals. Confidentiality will be ensured at all times and you will not be identified in any publication.

Any information resulting directly or indirectly from this research, as well as any patents developed directly or indirectly as a result of this research may be used for commercial purposes. You have no right to this property or to any share of the profits that may be earned directly or indirectly as a result of this research. However, in signing this form for this research, you do not give up any rights that you would otherwise have as a participant in research.

What if there is a problem?

We don't expect there to be any problems the study has been carefully designed to ensure your safety is the main priority. Any complaint about the way you have been dealt with during the study or any possible harm you might suffer will be addressed. See Part 2 below.

Will my taking part be kept confidential?

For peer review only

For peer review only - http://bmjopen.bmj.com/site/about/guidelines.xhtml

Yes. All the confidential information about your participation in this study will be kept confidential. Detailed information on this is given in Part 2.

For peer review only

For peer review only - http://bmjopen.bmj.com/site/about/guidelines.xhtml

PART 2: Detailed Information about the conduct of the study

Who is running the study?

The University of Liverpool is the Sponsor of this study and is responsible for managing it. They have asked that the day to day running of the study is carried out by a team based at the Liverpool Clinical Trials Centre (LCTC, part of the University of Liverpool) and qualitative and health economic researchers (part of the University of Liverpool).

The study has been reviewed by the Health Research Authority and the North of Scotland Research Ethics Committee (2).

This study is funded by the National Institute for Health Research's Health Technology Assessment Programme (ref: NIHR: 131015).

Your healthcare provider will not receive any payment for including you in this study.

How will my information be collected and handled?

The University of Liverpool is the Data Controller for this study and will need to use information from you and your medical records for this research project.

This information will include your initials, NHS number and name. If you are in the rehabilitation group (Group A of the study), your contact details will be sent to the cardiac rehabilitation team, so that they can contact you to arrange an appointment to explain how to use the 'Activate Your Heart' programme. Your contact details will also be shared with qualitative researchers at The University of Liverpool so they can contact you for telephone/video-call interviews. People will use this information to do the research or to check your records to make sure that the research is being done properly.

Individuals from The University of Liverpool, the LCTC and regulatory organisations may look at your medical and research records to check the accuracy of the research study.

People who do not need to know who you are will not be able to see your name or contact details. Your data will have a code number instead.

Data will be entered into a secure database from your healthcare team and staff members at the LCTC will be able to see this data. The LCTC will share some data with qualitative researchers and health economics researchers. The researchers doing the interviews will contact 30 participants about 3 months after the start of the study and again after 12 months to ask questions about how they felt being part of the study. These interviews can take place on the telephone or video call.

Health economic researchers at The University of Liverpool want to use your health economic data (number of visits to hospital, medication taken etc.) to help calculate the overall costs of care. We will ask you to complete a questionnaire called Client Service Receipt Inventory (CSRI). This is a tool used to collect information on the whole range of services and supports study participants may use. We will share the information you provide in this questionnaire with health economic researchers at The University of Liverpool.

We will keep all information about you safe and secure.

Once we have finished the study, we will keep the data for 10 years, so we can check the results. We will write our reports in a way so that no-one can work out that you took part in the study.

You can find out more about how we use your information at www.activate-trial.org.uk

What are my choices about how my information is used?

You can stop being part of the study at any time, without giving a reason, but we will keep information about you that we already have.

For peer review only

For peer review only - http://bmjopen.bmj.com/site/about/guidelines.xhtml

If you choose to stop taking part in the study, we would like to continue collecting information about your health from central NHS records, your hospital and your GP. If you do not want this to happen, please tell your healthcare provider

If you lose capacity during the study we would still plan to use the information collected from you.

We need to manage your records in specific ways for the research to be reliable. This means that we will not be able to let you see or change the data we hold about you.

Information sharing for other research

When you agree to take part in a research study, the information about your health and care may be beneficial to researchers running other research studies in this organisation and in other organisations. These organisations may be universities, NHS organisations or companies involved in health and care research in this country or abroad. Your information will only be used by organisations and researchers to conduct research in accordance with the UK Policy Framework for Health and Social Care Research, or equivalent standards.

If you agree to take part in this study, you will have the option to take part in future research using your anonymous data saved from this study.

Where can I find out more about how my information is used?

You can find out more about how we use your information:

- at the study website: www.activate-trial.org.uk
- at www.hrs.nhs.uk/information-about-patients
- in the Health Research Authority leaflet available from www.hra.nhs.uk/patientdataandresearch
- by contacting the University of Liverpool Data Protection Officer on LegalServices@liverpool.ac.uk.
- by asking one of the research team

What if there is a problem?

If you have a concern about any aspect of this study, you should ask to speak with one of your research team who will do their best to answer your questions.

If you remain unhappy and wish to complain formally, you can do this by contacting local NHS Patient Advice and Liaison Service (PALS) or equivalent. Members of your local hospital team or GP should be able to provide this information to you.

Every care will be taken in the course of this study. However, in the unlikely event that you are harmed by taking part in this research project, compensation from the study Sponsor (The University of Liverpool) may be available, but you may have to pay your related legal costs. The place where you receive your treatment, has a duty of care to you whether or not you agree to participate in the study. The study Sponsor accepts no liability for negligence on the part of your GP practice or healthcare provider. However, if you are harmed, and this is due to someone's negligence at the GP practice or healthcare provider, then you may have grounds for a legal action for compensation against the NHS organisation where you are being treated but you may have to pay for your legal costs. The normal National Health Service complaints procedures should be available to you.

FUNDED BY NIHR | National Institute for Health Research

Thank you for taking the time to read and consider this information sheet. Should you decide to take part in the study, you will be given a copy of the information sheet and a signed consent form to keep.

For peer review only

For peer review only - http://bmjopen.bmj.com/site/about/guidelines.xhtml

ACTIVATE

<Trust/Site address 1>
<Trust/Site address 2>
<Trust/Site address 3>
<postcode>
Tel: <telephone number>

Participant Consent Form

FOR SITE USE ONLY:

Site Name:	
Participant Study Number	
Participant Initials:	Participant DOB: / /

To be completed by the participant:

Once you have read and understood each statement please enter your initials in each box.	Initial
1. I have read and understood the information sheet for this study. I have had the opportunity to ask questions and have had these answered satisfactorily.	
2. I understand that participation is voluntary and that I am free to withdraw from the study at any time, without giving a reason, and without my care or legal rights being affected. However, the study team may need to collect some limited information for safety reasons.	
3. I give permission for a copy of this fully completed consent form to be sent to the LCTC (where it will be kept in a secure location) to allow confirmation that my consent was given.	
4. I understand that relevant sections of my medical notes and any data collected during the study may be looked at by authorised individuals from the central study team and representatives of the Sponsor, regulatory authorities and my healthcare provider. I give permission for these individuals to have access to my records and data.	
5. I agree to my GP being informed of my participation in the study.	
6. I agree for my data collected on the Client Service Receipt Inventory (CSRI) form to be shared with researchers at The University of Liverpool for the purpose of health economic research.	
7. I understand that my data will be kept by the University of Liverpool and at my healthcare provider in a confidential manner for 10 years from the end of the study.	
8. I agree to take part in the above study.	
The statements below are optional (you can still take part in the study even if you do not wish to agree to these):	
9. I agree to allow information or results arising from this study to be used in future healthcare and/or medical research providing my confidentiality is maintained.	
10. I agree to being contacted by a study researcher to conduct a qualitative interview and for the interview to be recorded. (if you agree to this statement provide your details below):	
11. I agree for any of my feedback from the qualitative interviews to be used as quotes in future publications, presentations and on the study website and newsletters	

Telephone number:	
Email address	

Please continue to the next page.

For peer review only

For peer review only - http://bmjopen.bmj.com/site/about/guidelines.xhtml

ACTIVATE

<Trust/Site address 1>
<Trust/Site address 2>
<Trust/Site address 3>
<postcode>
Tel: <telephone number>

Participant Consent Form

FOR SITE USE ONLY:

Site Name:			
Participant Study Number			
Participant Initials:		Participant DOB:	/ /

To be completed by the participant:

Your full name (please print):			
Your signature:		Date:	

To be completed by the Researcher (after participant has completed the form):

Researcher full name (please print):			
Researcher signature:		Date:	

Please file the original wet-ink copy in the ACTIVATE Investigator Site File, and make three copies: one for the participant, one for the medical notes and one to be sent to the LCTC.

For peer review only

For peer review only - http://bmjopen.bmj.com/site/about/guidelines.xhtml

ACTIVATE SPIRIT Checklist

SPIRIT 2013 Checklist: Recommended items to address in a clinical trial protocol and related documents*

Section/item	Item No	Description	Addressed on page number
**Administrative information**			
Title	1	Descriptive title identifying the study design, population, interventions, and, if applicable, trial acronym	1
Trial registration	2a	Trial identifier and registry name. If not yet registered, name of intended registry	1,4
	2b	All items from the World Health Organization Trial Registration Data Set	Appendix 1
Protocol version	3	Date and version identifier	20
Funding	4	Sources and types of financial, material, and other support	26
Roles and responsibilities	5a	Names, affiliations, and roles of protocol contributors	1,2,25
	5b	Name and contact information for the trial sponsor	26
	5c	Role of study sponsor and funders, if any, in study design; collection, management, analysis, and interpretation of data; writing of the report; and the decision to submit the report for publication, including whether they will have ultimate authority over any of these activities	26
	5d	Composition, roles, and responsibilities of the coordinating centre, steering committee, endpoint adjudication committee, data management team, and other individuals or groups overseeing the trial, if applicable (see Item 21a for data monitoring committee)	25

For peer review only

**Introduction**			
Background and rationale	6a	Description of research question and justification for undertaking the trial, including summary of relevant studies (published and unpublished) examining benefits and harms for each intervention	5,6
	6b	Explanation for choice of comparators	5,6
Objectives	7	Specific objectives or hypotheses	7
Trial design	8	Description of trial design including type of trial (eg, parallel group, crossover, factorial, single group), allocation ratio, and framework (eg, superiority, equivalence, noninferiority, exploratory)	7,8
**Methods: Participants, interventions, and outcomes**			
Study setting	9	Description of study settings (eg, community clinic, academic hospital) and list of countries where data will be collected. Reference to where list of study sites can be obtained	8
Eligibility criteria	10	Inclusion and exclusion criteria for participants. If applicable, eligibility criteria for study centres and individuals who will perform the interventions (eg, surgeons, psychotherapists)	8,9
Interventions	11a	Interventions for each group with sufficient detail to allow replication, including how and when they will be administered	9-11
	11b	Criteria for discontinuing or modifying allocated interventions for a given trial participant (eg, drug dose change in response to harms, participant request, or improving/worsening disease)	9-11
	11c	Strategies to improve adherence to intervention protocols, and any procedures for monitoring adherence (eg, drug tablet return, laboratory tests)	10-11
	11d	Relevant concomitant care and interventions that are permitted or prohibited during the trial	9-10

Outcomes	12	Primary, secondary, and other outcomes, including the specific measurement variable (eg, systolic blood pressure), analysis metric (eg, change from baseline, final value, time to event), method of aggregation (eg, median, proportion), and time point for each outcome. Explanation of the clinical relevance of chosen efficacy and harm outcomes is strongly recommended	12-13, 27-28
Participant timeline	13	Time schedule of enrolment, interventions (including any run-ins and washouts), assessments, and visits for participants. A schematic diagram is highly recommended (see Figure)	29-30
Sample size	14	Estimated number of participants needed to achieve study objectives and how it was determined, including clinical and statistical assumptions supporting any sample size calculations	13
Recruitment	15	Strategies for achieving adequate participant enrolment to reach target sample size	13

Methods: Assignment of interventions (for controlled trials)

Allocation:

Sequence generation	16a	Method of generating the allocation sequence (eg, computer-generated random numbers), and list of any factors for stratification. To reduce predictability of a random sequence, details of any planned restriction (eg, blocking) should be provided in a separate document that is unavailable to those who enrol participants or assign interventions	14
Allocation concealment mechanism	16b	Mechanism of implementing the allocation sequence (eg, central telephone; sequentially numbered, opaque, sealed envelopes), describing any steps to conceal the sequence until interventions are assigned	14
Implementation	16c	Who will generate the allocation sequence, who will enrol participants, and who will assign participants to interventions	14
Blinding (masking)	17a	Who will be blinded after assignment to interventions (eg, trial participants, care providers, outcome assessors, data analysts), and how	14

	17b	If blinded, circumstances under which unblinding is permissible, and procedure for revealing a participant's allocated intervention during the trial	14
**Methods: Data collection, management, and analysis**			
Data collection methods	18a	Plans for assessment and collection of outcome, baseline, and other trial data, including any related processes to promote data quality (eg, duplicate measurements, training of assessors) and a description of study instruments (eg, questionnaires, laboratory tests) along with their reliability and validity, if known. Reference to where data collection forms can be found, if not in the protocol	12
	18b	Plans to promote participant retention and complete follow-up, including list of any outcome data to be collected for participants who discontinue or deviate from intervention protocols	12
Data management	19	Plans for data entry, coding, security, and storage, including any related processes to promote data quality (eg, double data entry; range checks for data values). Reference to where details of data management procedures can be found, if not in the protocol	12-13
Statistical methods	20a	Statistical methods for analysing primary and secondary outcomes. Reference to where other details of the statistical analysis plan can be found, if not in the protocol	15-16
	20b	Methods for any additional analyses (eg, subgroup and adjusted analyses)	16-20
	20c	Definition of analysis population relating to protocol non-adherence (eg, as randomised analysis), and any statistical methods to handle missing data (eg, multiple imputation)	16

Methods: Monitoring

Data monitoring	21a	Composition of data monitoring committee (DMC); summary of its role and reporting structure; statement of whether it is independent from the sponsor and competing interests; and reference to where further details about its charter can be found, if not in the protocol. Alternatively, an explanation of why a DMC is not needed	20,25
	21b	Description of any interim analyses and stopping guidelines, including who will have access to these interim results and make the final decision to terminate the trial	14-15
Harms	22	Plans for collecting, assessing, reporting, and managing solicited and spontaneously reported adverse events and other unintended effects of trial interventions or trial conduct	20-21
Auditing	23	Frequency and procedures for auditing trial conduct, if any, and whether the process will be independent from investigators and the sponsor	20

Ethics and dissemination

Research ethics approval	24	Plans for seeking research ethics committee/institutional review board (REC/IRB) approval	20-21
Protocol amendments	25	Plans for communicating important protocol modifications (eg, changes to eligibility criteria, outcomes, analyses) to relevant parties (eg, investigators, REC/IRBs, trial participants, trial registries, journals, regulators)	20-21
Consent or assent	26a	Who will obtain informed consent or assent from potential trial participants or authorised surrogates, and how (see Item 32)	8
	26b	Additional consent provisions for collection and use of participant data and biological specimens in ancillary studies, if applicable	N/A
Confidentiality	27	How personal information about potential and enrolled participants will be collected, shared, and maintained in order to protect confidentiality before, during, and after the trial	14,21,26 Appendix 2

review only

Declaration of interests	28	Financial and other competing interests for principal investigators for the overall trial and each study site	26
Access to data	29	Statement of who will have access to the final trial dataset, and disclosure of contractual agreements that limit such access for investigators	26
Ancillary and post-trial care	30	Provisions, if any, for ancillary and post-trial care, and for compensation to those who suffer harm from trial participation	26
Dissemination policy	31a	Plans for investigators and sponsor to communicate trial results to participants, healthcare professionals, the public, and other relevant groups (eg, via publication, reporting in results databases, or other data sharing arrangements), including any publication restrictions	21
	31b	Authorship eligibility guidelines and any intended use of professional writers	21
	31c	Plans, if any, for granting public access to the full protocol, participant-level dataset, and statistical code	26
**Appendices**			
Informed consent materials	32	Model consent form and other related documentation given to participants and authorised surrogates	Appendix 2
Biological specimens	33	Plans for collection, laboratory evaluation, and storage of biological specimens for genetic or molecular analysis in the current trial and for future use in ancillary studies, if applicable	N/A

*It is strongly recommended that this checklist be read in conjunction with the SPIRIT 2013 Explanation & Elaboration for important clarification on the items. Amendments to the protocol should be tracked and dated. The SPIRIT checklist is copyrighted by the SPIRIT Group under the Creative Commons "Attribution-NonCommercial-NoDerivs 3.0 Unported" license.

Effectiveness and cost-effectiveness of a web-based cardiac rehabilitation programme for people with chronic stable angina: protocol for the ACTIVATE (Angina Controlled Trial Investigating the Value of the 'Activate your heart' Therapeutic E-intervention) randomised controlled trial

Journal:	*BMJ Open*
Manuscript ID	bmjopen-2024-084509.R1
Article Type:	Protocol
Date Submitted by the Author:	20-Feb-2024

Complete List of Authors:	Williams, Nefyn; University of Liverpool, Primary Care and Mental Health<br>Collins, Brendan; University of Liverpool, Public Health Policy and Systems<br>Comerford, Terence; National Institute for Health and Care Research Applied Research Collaboration North West Coast, Patient and Public Involvement<br>Dodd, Susanna; University of Liverpool, Biostatistics<br>Fisher, Michael; Liverpool University Hospitals NHS Foundation Trust<br>Hardwick, Ben; University of Liverpool, Liverpool Clinical Trials Centre<br>Hennessy, Sophie; University of Liverpool Faculty of Health and Life Sciences, Liverpool Clinical Trials Centre<br>Jolly, Kate; University of Birmingham, Institute of Applied Health Research<br>Jones, Ian; Liverpool John Moores University,<br>Lane, Deirdre; University of Liverpool,<br>Lip, Gregory; University of Liverpool, Liverpool Centre for Cardiovascular Science<br>Morgan, Erica; National Institute for Health and Care Research Applied Research Collaboration North West Coast, Patient and Public Involvement<br>Ralph, Penelope; University of Liverpool Faculty of Health and Life Sciences, Department of Primary Care and Mental Health<br>Thijssen, Dick; Liverpool John Moores University<br>Singh, Sally; University Hospitals of Leicester NHS Trust, Cardiac/Pulmonary Rehabilitation

**Primary Subject Heading**:	General practice / Family practice
Secondary Subject Heading:	Cardiovascular medicine, Rehabilitation medicine
Keywords:	Ischaemic heart disease < CARDIOLOGY, Primary Care < Primary Health Care, REHABILITATION MEDICINE, Randomized Controlled Trial, Adult cardiology < CARDIOLOGY, HEALTH ECONOMICS

Effectiveness and cost-effectiveness of a web-based cardiac rehabilitation programme for people with chronic stable angina: protocol for the ACTIVATE (Angina Controlled Trial Investigating the Value of the 'Activate your heart' Therapeutic E-intervention) randomised controlled trial

Nefyn H Williams[1,6]

Email: Nefyn.williams@liverpool.ac.uk

Brendan Collins[1]

Email: brenc@liverpool.ac.uk

Terence Comerford[2]

Email: tjc57b@gmail.com

Susanna Dodd[1]

Email: s.r.dodd@liverpool.ac.uk

Michael Fisher[3,6]

Email: Michael.Fisher@liverpoolft.nhs.uk

Ben Hardwick[1]

Email: ben121@liverpool.ac.uk

Sophie Hennessy[1]

Email: Sophie.hennessy@liverpool.ac.uk

Kate Jolly[4]

Email: c.b.jolly@bham.ac.uk

Ian Jones[5,6]

Email: i.d.jones@ljmu.ac.uk

Deirdre A Lane[1,6]

For peer review only

Email: deirdre.lane@liverpool.ac.uk

Gregory YH Lip[6]

Email: gregory.lip@liverpool.ac.uk

Erica Morgan[2]

Email: ericamorgan@me.com

Penelope Ralph[1]

Email: p.ralph@liverpool.ac.uk

Dick Thijssen[5,6]

Email: D.Thijssen@ljmu.ac.uk

Sally J Singh[7]

Email: sally.singh@uhl-tr.nhs.uk

[1]University of Liverpool, UK

[2]National Institute for Health and Care Research (NIHR), Applied Research Collaboration North West Coast (ARC NWC), Patient and Public Involvement

[3]Liverpool University Hospitals NHS Foundation Trust, UK

[4]University of Birmingham, UK

[5]Liverpool John Moores University, UK

[6]Liverpool Centre for Cardiovascular Science at University of Liverpool, Liverpool John Moores University and Liverpool Heart and Chest Hospital, UK

[7]Department of Respiratory Sciences, University of Leicester, Leicester, UK

Correspondence to:

Professor Nefyn H Williams

For peer review only - http://bmjopen.bmj.com/site/about/guidelines.xhtml

Department of Primary Care and Mental Health, University of Liverpool, 1st Floor, Block B,

Waterhouse Building, 1-5 Brownlow Street, Liverpool, L69 3GL, UK

Email: nefyn.williams@liverpool.ac.uk

For peer review only

For peer review only - http://bmjopen.bmj.com/site/about/guidelines.xhtml

Abstract

Introduction

Chronic stable angina is common and disabling. Cardiac rehabilitation is routinely offered to people following myocardial infarction or revascularisation procedures and has the potential to help people with chronic stable angina. However, there is insufficient evidence of effectiveness and cost-effectiveness for its routine use in this patient group. The objectives of this study are to compare the effectiveness and cost-effectiveness of the 'Activate Your Heart' cardiac rehabilitation programme for people with chronic stable angina compared with usual care.

Methods and analysis

ACTIVATE is a multicentre, parallel-group, two-arm, superiority, pragmatic randomised controlled trial, with recruitment from primary and secondary care centres in England and Wales and a target sample size of 518 (1:1 allocation; allocation sequence by minimisation programme with built in random element). The study uses secure web-based allocation concealment. The two treatments will be optimal usual care (control) and optimal usual care plus the 'Activate Your Heart' web-based cardiac rehabilitation programme (intervention). Outcome assessment and statistical analysis will be performed blinded; participants will be unblinded. Outcomes will be measured at baseline and at 6 and 12 months' follow-up. Primary outcome will be the UK version of Seattle Angina Questionnaire (SAQ-UK), physical limitations domain at 12 months' follow-up. Secondary outcomes will be the remaining two domains of SAQ-UK, dyspnoea, anxiety and depression, health utility, self-efficacy, physical activity and the incremental shuttle walk test. All safety events will be recorded, and serious adverse events assessed to determine whether they are related to the intervention and expected. Concurrent economic evaluation will be cost-utility analysis from health service

For peer review only - http://bmjopen.bmj.com/site/about/guidelines.xhtml

perspective. An embedded process evaluation will determine the mechanisms and processes that explain the implementation and impacts of the cardiac rehabilitation programme.

Ethics and dissemination

North of Scotland NHS Research Ethics Committee approval, reference 21/NS/0115. Participants will provide written informed consent. Results will be disseminated by peer-reviewed publication.

Trial registration

ISRCTN10054455 (registered 16/09/2021).

Strengths and limitations of this study

- Pragmatic randomised controlled trial of an established cardiac rehabilitation programme for individuals with chronic stable angina, with unblinded participants and rehabilitation providers.
- Concurrent economic evaluation with a health service perspective.
- Embedded process evaluation to determine the mechanisms and processes that explain the implementation and impacts of the enhanced rehabilitation programme.
- The web-based intervention may not be acceptable to some people, but a paper version is available.

For peer review only - http://bmjopen.bmj.com/site/about/guidelines.xhtml

INTRODUCTION

Angina is chest pain usually caused by atherosclerosis of the coronary arteries, which restricts blood flow to the myocardium, especially when oxygen demand is increased during exercise. Angina is defined as stable if chest pain occurs predictably on physical exertion, or with emotion, and settles promptly with rest or after taking sublingual nitrate medication. Affected individuals have an increased risk of acute coronary events such as myocardial infarction (MI) [1]. Management consists of behaviour change advice to reduce risk factor profile, drug treatment and revascularisation procedures [2,3].

Cardiac rehabilitation is routinely offered to patients following MI, or revascularisation procedures, and consists of co-ordinated activities to improve the underlying cause and restore optimal functioning and slow, or reverse, disease progression [4]. The most prevalent components of cardiac rehabilitation are behaviour change and exercise training, but comprehensive cardiac rehabilitation programmes also include education, interventions to improve mental health, involvement of friends and family members and the development of long-term strategies beyond participation in the cardiac rehabilitation programme. Despite international guidance [2,5], such programmes are not offered routinely to people with chronic stable angina [6]. In the UK, the National Institute for Health and Care Excellence (NICE) does not support cardiac rehabilitation in this group until stronger evidence of effectiveness and cost-effectiveness is available [3]. Although there is good evidence for the effectiveness of cardiac rehabilitation for heart failure [7], or following MI or revascularisation procedures [8], rates of uptake and long-term adherence are low, especially in women, ethnic minority groups, and in areas of high deprivation [6].

For peer review only - http://bmjopen.bmj.com/site/about/guidelines.xhtml

For peer review only

A systematic review of exercise-based cardiac rehabilitation for adults with stable angina [9], identified seven randomised controlled trials (RCTs) (n=581) with high risk of bias. It found low quality evidence that cardiac rehabilitation resulted in a small improvement in exercise capacity compared with usual care with standard mean difference 0.45 (95% confidence intervals [CI] 0.2 to 0.7), and uncertainty about the effect on all-cause mortality, acute MI, cardiovascular-related hospital admissions, quality of life (QoL) and cardiac rehabilitation-related adverse events. These RCTs were small in size and recruited mainly white middle-aged men. More evidence is needed from under-represented groups including women, older age-groups, ethnic minorities and those with co-morbid conditions. Web-based programmes might increase the reach of cardiac rehabilitation, and there has been one small RCT (n=94) of such a programme ('Activate Your Heart'), which demonstrated that it was possible to provide cardiac rehabilitation to people with chronic stable angina [10]. Trial methods were feasible in terms of recruitment, retention and outcome measurement. After six months there were statistically significant lower levels of angina frequency, but a larger RCT is needed to test longer-term effectiveness and cost-effectiveness.

‘Activate Your Heart’ has the potential to improve physical and mental health with minimal risk. There may be a risk of injury or provoking an episode of angina when exercising, however the physical activity goals are carefully graded and the programme has an excellent safety record [10].

For peer review only - http://bmjopen.bmj.com/site/about/guidelines.xhtml

Aims and objectives

We aim to assess whether a web-based cardiac rehabilitation programme can improve the health of people with chronic stable angina, whether it is good value for money and delivered as intended.

The primary objective is to determine the effectiveness of the 'Activate Your Heart' web-based cardiac rehabilitation programme (or a paper version for those who prefer or who do not have internet access or device), for people with chronic stable angina compared with usual care in terms of angina-related health status, at 12 months' follow-up.

Secondary objectives:

- To estimate the cost-effectiveness of the cardiac rehabilitation programme for people with chronic stable angina compared with usual care in a cost-utility analysis with a health service perspective;
- To investigate the mechanisms and processes that explain the implementation and impacts of the cardiac rehabilitation programme in the intervention group and usual care in the control group;
- To identify the barriers and facilitators of uptake and completion of the rehabilitation programme with a focus on equity.

METHODS AND ANALYSIS

Trial design

For peer review only - http://bmjopen.bmj.com/site/about/guidelines.xhtml

ACTIVATE is a multisite, parallel-group, two-armed, superiority RCT with 1:1 allocation, stratified by gender and recruitment site (Figure 1). A pragmatic RCT of effectiveness compared with usual care, with blinded outcome assessment and statistical analysis; unblinded participants and rehabilitation providers. An internal pilot phase will assess feasibility. There will be a concurrent economic evaluation with a health service perspective. An embedded process evaluation will use mixed methods to examine the mechanisms and processes that explain the implementation and impacts of the enhanced rehabilitation programme, including an examination of barriers leading to health inequities. The Health Inequalities Assessment Toolkit (www.hiat.org.uk) informed trial design. The RCT was registered on 16 September 2021 (ISRCTN10054455). Trial registration data can be found in Appendix 1.

Trial setting and selection of sites/clinicians

Participants will be recruited from primary and secondary care in areas of England and Wales with high disease burden and socio-economic deprivation. Many of these areas include high proportions of ethnic minority groups. The web-based rehabilitation intervention (or paper version) will be delivered in the community.

Trial population

Inclusion criteria

- Adults with chronic stable angina with at least two out of three of the following features: constricting central chest pain, precipitated by exertion or emotional stress, relieved by rest or glyceryl trinitrate spray.

For peer review only - http://bmjopen.bmj.com/site/about/guidelines.xhtml

- Evidence of myocardial ischaemia from either a past medical history of acute coronary syndrome, myocardial infarction (MI) or revascularisation procedure at least 12 months previously; or from imaging studies such as invasive coronary angiography, computerised tomography angiography or myocardial perfusion testing.
- Revascularisation procedures not planned and managed with medical treatments only, including people with previous MI, or previous revascularisation procedure who may have attended cardiac rehabilitation in the past.

Exclusion criteria

- History of MI, revascularisation or cardiac rehabilitation participation within the last 12 months.
- Significant co-morbidities (as deemed by person confirming eligibility) that would limit participation in the exercise-based rehabilitation programme.
- Refractory angina on maximal medical therapy.

Trial treatment/interventions

We plan to compare web-based cardiac rehabilitation in addition to optimal usual care (intervention) with optimal usual care alone.

Optimal usual care

Usual care will be optimised in all participants. All participants will be seen by their GP or cardiology team, using a protocol informed by NICE guidance [3] to ensure that all are receiving optimal care. Considering the pragmatic nature of the proposed RCT, no

For peer review only - http://bmjopen.bmj.com/site/about/guidelines.xhtml

restrictions will be placed on usual care. NICE guidance [3] recommends that patients with chronic stable angina receive adequate information and support, lifestyle advice about smoking, exercise, diet and weight control. Episodes of angina should be treated with short-acting nitrate medication. Anti-anginal drug treatment to prevent episodes of angina consist of a beta-blocker or calcium channel blocker in the first instance; second line drugs (such as ivabradine, ranolazine) will be used as needed. In addition, drugs for secondary prevention of cardiovascular disease such as MI or stroke, include aspirin, angiotensin-converting enzyme inhibitor (especially if diabetes is present), statins in line with NICE guidance (and treatment targets) on lipid modification [11], and anti-hypertensives in line with NICE guidance (and treatment targets) on hypertension [12].

Concomitant medication will be recorded as part of the health economic data collection. There are no restrictions on any medications. Participants will not have participated in a cardiac rehabilitation programme over the previous 12 months, but other schemes such as smoking cessation, exercise referral and weight management schemes may be available and accessible to participants. Where participants have engaged in one or more of these it will be recorded.

Cardiac rehabilitation intervention

Usual care will be optimised as described above. In addition, participants in the intervention arm will be given access to 'Activate Your Heart' (www.activateyourheart.org.uk), which is an online interactive, secure and password-protected website designed for participants to use at home. The programme was co-produced with health care professionals, patients/members of the public and software designers. The programme aims to deliver the

For peer review only - http://bmjopen.bmj.com/site/about/guidelines.xhtml

five core components described by the British Association for Cardiovascular Prevention and Rehabilitation (BACPR): health behaviour change and education, lifestyle risk factor management, medical risk factor management, psychosocial health and long-term management [4]. The programme uses the following behaviour change techniques: goal setting, self-monitoring, feedback on behaviour, graded tasks, social reward, providing information about health consequences, and reducing negative emotions. The programme is tailored to individual need, organised in four stages, which can be completed in 8 weeks, but access to the site and its features continues for 12 months. Before beginning the programme, each participant sees a member of cardiac rehabilitation staff who provides training on the website either face-to-face, or remotely by telephone or video consultation. The participant will complete an online registration form, which records their medical history and cardiac risk factors. A paper-based manual is available for participants who are unable or unwilling to use the online rehabilitation system [13]. The website generates a tailored plan from this information, consisting of individualised goals for exercise, diet (eating more fruit and vegetables and reducing salt), emotions (managing stress, anxiety and low mood) and smoking. Adherence with these goals is regularly assessed by a short set of questions and feedback on performance. As the participant progresses through the programme, goals become increasingly difficult. Each participant records details of their daily exercise in an online diary, and regular feedback is given. Smokers are provided with feedback about the amount of money they are saving. The programme also contains written information about the health consequences of heart disease and risk factors (exercise, diet, sexual activity, driving, returning to work, hobbies, holidays, benefits, smoking, anxiety and mood). In addition, the programme reduces negative emotions by providing advice about stress reduction and anxiety management skills. There is access to an online discussion

For peer review only - http://bmjopen.bmj.com/site/about/guidelines.xhtml

forum, and 'ask the expert' email facility. Cardiac rehabilitation staff can monitor participants' progress and respond to questions posted via e-mail. After 12 weeks the cardiac rehabilitation staff member will see the participant again to discuss progress and local exercise opportunities. Cardiac rehabilitation staff will report any safety events that they become aware of.

Outcomes

Participants will complete outcome measures at baseline, 6 and 12 months, administered by practice staff blinded to participant allocation, which are from the standardised outcome measurement for patients with coronary artery disease, agreed by the International Consortium for Health Outcomes Measurement [14] (Tables 1 and 2). The primary outcome will be the UK Version of Seattle Angina Questionnaire (SAQ-UK) Physical Limitation domain [15] assessed at 12 months' follow-up. Participant completed secondary outcomes will include the remaining two domains of the SAQ-UK [15], MRC dyspnoea scale [16], Hospital Anxiety and Depression Scale (HADS) [17], and General Self-Efficacy Scale [18]. Physical activity will be measured where possible with the ActivPAL accelerometer [19] for 7 days, supplemented by the International Physical Activity Questionnaire [20]. Cardiovascular fitness will be measured with the Incremental Shuttle Walk Test [21]. Economic measures will be EuroQol EQ-5D-5L [22] and Client Service Receipt Inventory (CSRI) [23] as well as intervention costs. Baseline assessments will also include height, weight, blood pressure and serum cholesterol within the last 12 months.

Qualitative interviews will take place with a selection of participants at the end of the 12 week cardiac rehabilitation programme among those receiving the intervention, and

For peer review only - http://bmjopen.bmj.com/site/about/guidelines.xhtml

between months 11 and 12 with the same sample of participants in the intervention group and an additional sample from the control group. These will gather data on trial participation and intervention design (see process evaluation below).

Routinely collected demographic, clinical and recruitment data will include the number of participants who are eligible, willing to be randomised, withdraw after randomisation, complete outcome measurements, reasons for non-completion, age and gender.

Sample size

In order to detect the minimum clinically important difference of 8 points for the SAQ-UK physical limitation domain, assuming a standard deviation of 25 [15], with 90% power at the 5% significance level, 207 participants per group will be required (calculated using nQuery 8). In order to allow for up to 20% loss to follow up, we aim to recruit 259 participants per group (518 in total). This sample size will give >99% power for secondary outcomes.

Recruitment and randomisation

Screening and consent

Written informed consent will be sought from potential participants who will be approached by the study team and invited to consider participation, following a search of their health record for eligibility. Where possible, potential participants will be introduced to the study during a face-to-face appointment with clinical staff. Potential participants can also be approached by a member of the local research team by written invitation and followed up by a phone call or text message after a week if there has been no response. Potential participants will be invited to attend a screening appointment. If they are eligible, and

For peer review only

interested in the trial, the trial team researchers would then recruit participants following the trial's informed consent process. These assessments will be recorded in a screening log, including any reasons for ineligibility. Copies of the participant information sheet and informed consent form can be found in Appendix 2.

Randomisation procedures

Participants will be randomised via a secure (24-hour) web-based randomisation system controlled centrally by the clinical trials unit to receive either usual care or tailored cardiac rehabilitation programme ('Activate Your Heart') plus usual care. Randomisation will have an allocation ratio of 1:1 and use a minimisation programme with a built in random element, stratified by site and gender. Randomisation should occur no more than 2 weeks after confirming eligibility, obtaining informed consent, and completing baseline assessments. Following allocation, participants will be notified of their allocation as soon as possible and then receive their randomised treatment allocation.

Blinding

This is a pragmatic trial comparing the 'Activate Your Heart' cardiac rehabilitation intervention with usual care, so it will not be possible to blind participants or their rehabilitation providers to treatment group allocation. However, collection of outcome measures and data analysis will be performed blind to treatment allocation. We will include a perception of allocation form for the person collecting the outcome measures to complete, to monitor the level of blinding achieved for these researchers.

Statistical analysis

For peer review only - http://bmjopen.bmj.com/site/about/guidelines.xhtml

Final analysis will take place once all participants have been followed-up for 12 months, and the database has been locked. Analyses will be by 'intention to treat' for the primary and secondary outcomes on all randomised participants, in the group to which they were allocated, and for whom the outcomes of interest have been observed or measured. A full and detailed statistical analysis plan (SAP) will be finalised prior to any comparative analysis of the groups; the main features of the SAP are described briefly here.

Baseline

Demographic and clinical characteristics will be summarised separately using descriptive statistics for each randomised group to allow assessment of whether balance was achieved between randomised groups. This will include gender, ethnicity, socio-economic status and other relevant PROGRESS-Plus characteristics [24]. No statistical testing of differences between groups will be performed.

Analysis of effectiveness

Primary and secondary outcomes at baseline, 6 months' and 12 months' follow-up will be summarised for each treatment group using descriptive statistics at each time point. If normally distributed, the difference between group means (with 95% confidence intervals) will be reported from an analysis of covariance (ANCOVA) adjusted for baseline score and stratification factors.

Missing data and withdrawals

Predictors of missing data will be investigated using regression models (including age, living arrangements and co-morbidities) and any significant predictors will be considered for inclusion in the models. In addition, given the two assessment points at 6 and 12 months, a sensitivity analysis will be conducted using a joint modelling approach to check whether

For peer review only - http://bmjopen.bmj.com/site/about/guidelines.xhtml

For peer review only

there is any difference in outcome, using longitudinal outcome rather than the outcome at 6 months or 12 months alone, between the randomised arms, adjusted for dropouts or missing values.

Instrumental variable regression

The causal impact of engagement with the intervention will be assessed in an instrumental variable regression model, using the number of stages completed in the rehabilitation programme, and the number of times that the programme is accessed, as measures of intervention use.

Mediation analyses

The hypothesised mechanism of change for the rehabilitation intervention is that participants' primary outcome (physical limitation) is mediated by self-efficacy and physical activity. If the rehabilitation intervention has a significant effect on the primary outcome ($p<0.05$) in ANCOVA, causal mediation analysis will be used to determine whether these potential mediators predict change in SAQ-UK Physical Limitation domain at 12 months.

Economic analysis

The economic evaluation aims to estimate the cost-effectiveness of the 'Activate Your Heart' online rehabilitation programme plus optimal usual care when compared with optimal usual care alone. The rehabilitation programme will be fully costed using unit costs from a health service perspective. Unit costs will be obtained from national sources of reference costs and applied to information received from pilot questionnaires, namely salary band of cardiac rehabilitation therapists, time spent with the participant at the initial and final rehabilitation appointments, costs of travel and the costs of registering with the 'Activate Your Heart' online cardiac rehabilitation programme. Participants' health service activity data will be

For peer review only - http://bmjopen.bmj.com/site/about/guidelines.xhtml

For peer review only

obtained from the CSRI [23] and unit costs from national sources [25, 26]. The costs of service use and the cost of the intervention will be added together for use in a cost-effectiveness analysis. We will account for the likely skewed nature of cost data in any regression carried out, by using Tweedie regression and/or beta distributions. The EQ-5D-5L scores will be converted into a health utility weight using UK norms. The base-case analysis will be conducted using the Van Hout scoring algorithm [27], with sensitivity analysis conducted using the EQ-5D-5L value set for England [28]. Utility indices will be used to calculate QALYs over the 12 month trial period, using the area under the curve approach, with regression-based adjustment for baseline EQ-5D utility scores [29,30]. The health economic results will initially be presented as disaggregated tables of costs alongside the health consequences. In these tables, we will also describe the costs and consequences of the rehabilitation intervention in the underserved groups recruited, such as women, ethnic minorities and those of low socio-economic status.

Costs and consequences will then be combined in a cost-utility analysis, to estimate the cost per additional QALY gained associated with each of the compared options. Deterministic and probabilistic sensitivity analyses will be undertaken to explore the robustness of the obtained results to sample variability and plausible variations in key assumptions and employed analytical methods. Cost-effectiveness acceptability curves will be constructed to show the probability that the intervention is cost-effective at specific thresholds of cost per QALY gained such as NICE threshold range of £20,000 to £30,000 per QALY gained [31]. We will develop a two-stage economic model; a decision tree to reflect the outcomes of the trial (within-trial economic evaluation) and a Markov state transition model to extrapolate the evolution of patient outcome and costs over a lifetime horizon (model-based economic

For peer review only - http://bmjopen.bmj.com/site/about/guidelines.xhtml

evaluation). Value of information analysis will be conducted after taking into account uncertainty derived from decision-analytic models.

Process evaluation

The concurrent mixed methods process evaluation will aim to identify and explain the mechanisms and processes that enabled or acted as a barrier to the implementation of the cardiac rehabilitation intervention [32,33]. The process evaluation will help build a picture of how the intervention was carried out in reality and what factors shaped it. By conducting a process evaluation, it will be possible to identify if observed impacts are solely due to the cardiac rehabilitation programme, or if these impacts are a result of external and internal variables closely linked to the environment and the context in which the intervention takes place. More specifically, the process evaluation will examine the recruitment of sites and rehabilitation teams, recruitment and reach in participants (from the recruitment log), intervention delivery to individuals (coverage, intensity), participation, retention, response of individuals to the rehabilitation intervention (data collected by the 'Activate Your Heart' website), unintended consequences (adverse events and inequities), contextual factors and sustained motivation for behaviour change. The website will be examined for the degree of completion of the programme, number of occasions that the website was accessed, and the extent of the change in behaviour(s) recorded. There will be a particular focus on underserved groups (women, ethnic minorities, low socio-economic status, other PROGRESS-Plus [24] characteristics and those without internet access or device).

Semi-structured telephone or video interviews of participants (n=30) with purposive sampling to ensure diversity based on age, gender, functional impairment, treatment group,

For peer review only - http://bmjopen.bmj.com/site/about/guidelines.xhtml

site, ethnicity and mode of delivery. A topic guide will be developed with the assistance of our Public Advisory Group, and will ask about the acceptability of the rehabilitation programme and the barriers and facilitators of uptake and completion, such as family support, health inequities, lack of facilities, lack of transport, etc. However, topic guides will be iterative and informed by earlier interviews and adapted accordingly. There will be a particular focus on the acceptability and barriers to implementation in underserved groups (women, ethnic minority and low socio-economic status). Interviews will take place at the end of the 12 week cardiac rehabilitation programme among those receiving the intervention, and between months 11 and 12 in both groups. We will interview a minimum of 10 cardiac rehabilitation staff at different time-points, to gain insight into the early, medium and longer term delivery of the cardiac rehabilitation programme. All of the interviews will be audio recorded and fully transcribed. Participant contact with the online discussion forum, and the 'ask the expert' email facility will also be examined. We will also examine data collected routinely by the website for the purposes of the national audit for cardiac rehabilitation. Qualitative data will be analysed using a thematic analysis approach [34]. Quantitative data (recruitment logs, use of the website, recorded change in behaviours, national audit data) will be analysed using descriptive statistics to allow for the exploration of frequency of responses. All data sets will be synthesised in order to describe the complex nature of the enhanced rehabilitation intervention.

Patient and public involvement

There has been patient and public involvement (PPI) at all stages including developing the research question, choosing outcomes relevant to patients, commenting on the burden of the intervention and of trial participation. Two PPI co-investigators are active members of

For peer review only - http://bmjopen.bmj.com/site/about/guidelines.xhtml

the trial management group and help to convene a public advisory group which meets throughout the trial to advise on trial procedures, especially patient facing materials, promotional videos in multiple languages and the dissemination plan. Members of the PPI group recorded voice-overs for the promotional video in different languages.

ETHICS AND DISSEMINATION

NHS research ethics approval was obtained from North of Scotland Research Ethics Committee, reference 21/NS/0116. The current protocol is version 5 (04/05/2023). Participants will provide written informed consent. A Trial Steering Committee is providing overall supervision, and an Independent Data Safety and Monitoring Committee is responsible for reviewing and assessing recruitment, interim monitoring of safety and effectiveness, trial conduct and external data.

All safety events will be recorded by researchers when they are made aware of the event by the participant, treating clinician, or therapist. Adverse event reports and serious adverse events (SAEs) not related to the intervention will be entered on to the remote data entry system. Each SAE will be assessed by the relevant PI to determine whether it is related to the intervention. A related SAE will be assessed by the CI to determine whether it is expected. If the SAE is related and unexpected (RUSAE) it will be reported to the Research Ethics Committee (REC) and sponsor in an expedited manner.

Reporting of the trial will be consistent with the CONSORT 2010 Statement (patient reported outcomes and non-pharmacological interventions) [35]. We will submit the final report to a peer-reviewed academic journal, according to our publication strategy and

For peer review only - http://bmjopen.bmj.com/site/about/guidelines.xhtml

authorship policy. Research data will be available for secondary analysis upon reasonable request.

Trial status

The first participant was recruited in July 2022. At the time of submission this trial had been open in 20 sites, with 10 more sites in preparation, and had recruited 64 participants. Updated recruitment information can be found on the trial website (http://activate-trial.org.uk).

Abbreviations

ANCOVA	Analysis of Covariance
ARC NWC	Applied Research Collaboration North West Coast
BACPR	British Association for Cardiovascular Prevention and Rehabilitation
CI	Chief Investigator
CONSORT	Consolidated Standards of Reporting Trials.
CSRI	Client Service Receipt Inventory
EQ-5D-5L	EuroQol 5 Dimensions 5 Levels
GP	General Practitioner
HADS	Hospital Anxiety and Depression Scale
IPAQ	International Physical Activity Questionnaire
ISRCTN	International Standard Randomised Controlled Trial Number
IV	Instrumental Variable

For peer review only - http://bmjopen.bmj.com/site/about/guidelines.xhtml

LCTC	Liverpool Clinical Trials Centre
MI	Myocardial Infarction
MRC	Medical Research Council
NHS	National Health Service
NICE	National Institute of Health and Clinical Excellence
NIHR	National Institute for Health and Care Research
PCI	Percutaneous Coronary Intervention
PI	Principal Investigator
PPI	Patient and Public Involvement
QALY	Quality Adjusted Life Year
QoL	Quality of Life
RCT	Randomised Controlled Trial
REC	Research Ethics Committee
RUSAE	Related Unexpected Serious Adverse Event
SAE	Serious Adverse Event
SAQ-UK	United Kingdom version of Seattle Angina Questionnaire

For peer review only

For peer review only - http://bmjopen.bmj.com/site/about/guidelines.xhtml

Contributors

NHW is the chief investigator and grant holder, and SJS is the joint lead investigator and they are responsible for study design, conduct and analysis, have overall responsibility for the study and act as guarantors. SH is the trial co-ordinator and BH the senior trial co-ordinator overseeing day-to-day conduct, and providing methodological input. SDod will write the statistical analysis plan. BC will write the health economic analysis plan. DAL and PR will write the process evaluation analysis plan with NHW. EM and TJC are Patient and Public Involvement co-investigators. GYHL, MF, TJ and KJ are co-investigators responsible for study design, methodological oversight and provide cardiology (GYHL, MF), exercise science (DT), cardiac nursing (IJ) and primary care (NHW, KJ) expertise. All authors were involved in drafting, revising and approving this manuscript.

Acknowledgements

The authors would like to thank all of the principal investigators, research nurses, hospital staff, cardiac rehabilitation teams, leisure centre and exercise referral scheme staff: Dr Timothy Smith (Pendle Medical Partnership,) Lisa Robson (Pendle View Medical Centre,) Dr Tanveer Ahmed Dr Eve Frost (Shifa Surgery,) Vishal Mashru (Billesdon Surgery, Croft Medical Centre, South Leicestershire Medical Group,) Dr Selena Harris, Maisie Wrightson (Clarence House, Dr Jenny Liddell (Corwen Medical Centre,) Dr Joanna Le Noury (Plas Menai Surgery), Dr Helen Alefounder, Dr Jessica Deacon, Lucie Parrie, Chantal Stewart (West End Medical Centre,) Dr Nadja Van Ginneken, Theresa Nuttall, Jessica Keane (Brownlow Group,) Dr Stephen Forster (Heswall and Pensby Group Practice,) Dr David Lewis, Ann Kent, Carol Burgess, Carrie Geldhart (Vauxhall Primary Care Health Centre), Dr Helen Steiner, Stella Oldham (The Village Practice,) Dr Rebecca Sinfield, Hannah Mcgowan (St George's Medical

For peer review only - http://bmjopen.bmj.com/site/about/guidelines.xhtml

Centre,) Dr Gareth Powell, Michelle Morgan, Shirley Salter (Llan Healthcare,) Dr Michael Fisher, Laura Pennington, Anthony Burns, Daniel Evans, Ahmed Elamin (Liverpool University Hospitals NHS Foundation Trust), Professor Sally Singh, George Hudson, Donna Alexander (Leicester Hospitals NHS Trust.)

Special thanks to the LCTC head of data management and monitoring (Clare Jackson), LCTC information systems team (Sharon Keane, Janet Harrison, Keith Kennedy, Tim Charter), trial co-ordinator assistant (Eleanor MacDonald) and quality assurance managers (Katie Neville, Claire Johnson), and to the North West Coast Clinical Research Network.

We very much appreciate the work of the Trial Steering Committee Clare Wilkinson (chair), Robert Copeland (Independent Member) Rod Taylor (Independent Member), Katie Pike (Independent Statistician), Tracy Kitto (Independent Member), Keith Wilson (PPI Representative); Independent Data and Safety Monitoring Committee Umesh Chauhan (chair), Catriona Keerie (Independent Statistician), Ahmet Fuat (Independent Member), Adrian Taylor (Independent Member); PPI team Terrence Comerford, Ray Martin, Erica Morgan, Hameed Khan, Naheed Tahir, Robert Smart and Lynn Hedgecoe.

Funding

This work was funded by the National Institute for Health Research (NIHR) Health Technology Assessment Programme, grant number 131015. It was supported by the NIHR Applied Research Collaboration North West Coast, NIHR Clinical Research Network North West Coat and NIHR Leicester Biomedical Research Centre. SJS and GYHL are NIHR Senior investigators. KJ is part-funded by the NIHR Applied Research Collaboration West Midlands.

For peer review only

For peer review only - http://bmjopen.bmj.com/site/about/guidelines.xhtml

The views and opinions expressed therein are those of the authors and do not necessarily reflect those of the HTA, NIHR, NHS or the Department of Health.

Trial sponsor

University of Liverpool: sponsor's reference: UoL001635. Contact: Karen Wilding, Senior Clinical Research Governance Manager, University of Liverpool. The University has appropriate clinical trials and professional indemnity insurance.

Competing interests

NHW reports additional grants from NIHR HTA outside the submitted work and deputy chair of the NIHR HTA programme funding committee (commissioned research). DAL reports additional grants from the NIHR outside the submitted work and investigator-initiated educational grants from BMS and Pfizer. DAL has been a speaker for Bayer, Boehringer Ingelheim, and BMS/Pfizer and has consulted for BMS, and Boehringer Ingelheim; all outside the submitted work. GHYL has been a consultant and speaker for BMS/Pfizer, Medtronic, Boehringer Ingelheim and Daiichi-Sankyo. No fees were directly received personally. BC works part-time for Welsh Government outside of the submitted work. KJ was sub-committee chair of the NIHR Programme Grants for Applied Research until 2023. BH reports additional grants from the NIHR outside the submitted work and investigator-initiated grant from Novartis. All other authors declare no competing interests.

Data availability statement

For peer review only - http://bmjopen.bmj.com/site/about/guidelines.xhtml

Technical appendix, statistical code, and dataset will be made available after completion of the study and the reporting of the main results. The datasets generated will be stored in a non-publicly available repository and will be available from 31/07/2026 on request from the corresponding author. Consent was required and obtained from all participants. Data will be anonymised before they are shared without any ethical or legal restrictions.

For peer review only

For peer review only - http://bmjopen.bmj.com/site/about/guidelines.xhtml

References

1. Eisen A, et al. REACH Registry Investigators. Angina and Future Cardiovascular Events in Stable Patients with Coronary Artery Disease: Insights from the Reduction of Atherothrombosis for Continued Health (REACH) Registry.JAmHeartAssoc 2016 28;5(10).
2. Knuuti J, et al. 2019 ESC Guidelines for the diagnosis and management of chronic coronary syndromes: The Task Force for the diagnosis & management of chronic coronary syndromes of the European Society of Cardiology (ESC).EurHeartJ 2020 41:407–77.
3. National Institute for Health and Clinical Excellence. CG126. Stable angina: management. https://www.nice.org.uk/guidance/cg126/resources/stable-angina-management-pdf-35109453262021 Accessed 15 September 2023.
4. British Association for Cardiovascular Prevention and Rehabilitation (BACPR) Standards and Core Components for Cardiovascular Disease Prevention and Rehabilitation 2023 (4th Edition). https://www.bacpr.org/__data/assets/pdf_file/0021/64236/BACPR-Standards-and-Core-Components-2023.pdf Accessed 15 September 2023.
5. Fihn SD, et al. 2012 ACC/AHA/AATS/PCNA/SCAI/STS Guideline for the Diagnosis and Management of Patients With Stable Ischemic Heart Disease. J Am Coll Cardiol 2012; 60: e44-164.
6. British Heart Foundation. The National Audit of Cardiac Rehabilitation: Quality and Outcomes Report 2019. https://www.bhf.org.uk/informationsupport/publications/statistics/national-audit-of-cardiacrehabilitation-quality-and-outcomes-report-2019. Accessed 03 July 2020.
7. Taylor RS, et al. Exercise-based rehabilitation for heart failure. Cochrane Database of Systematic Reviews 2014, Issue 4. Art. No.: CD003331.
8. Heran BS, et al. Exercise-based cardiac rehabilitation for coronary heart disease. Cochrane Database of Systematic Reviews 2011, Issue 7. Art. No.: CD001800.
9. Long L, et al. Exercise-based cardiac rehabilitation for adults with stable angina. Cochrane Database of Systematic Reviews 2018, Issue 2. Art. No.: CD012786.
10. Devi R, Powell J, Singh S, A Web-Based Program Improves Physical Activity Outcomes in a Primary Care Angina Population: Randomized Controlled Trial J Med Internet Res 2014;16(9):e186 URL: https://www.jmir.org/2014/9/e186, DOI: 10.2196/jmir.3340
11. National Institute for Health and Clinical Excellence. CG181. Cardiovascular disease: risk assessment and reduction, including lipid modification. https://www.nice.org.uk/guidance/cg181/resources/cardiovascular-disease-risk-assessment-and-reduction-including-lipid-modification-pdf-35109807660997 Accessed 15 September 2023.
12. National Institute for Health and Clinical Excellence. CG136. Hypertension in adults: diagnosis and management. https://www.nice.org.uk/guidance/ng136/resources/hypertension-in-adults-diagnosis-and-management-pdf-66141722710213 Accessed 15 September 2023.
13. Warschauer M, Matuchinak T. New technology and digital worlds: Analyzing evidence of equity in access, use, and outcomes. Review of Research in Education 2010; 34: 179-225.
14. McNamara RL, et al. Standardized Outcome Measurement for Patients With Coronary Artery Disease: Consensus From the International Consortium for Health Outcomes Measurement (ICHOM). J Am Heart Assoc 2015; 4: e001767.
15. Garratt A, Hutchinson A, Russell IT. The UK version of the Seattle Angina Questionnaire (SAQ-UK): Reliability, validity and responsiveness. J Clin Epidemiol 2001; 54: 907-15.
16. MRC dyspnoea scale. https://www.ukri.org/councils/mrc/facilities-and-resources/find-an-mrc-facility-or-resource/mrc-dyspnoea-scale/ Accessed 08 April 2023.

For peer review only - http://bmjopen.bmj.com/site/about/guidelines.xhtml

17. Zigmond A, Snaith R. The Hospital Anxiety and Depression Scale. Acta Psychiatr Scand. 983; 67: 1983.
18. Schwarzer R, Jerusalem M. Generalized Self-Efficacy scale, Measures in health psychology: a user's portfolio. Causal and control beliefs. Windsor, UK: NFER-NELSON, 1995.
19. Edwardson CL, Winkler EAH, Bodicoat DH, Yates T, Davies MJ, Dunstan DW, Healy GN. Considerations when using the activPAL monitor in field-based research with adult populations. J Sport Health Sci 2017; 6: 162-178.
20. Craig CL, et al. International physical activity questionnaire: 12-country reliability and validity. Med Sci Sports Exerc 2003; 35: 1381-95.
21. Singh SJ, Morgan MDL, Scott S, Walters D, Hardman AE. Development of a shuttle walking test of disability in patients with chronic airways obstruction. Thorax 1992; 47: 1019-1024.
22. EuroQol Group. EuroQol – a new facility for the measurements of health-related quality of life. Health Policy 1990;16:199-208.
23. Database of Instruments for Resource Use Measurement. Client Service Receipt Inventory (CSRI). http://www.dirum.org/instruments/details/44. Accessed 31 January 2019.
24. O'Neill J, Tabish H, Welch V, Petticrew M, Pottie K, Clarke M, Evans T, Pardo J, Waters E, White H, Tugwell P. Applying an equity lens to interventions: using PROGRESS ensures consideration of socially stratifying factors to illuminate inequities in health. J Clin Epidemiol 2014; 67: 56-64.
25. Department of Health. NHS reference costs 2015 to 2016. https://www.gov.uk/government/publications/nhs-reference-costs-2015-to-2016 Accessed 03 July 2020.
26. Curtis L, Burns A. Unit costs of health and social care 2019. Personal Social Services Research Unit (PSSRU). https://www.pssru.ac.uk/project-pages/unit-costs/unit-costs-2019/ Accessed 03 July 2020.
27. van Hout B, et al. Interim Scoring for the EQ-5D-5L: Mapping the EQ-5D-5L to EQ-5D-3L Value Sets. Value in Health 2012; 15: 708-715.
28. Devlin NJ, Shah KK, Feng Y, Mulhern B, Van Hout B. Valuing health-related quality of life: An EQ-5D-5L value set for England. Health Economics. 2018;27(1):7-22.
29. Glick H, Doshi J, Sonnad S, Polsky D. Economic evaluation in clinical trials. Oxford: Oxford University Press; 2007.
30. Drummond MF, Sculpher MJ, Torrance GW, O'Brien BJ, Stoddart GL. Methods for the economic evaluation of health care programmes. Oxford: Oxford University Press; 2005.
31. National Institute for Health and Care Excellence. Guide to the methods of technology appraisal. http://www.nice.org.uk/article/pmg9/resources/non-guidance-guide-to-themethods-of-technology-appraisal-2013-pdf. 2013. Accessed on 03 July 2020.
32. Oakley, A., Strange V., Bonell C., Allen E, Stephenson J. Process evaluation in randomised controlled trials of complex interventions. Health Services Research 2006; 332: 413-416.
33. Moore GF, Audrey S, Barker M, Bond L, Bonell C, Hardeman W, Moore L, O'Cathain A, Tinati T, Wight D, Baird J. Process evaluation of complex interventions: Medical Research Council guidance. BMJ 2015; 350: h1258.
34. Braun V, Clarke V. Using thematic analysis in psychology. Qualitative Research in Psychology 2006; 3: 77-101.
35. Schulz KF, Altman DG , Moher D , et al. CONSORT 2010 statement: updated guidelines for reporting parallel group randomised trials. BMC Med 2010;8:18

For peer review only - http://bmjopen.bmj.com/site/about/guidelines.xhtml

Table 1. ACTIVATE outcome measures

**Patient Completed Measure - Primary**	**Description**	**Range**
UK version of Seattle Angina Questionnaire (SAQ-UK) Physical Limitation domain [15]	Measures how daily activities are limited by angina over the past 4 weeks. Items are weighted equally, summed and transformed to a 0-100 score. Higher score indicates better functioning.	(0-100)
**Patient Completed Measures - Secondary**		
UK version of Seattle Angina Questionnaire (SAQ-UK) Angina Frequency and Perception and Treatment Satisfaction domains [15]	Remaining two domains of SAQ-UK. Items are weighted equally, summed and transformed to a 0-100 score. Higher scores represent higher levels of health or satisfaction with treatment.	(0-100)
MRC dyspnoea scale [16]	Five item questionnaire that assesses the degree of shortness of breath with common activities. One point is assigned to each activity associated with dyspnoea. Higher scores indicates more limitation due to dyspnoea.	(0-5)
Hospital Anxiety and Depression Scale (HADS) [17]	Measures anxiety (7 items) and depression (7 items) in patients with physical health problems. Two sub-scales with higher scores indicating greater anxiety or depression.	(0-21)
General Self-Efficacy scale [18]	Measures optimistic self-belief to cope with a variety of difficult demands in life. Higher score indicates greater self-efficacy.	(10-40)
International Physical Activity Questionnaire (IPAQ) [20]	This questionnaire supplements the accelerometer data by providing self-reported duration (minutes) and frequency (days) of physical activity in the previous 7 days in the domains: job-related, transportation, housework, recreation, and sitting.	Minutes of light, moderate and vigorous physical activity

For peer review only - http://bmjopen.bmj.com/site/about/guidelines.xhtml

**Patient Completed Economic Measures**		
EuroQol EQ-5D-5L [22]	Health utility index with five dimensions (mobility, self-care, usual activities, pain/discomfort, anxiety/depression) and five levels to give health states converted to a utility weight. Also Visual Analogue Score (VAS) for health state today	Health utility weight from 0 (death) to 1.0 (perfect health) also with negative values VAS (0-100)
Client Service Receipt Inventory (CSRI) [23]	Collects information about health service use, including medication, smoking cessation, diet management, weight control programmes and exercise referral schemes.	According to activity
**Objective measures of physical activity and function**		
Physical activity measured with the ActivPAL accelerometer [19]	The ActivPAL device is a small, slim monitor worn on the thigh. It provides accelerometer-derived information about thigh position and acceleration to determine body posture (sitting, lying or upright), stepping, and stepping speed (cadence), from which energy expenditure can be inferred. It will be worn for 7 days to measure step count, energy expenditure, duration of sedentary, light, moderate and vigorous physical activity. Participants will be asked to keep a wear diary documenting working hours, sleep, removal reasons and any other comments.	Step count, energy expenditure, duration of sedentary, light, moderate and vigorous physical activity
Incremental Shuttle Walk Test [21]	Progressive walking test that requires the participant to walk up and down a 10 m course, at a speed dictated by an audio signal. The time between signals is reduced every minute and there are 12 levels. The test ends when the participant either (a) is too breathless to maintain the required speed or (b) fails to complete a shuttle in the time allowed or (c) reaches 85% of the predicted maximal heart rate.	Metres walked

For peer review only - http://bmjopen.bmj.com/site/about/guidelines.xhtml

Table 2. ACTIVATE protocol schedule of forms and procedures

Procedures	Screening	Baseline / Randomisation	7 days post randomisation*	Trial intervention**	26 weeks post randomisation	27 weeks post randomisation*	52weeks post randomisation	53 weeks post randomisation*
**Eligibility screening and consent**								
Assessment of eligibility	X							
Informed consent	X							
Confirm consent		X		X	X		X	
Randomisation		X						
Vital signs, height, weight, serum cholesterol		X						
**Outcome measurement**								
Seattle Angina Questionnaire		X			X		X	
MRC dyspnoea scale		X			X		X	
Hospital Anxiety and Depression Scale		X			X		X	
General Self-Efficacy scale		X			X		X	
EuroQol EQ-5D-5L		X			X		X	
Client Service Receipt Inventory		X			X		X	
Physical activity measured with ActivPal accelerometer		X	X*		X	X*	X	X*
International Physical Activity Questionnaire			X			X		X
Incremental shuttle walk test		X			X		X	
**Safety Event Reporting**								
Monitoring of Adverse Events			X	X	X	X	X	X
Monitoring of Serious Adverse Events			X	X	X	X	X	X

* Removal of ActivPal after 7 days

** If randomised to intervention arm, participants will be invited to meet with member of cardiac rehabilitation staff who will train the participant on how to use the intervention

For peer review only

Figure title

Figure 1. ACTIVATE participant flowchart

For peer review only

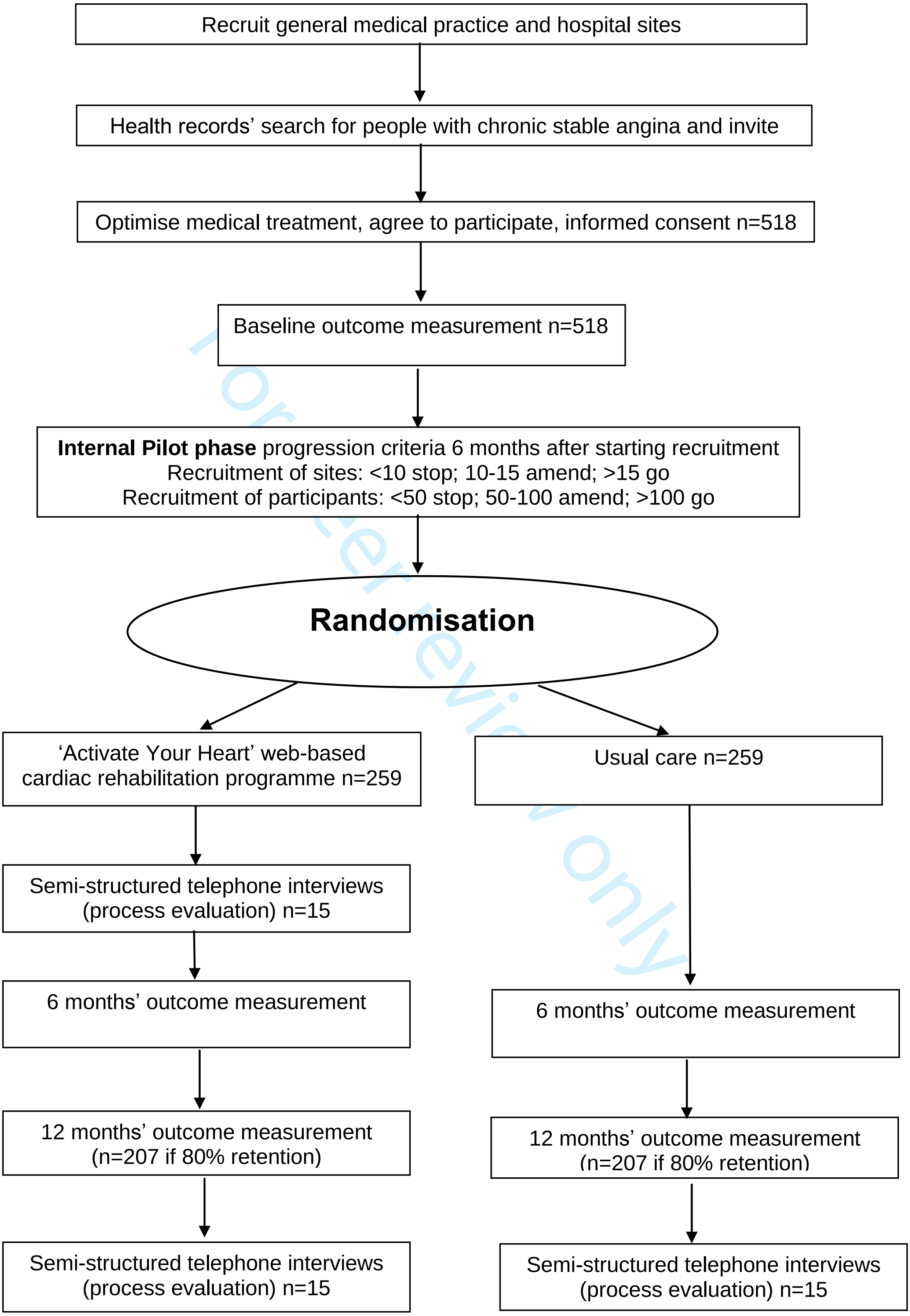
Recruit general medical practice and hospital sites
Health records' search for people with chronic stable angina and invite
Optimise medical treatment, agree to participate, informed consent n=518
Baseline outcome measurement n=518
Internal Pilot phase progression criteria 6 months after starting recruitment
Recruitment of sites: <10 stop; 10-15 amend; >15 go
Recruitment of participants: <50 stop; 50-100 amend; >100 go
Randomisation
'Activate Your Heart' web-based cardiac rehabilitation programme n=259
Usual care n=259
Semi-structured telephone interviews (process evaluation) n=15
6 months' outcome measurement
6 months' outcome measurement
12 months' outcome measurement (n=207 if 80% retention)
12 months' outcome measurement (n=207 if 80% retention)
Semi-structured telephone interviews (process evaluation) n=15
Semi-structured telephone interviews (process evaluation) n=15

Appendix 1 ACTIVATE Trial Registration Data

Data category	Information
Registry and trial identification no.	ISRCTN10054455
Date of registration	16/09/2021
Funder	NIHR Evaluation, Trials and Studies Co-ordinating Centre (NETSCC); Grant code 131015
Sponsor	University of Liverpool
Contact for public enquiries	email: activate.trial@liverpool.ac.uk
Scientific title	Cardiac rehabilitation for people with chronic stable angina
Acronym	Angina Controlled Trial Investigating the Value of the 'Activate Your Heart' E-intervention (ACTIVATE)
Countries of recruitment	United Kingdom
Health condition	Angina pectoris
Intervention	Intervention comparator: Web-based cardiac rehabilitation ('Activate Your Heart')
	Control comparator: Usual care
Inclusion criteria	Adult patients (age >18 years)
	Chronic stable angina with at least 2 out of 3 of the following features:<br>i) Constricting central chest pain<br>ii) Precipitated by exertion or emotional stress<br>iii) Relieved by rest or glyceryl trinitrate spray
	Evidence of myocardial ischaemia from either a past medical history of acute coronary syndrome, myocardial infarction (MI) or revascularisation procedure at least 12 months in the past; or from imaging studies such as invasive coronary angiography, computerised tomography angiography or myocardial perfusion testing.
	Revascularisation procedures not planned and treated with medical treatments only, including people with previous MI, or previous revascularisation procedure who may have attended cardiac rehabilitation in the past.
Exclusion criteria	History of myocardial infarction (MI) within the last 12 months.
	History of revascularisation procedure within the last 12 months, or planned at the time of study recruitment.
	Participation in a cardiac rehabilitation programme within the last 12 months, or planned at time of study recruitment.

For peer review only

	Significant co-morbidities (as deemed by person confirming eligibility) that would limit participation in the exercise-based rehabilitation programme.
	Refractory angina on maximal medical therapy.
Study design	Interventional
	Randomised controlled trial
	Treatment, education or self-management, psychological and behavioural, complex intervention, cardiac rehabilitation
Recruitment start date	01/12/2021
Target sample size	518
Primary outcome	UK version of Seattle Angina Questionnaire (SAQ-UK), physical limitations domain
Secondary outcomes	SAQ-UK angina frequency and perception and treatment satisfaction domains, MRC dyspnoea scale, Hospital Anxiety and Depression Scale, General Self-Efficacy Scale. Physical activity measured with the ActivPAL accelerometer for 7 days, supplemented by the International Physical Activity Questionnaire. Cardiovascular fitness measured with the Incremental Shuttle Walk Test. Economic measures EuroQol EQ-5D-5L and Client Service Receipt Inventory.

For peer review only

<Trust/Site address 1>
<Trust/Site address 2>
<Trust/Site address 3>
<postcode>
Tel: <telephone number>

Participant Information Sheet for ACTIVATE

- You have been invited to take part in a research study. Before you decide, it is important that you understand why the research is being done and what it will involve.
- Please take time to read the following information carefully. **Part 1** tells you the purpose of the study and what will happen to you if you take part. **Part 2** gives you more detailed information about the conduct of the study.
- You can ask a member of your clinical team if there is anything that is not clear, or if you would like more information.
- If you wish you can discuss it with friends, relatives or get independent advice from your local Patient Advice and Liaison Service (PALS) or equivalent.
- **Taking part is voluntary**. You do not need to take part and you do not need to give a reason.
- ACTIVATE is a study to look at cardiac rehabilitation for people with long-term stable angina (chest pain that happens with exertion) to see if a cardiac rehabilitation programme is effective.

How to contact the study team:

If you have any questions about this study please talk to your research team:

- **Principal investigator name and Tel number**
- **Research Nurse name and Tel Number**

Contents:

Part 1

- Why are we doing the ACTIVATE study?
- Do I have to take part?
- What will happen to me if I take part?
- What will I have to do if I take part?
- How will I know which treatment I'm going to have?
- What are the benefits and risks of taking part?
- What are the alternatives for treatment?
- What happens if I change my mind?
- What if new information becomes available?
- What happens when the study stops?
- What if there is a problem?
- Will my taking part in the study be kept confidential?

Part 2

- Who is running the study?
- How will my information be collected and handled?
- What are my choices about how my information is used?
- Information sharing for other research
- Where can I find out more about how my information is used?
- What if there is a problem?

For peer review only - http://bmjopen.bmj.com/site/about/guidelines.xhtml

PART 1: Purpose of the study and what will happen if you take part

Why are we doing the ACTIVATE study?

The aim of the study is to improve the health of people with long-term angina which limits daily activities. We want to see whether a rehabilitation programme combined with usual heart care is better than usual heart care alone.

Angina is chest pain brought on by physical activity or stress and is caused by narrowed blood vessels restricting the flow of blood to the heart muscle. It can severely limit day to day activities. Cardiac rehabilitation programmes promote the physical and mental health of people with heart disease by increasing their understanding and improving their physical fitness levels. Cardiac rehabilitation is normally recommended after heart attacks or heart surgery, but not for people with stable angina who do not require an operation. Although cardiac rehabilitation might also benefit people with stable angina, more evidence is needed before it can be used in standard care.

Participants will be split into two groups randomly. Group A will receive usual care from your healthcare provider plus a cardiac rehabilitation programme called 'Activate Your Heart'. Group B will receive usual care from your healthcare provider. 'Activate Your Heart' is an interactive website for you to use at home. The programme was developed by a team of health care professionals, people with angina and software designers. The programme helps you to set goals, monitor progress and gives you feedback on how you are doing. It encourages you to gradually increase your activity level. A paper manual will be available for participants who are unable to use the online version or for those who feel they would prefer a paper version. We would also like participants to complete some questionnaires whilst taking part in the study and also wear a small device called an ActivPAL device which looks out how active you are.

We want to recruit 518 participants aged 18+ years with long-term stable angina. Each participant will be asked to take part in the study for 12 months.

Do I have to take part?

No, **taking part is voluntary**. It is up to you to decide whether or not you want to take part.

If you decide not to take part then you will still receive the usual treatment your healthcare provider offers.

If you decide to take part you can also choose to stop at any time without giving a reason. The decision you make on whether to take part or not will not affect the standard of care you receive now or in the future.

What will happen to me if I take part?

A member of the clinical team can talk to you in more detail about this study and you will be able to ask any questions that you have. If you have had all of your questions answered and are happy to take part then you will be asked to sign a consent form to confirm you want to take part.

Whichever group you are allocated, we will ask you to attend the visits shown in the flowchart on page 3.

What will I have to do if I take part?

Once you have signed the consent form, we will check and confirm that this study is suitable for you and you will be asked to follow the study plan (see study timeline on page 3). You will have to:

- Attend your GP surgery or hospital for follow-up visits over a 12-month period. It may be possible to do these over the telephone or video call.
- At each follow-up visit we will ask you to complete a set of questionnaires.
- If you are randomly allocated to receive the 'Activate Your Heart' intervention you will be invited to meet with someone who will explain how the system works. A member of the research team will contact you to arrange a suitable date to meet and give you instructions on where to meet.

For peer review only

For peer review only - http://bmjopen.bmj.com/site/about/guidelines.xhtml

Study Timeline

Week 0

Baseline Assessment (Both Groups) (90 minutes)

You will be asked to sign a consent form. Your healthcare provider will then do some routine assessments, ask you to complete some questionnaires and fit an ActivPAL

Week 1

7 days later and Randomisation (Both Groups) (30 minutes)

The ActivPAL will be removed and you will be asked to complete a questionnaire. You will be assigned to treatment group A or treatment group B by chance.

Week 2-3

Intervention Training (Group A only) (60 minutes)

You will be asked to attend training with our cardiac specialist on the 'Activate Your Heart' online programme.

Week 27

Follow-up Visit 1 (Both Groups) (60 minutes)

Your healthcare provider will do some investigations, ask you to complete some questionnaires and fit an ActivPAL

Week 28

7 days after Follow-up Visit 1 (Both Groups) (30 minutes)

The ActivPAL will be removed and you will be asked to complete a questionnaire.

Week 53

Follow-up Visit 2 (Both Groups) (60 minutes)

Your healthcare provider will do some investigations, ask you to complete some questionnaires and fit an ActivPAL

Week 54

7 days after Follow-up Visit 2 (Both Groups) (30 minutes)

The ActivPAL will be removed and you will be asked to complete a questionnaire.

Procedure	Description	Research Treatment or Standard of care
Activate your heart	This is a secure, interactive website designed for you to use at home. The programme is tailored to individual need and is in four stages, which can be completed in 8 weeks, but your access to the site and its features continue for 12 months. A paper version is also available for any participant who does not want to access the website.	**Research Treatment**
Questionnaires	We will ask you to complete a set of questionnaires about your health, and use of health services, when you start taking part, again after 6 months and then a final set of questionnaires at 12 months.	**Research Treatment**
Physical activity measured with the ActivPAL accelerometer	The ActivPAL device is a small, slim monitor worn on the thigh. We will ask you to wear it for 7 days when you first take part, for another 7 days after 6 months and then a final 7 days after 12 months. The device will look at how physically active you are. We will also ask you to keep a diary documenting working hours, sleep, reasons for not wearing it, and any other comments	**Research Treatment**

For peer review only - http://bmjopen.bmj.com/site/about/guidelines.xhtml

Procedure	Description	Research Treatment or Standard of care
Incremental Shuttle Walk Test	We will ask you to complete this test when you start taking part, again after 6 months and then finally at 12 months. For this walking test we will ask you to walk back and forth along a 10-metre track at a comfortable pace, every minute the pace will increase. The distance you can walk will give us an idea of your exercise capacity.	**Research Treatment**
Height, weight, blood pressure and Serum Cholesterol	These are standard tests that you will have done when you visit the Doctor, we would like to collect this information for our study.	**Standard of Care**

Optional telephone interviews

We will interview a selection of participants over the telephone to ask about your experience of being in the study. These will last up to 1 hour and will be recorded. We will offer a £30 shopping voucher for your time.

How will I know which treatment I get?

In the ACTIVATE study participants will be split into two treatment groups. Your healthcare provider will let you know what group you are in. We use a computer programme that puts participants into groups by chance:

- One group will receive standard of care treatment plus have access to the online 'Activate Your Heart' rehabilitation programme.
- The other group will receive standard of care treatment only.

Are there any risks of taking part?

Experience from using the 'Activate Your Heart' programme for cardiac rehabilitation has shown that it is very safe. There may be a small risk of injury or provoking an episode of angina when exercising, but to prevent this, the physical activity goals are carefully set. You can contact the cardiac rehabilitation therapists for advice and support or by joining an online scheduled weekly chat room.

What happens if I change my mind?

If at any point you decide to stop taking part in the study you will still receive your usual treatment from your healthcare provider. If you do decide to stop taking part we will ask you if you would like to: continue to complete follow up visits for the study or stop taking part with no more study visits.

What happens when the study stops?

When you finish taking part in the study your healthcare provider will discuss what treatment is best for you next.

The results of the study will be presented at conferences and published in medical journals. Confidentiality will be ensured at all times and you will not be identified in any publication.

Any information resulting directly or indirectly from this research, as well as any patents developed directly or indirectly as a result of this research may be used for commercial purposes. You have no right to this property or to any share of the profits that may be earned directly or indirectly as a result of this research. However, in signing this form for this research, you do not give up any rights that you would otherwise have as a participant in research.

What if there is a problem?

We don't expect there to be any problems the study has been carefully designed to ensure your safety is the main priority. Any complaint about the way you have been dealt with during the study or any possible harm you might suffer will be addressed. See Part 2 below.

Will my taking part be kept confidential?

For peer review only - http://bmjopen.bmj.com/site/about/guidelines.xhtml

Yes. All the confidential information about your participation in this study will be kept confidential. Detailed information on this is given in Part 2.

For peer review only

For peer review only - http://bmjopen.bmj.com/site/about/guidelines.xhtml

PART 2: Detailed Information about the conduct of the study

Who is running the study?

The University of Liverpool is the Sponsor of this study and is responsible for managing it. They have asked that the day to day running of the study is carried out by a team based at the Liverpool Clinical Trials Centre (LCTC, part of the University of Liverpool) and qualitative and health economic researchers (part of the University of Liverpool).

The study has been reviewed by the Health Research Authority and the North of Scotland Research Ethics Committee (2).

This study is funded by the National Institute for Health Research's Health Technology Assessment Programme (ref: NIHR: 131015).

Your healthcare provider will not receive any payment for including you in this study.

How will my information be collected and handled?

The University of Liverpool is the Data Controller for this study and will need to use information from you and your medical records for this research project.

This information will include your initials, NHS number and name. If you are in the rehabilitation group (Group A of the study), your contact details will be sent to the cardiac rehabilitation team, so that they can contact you to arrange an appointment to explain how to use the 'Activate Your Heart' programme. Your contact details will also be shared with qualitative researchers at The University of Liverpool so they can contact you for telephone/video-call interviews. People will use this information to do the research or to check your records to make sure that the research is being done properly.

Individuals from The University of Liverpool, the LCTC and regulatory organisations may look at your medical and research records to check the accuracy of the research study.

People who do not need to know who you are will not be able to see your name or contact details. Your data will have a code number instead.

Data will be entered into a secure database from your healthcare team and staff members at the LCTC will be able to see this data. The LCTC will share some data with qualitative researchers and health economics researchers. The researchers doing the interviews will contact 30 participants about 3 months after the start of the study and again after 12 months to ask questions about how they felt being part of the study. These interviews can take place on the telephone or video call.

Health economic researchers at The University of Liverpool want to use your health economic data (number of visits to hospital, medication taken etc.) to help calculate the overall costs of care. We will ask you to complete a questionnaire called Client Service Receipt Inventory (CSRI). This is a tool used to collect information on the whole range of services and supports study participants may use. We will share the information you provide in this questionnaire with health economic researchers at The University of Liverpool.

We will keep all information about you safe and secure.

Once we have finished the study, we will keep the data for 10 years, so we can check the results. We will write our reports in a way so that no-one can work out that you took part in the study.

You can find out more about how we use your information at www.activate-trial.org.uk

What are my choices about how my information is used?

You can stop being part of the study at any time, without giving a reason, but we will keep information about you that we already have.

For peer review only

For peer review only - http://bmjopen.bmj.com/site/about/guidelines.xhtml

If you choose to stop taking part in the study, we would like to continue collecting information about your health from central NHS records, your hospital and your GP. If you do not want this to happen, please tell your healthcare provider

If you lose capacity during the study we would still plan to use the information collected from you.

We need to manage your records in specific ways for the research to be reliable. This means that we will not be able to let you see or change the data we hold about you.

Information sharing for other research

When you agree to take part in a research study, the information about your health and care may be beneficial to researchers running other research studies in this organisation and in other organisations. These organisations may be universities, NHS organisations or companies involved in health and care research in this country or abroad. Your information will only be used by organisations and researchers to conduct research in accordance with the UK Policy Framework for Health and Social Care Research, or equivalent standards.

If you agree to take part in this study, you will have the option to take part in future research using your anonymous data saved from this study.

Where can I find out more about how my information is used?

You can find out more about how we use your information:

- at the study website: www.activate-trial.org.uk
- at www.hrs.nhs.uk/information-about-patients
- in the Health Research Authority leaflet available from www.hra.nhs.uk/patientdataandresearch
- by contacting the University of Liverpool Data Protection Officer on LegalServices@liverpool.ac.uk.
- by asking one of the research team

What if there is a problem?

If you have a concern about any aspect of this study, you should ask to speak with one of your research team who will do their best to answer your questions.

If you remain unhappy and wish to complain formally, you can do this by contacting local NHS Patient Advice and Liaison Service (PALS) or equivalent. Members of your local hospital team or GP should be able to provide this information to you.

Every care will be taken in the course of this study. However, in the unlikely event that you are harmed by taking part in this research project, compensation from the study Sponsor (The University of Liverpool) may be available, but you may have to pay your related legal costs. The place where you receive your treatment, has a duty of care to you whether or not you agree to participate in the study. The study Sponsor accepts no liability for negligence on the part of your GP practice or healthcare provider. However, if you are harmed, and this is due to someone's negligence at the GP practice or healthcare provider, then you may have grounds for a legal action for compensation against the NHS organisation where you are being treated but you may have to pay for your legal costs. The normal National Health Service complaints procedures should be available to you.

FUNDED BY NIHR | National Institute for Health Research

Thank you for taking the time to read and consider this information sheet. Should you decide to take part in the study, you will be given a copy of the information sheet and a signed consent form to keep.

For peer review only

For peer review only - http://bmjopen.bmj.com/site/about/guidelines.xhtml

ACTIVATE

<Trust/Site address 1>
<Trust/Site address 2>
<Trust/Site address 3>
<postcode>
Tel: <telephone number>

Participant Consent Form

FOR SITE USE ONLY:

Site Name:	
Participant Study Number	
Participant Initials:	Participant DOB: / /

To be completed by the participant:

Once you have read and understood each statement please enter your initials in each box.	Initial
1. I have read and understood the information sheet for this study. I have had the opportunity to ask questions and have had these answered satisfactorily.	
2. I understand that participation is voluntary and that I am free to withdraw from the study at any time, without giving a reason, and without my care or legal rights being affected. However, the study team may need to collect some limited information for safety reasons.	
3. I give permission for a copy of this fully completed consent form to be sent to the LCTC (where it will be kept in a secure location) to allow confirmation that my consent was given.	
4. I understand that relevant sections of my medical notes and any data collected during the study may be looked at by authorised individuals from the central study team and representatives of the Sponsor, regulatory authorities and my healthcare provider. I give permission for these individuals to have access to my records and data.	
5. I agree to my GP being informed of my participation in the study.	
6. I agree for my data collected on the Client Service Receipt Inventory (CSRI) form to be shared with researchers at The University of Liverpool for the purpose of health economic research.	
7. I understand that my data will be kept by the University of Liverpool and at my healthcare provider in a confidential manner for 10 years from the end of the study.	
8. I agree to take part in the above study.	
The statements below are optional (you can still take part in the study even if you do not wish to agree to these):	
9. I agree to allow information or results arising from this study to be used in future healthcare and/or medical research providing my confidentiality is maintained.	
10. I agree to being contacted by a study researcher to conduct a qualitative interview and for the interview to be recorded. (if you agree to this statement provide your details below):	
11. I agree for any of my feedback from the qualitative interviews to be used as quotes in future publications, presentations and on the study website and newsletters	

Telephone number:	
Email address	

Please continue to the next page.

For peer review only

For peer review only - http://bmjopen.bmj.com/site/about/guidelines.xhtml

ACTIVATE

<Trust/Site address 1>
<Trust/Site address 2>
<Trust/Site address 3>
<postcode>
Tel: <telephone number>

Participant Consent Form

FOR SITE USE ONLY:

Site Name:			
Participant Study Number			
Participant Initials:		Participant DOB:	/ /

To be completed by the participant:

Your full name (please print):			
Your signature:		Date:	

To be completed by the Researcher (after participant has completed the form):

Researcher full name (please print):			
Researcher signature:		Date:	

Please file the original wet-ink copy in the ACTIVATE Investigator Site File, and make three copies: one for the participant, one for the medical notes and one to be sent to the LCTC.

For peer review only - http://bmjopen.bmj.com/site/about/guidelines.xhtml

For peer review only

ACTIVATE SPIRIT Checklist

SPIRIT — STANDARD PROTOCOL ITEMS: RECOMMENDATIONS FOR INTERVENTIONAL TRIALS

SPIRIT 2013 Checklist: Recommended items to address in a clinical trial protocol and related documents*

Section/item	Item No	Description	Addressed on page number
**Administrative information**			
Title	1	Descriptive title identifying the study design, population, interventions, and, if applicable, trial acronym	1
Trial registration	2a	Trial identifier and registry name. If not yet registered, name of intended registry	1,4
	2b	All items from the World Health Organization Trial Registration Data Set	Appendix 1
Protocol version	3	Date and version identifier	20
Funding	4	Sources and types of financial, material, and other support	26
Roles and responsibilities	5a	Names, affiliations, and roles of protocol contributors	1,2,25
	5b	Name and contact information for the trial sponsor	26
	5c	Role of study sponsor and funders, if any, in study design; collection, management, analysis, and interpretation of data; writing of the report; and the decision to submit the report for publication, including whether they will have ultimate authority over any of these activities	26
	5d	Composition, roles, and responsibilities of the coordinating centre, steering committee, endpoint adjudication committee, data management team, and other individuals or groups overseeing the trial, if applicable (see Item 21a for data monitoring committee)	25

For peer review only

**Introduction**			
Background and rationale	6a	Description of research question and justification for undertaking the trial, including summary of relevant studies (published and unpublished) examining benefits and harms for each intervention	5,6
	6b	Explanation for choice of comparators	5,6
Objectives	7	Specific objectives or hypotheses	7
Trial design	8	Description of trial design including type of trial (eg, parallel group, crossover, factorial, single group), allocation ratio, and framework (eg, superiority, equivalence, noninferiority, exploratory)	7,8
**Methods: Participants, interventions, and outcomes**			
Study setting	9	Description of study settings (eg, community clinic, academic hospital) and list of countries where data will be collected. Reference to where list of study sites can be obtained	8
Eligibility criteria	10	Inclusion and exclusion criteria for participants. If applicable, eligibility criteria for study centres and individuals who will perform the interventions (eg, surgeons, psychotherapists)	8,9
Interventions	11a	Interventions for each group with sufficient detail to allow replication, including how and when they will be administered	9-11
	11b	Criteria for discontinuing or modifying allocated interventions for a given trial participant (eg, drug dose change in response to harms, participant request, or improving/worsening disease)	9-11
	11c	Strategies to improve adherence to intervention protocols, and any procedures for monitoring adherence (eg, drug tablet return, laboratory tests)	10-11
	11d	Relevant concomitant care and interventions that are permitted or prohibited during the trial	9-10

Outcomes	12	Primary, secondary, and other outcomes, including the specific measurement variable (eg, systolic blood pressure), analysis metric (eg, change from baseline, final value, time to event), method of aggregation (eg, median, proportion), and time point for each outcome. Explanation of the clinical relevance of chosen efficacy and harm outcomes is strongly recommended	12-13, 27-28
Participant timeline	13	Time schedule of enrolment, interventions (including any run-ins and washouts), assessments, and visits for participants. A schematic diagram is highly recommended (see Figure)	29-30
Sample size	14	Estimated number of participants needed to achieve study objectives and how it was determined, including clinical and statistical assumptions supporting any sample size calculations	13
Recruitment	15	Strategies for achieving adequate participant enrolment to reach target sample size	13

Methods: Assignment of interventions (for controlled trials)

Allocation:

Sequence generation	16a	Method of generating the allocation sequence (eg, computer-generated random numbers), and list of any factors for stratification. To reduce predictability of a random sequence, details of any planned restriction (eg, blocking) should be provided in a separate document that is unavailable to those who enrol participants or assign interventions	14
Allocation concealment mechanism	16b	Mechanism of implementing the allocation sequence (eg, central telephone; sequentially numbered, opaque, sealed envelopes), describing any steps to conceal the sequence until interventions are assigned	14
Implementation	16c	Who will generate the allocation sequence, who will enrol participants, and who will assign participants to interventions	14
Blinding (masking)	17a	Who will be blinded after assignment to interventions (eg, trial participants, care providers, outcome assessors, data analysts), and how	14

	17b	If blinded, circumstances under which unblinding is permissible, and procedure for revealing a participant's allocated intervention during the trial	14

Methods: Data collection, management, and analysis

Data collection methods	18a	Plans for assessment and collection of outcome, baseline, and other trial data, including any related processes to promote data quality (eg, duplicate measurements, training of assessors) and a description of study instruments (eg, questionnaires, laboratory tests) along with their reliability and validity, if known. Reference to where data collection forms can be found, if not in the protocol	12
	18b	Plans to promote participant retention and complete follow-up, including list of any outcome data to be collected for participants who discontinue or deviate from intervention protocols	12
Data management	19	Plans for data entry, coding, security, and storage, including any related processes to promote data quality (eg, double data entry; range checks for data values). Reference to where details of data management procedures can be found, if not in the protocol	12-13
Statistical methods	20a	Statistical methods for analysing primary and secondary outcomes. Reference to where other details of the statistical analysis plan can be found, if not in the protocol	15-16
	20b	Methods for any additional analyses (eg, subgroup and adjusted analyses)	16-20
	20c	Definition of analysis population relating to protocol non-adherence (eg, as randomised analysis), and any statistical methods to handle missing data (eg, multiple imputation)	16

Methods: Monitoring

Data monitoring	21a	Composition of data monitoring committee (DMC); summary of its role and reporting structure; statement of whether it is independent from the sponsor and competing interests; and reference to where further details about its charter can be found, if not in the protocol. Alternatively, an explanation of why a DMC is not needed	20,25
	21b	Description of any interim analyses and stopping guidelines, including who will have access to these interim results and make the final decision to terminate the trial	14-15
Harms	22	Plans for collecting, assessing, reporting, and managing solicited and spontaneously reported adverse events and other unintended effects of trial interventions or trial conduct	20-21
Auditing	23	Frequency and procedures for auditing trial conduct, if any, and whether the process will be independent from investigators and the sponsor	20

Ethics and dissemination

Research ethics approval	24	Plans for seeking research ethics committee/institutional review board (REC/IRB) approval	20-21
Protocol amendments	25	Plans for communicating important protocol modifications (eg, changes to eligibility criteria, outcomes, analyses) to relevant parties (eg, investigators, REC/IRBs, trial participants, trial registries, journals, regulators)	20-21
Consent or assent	26a	Who will obtain informed consent or assent from potential trial participants or authorised surrogates, and how (see Item 32)	8
	26b	Additional consent provisions for collection and use of participant data and biological specimens in ancillary studies, if applicable	N/A
Confidentiality	27	How personal information about potential and enrolled participants will be collected, shared, and maintained in order to protect confidentiality before, during, and after the trial	14,21,26 Appendix 2

review only

Declaration of interests	28	Financial and other competing interests for principal investigators for the overall trial and each study site	26
Access to data	29	Statement of who will have access to the final trial dataset, and disclosure of contractual agreements that limit such access for investigators	26
Ancillary and post-trial care	30	Provisions, if any, for ancillary and post-trial care, and for compensation to those who suffer harm from trial participation	26
Dissemination policy	31a	Plans for investigators and sponsor to communicate trial results to participants, healthcare professionals, the public, and other relevant groups (eg, via publication, reporting in results databases, or other data sharing arrangements), including any publication restrictions	21
	31b	Authorship eligibility guidelines and any intended use of professional writers	21
	31c	Plans, if any, for granting public access to the full protocol, participant-level dataset, and statistical code	26
**Appendices**			
Informed consent materials	32	Model consent form and other related documentation given to participants and authorised surrogates	Appendix 2
Biological specimens	33	Plans for collection, laboratory evaluation, and storage of biological specimens for genetic or molecular analysis in the current trial and for future use in ancillary studies, if applicable	N/A

*It is strongly recommended that this checklist be read in conjunction with the SPIRIT 2013 Explanation & Elaboration for important clarification on the items. Amendments to the protocol should be tracked and dated. The SPIRIT checklist is copyrighted by the SPIRIT Group under the Creative Commons "Attribution-NonCommercial-NoDerivs 3.0 Unported" license.

For peer review only
