# Supplementary material for: Effectiveness and cost-effectiveness of a web-based cardiac rehabilitation programme for people with chronic stable angina: protocol for the ACTIVATE (Angina Controlled Trial Investigating the Value of the ‘Activate your heart’ Therapeutic E-intervention) randomised controlled trial
Source: BMJ Open. 2024 Mar 25;14(3):e084509. doi: 10.1136/bmjopen-2024-084509 (PMC10966821; doi:10.1136/bmjopen-2024-084509)
Supplement: Reviewer comments [file bmjopen-2024-084509.reviewer_comments.pdf]

PEER REVIEW HISTORY

BMJ Open publishes all reviews undertaken for accepted manuscripts. Reviewers are asked to complete a checklist review form (**http://bmjopen.bmj.com/site/about/resources/checklist.pdf**) and are provided with free text boxes to elaborate on their assessment. These free text comments are reproduced below.

ARTICLE DETAILS

**TITLE (PROVISIONAL)**	Effectiveness and cost-effectiveness of a web-based cardiac rehabilitation programme for people with chronic stable angina: protocol for the ACTIVATE (Angina Controlled Trial Investigating the Value of the 'Activate your heart' Therapeutic E-intervention) randomised controlled trial
**AUTHORS**	Williams, Nefyn; Collins, Brendan; Comerford, Terence; Dodd, Susanna; Fisher, Michael; Hardwick, Ben; Hennessy, Sophie; Jolly, Kate; Jones, Ian; Lane, Deirdre; Lip, Gregory; Morgan, Erica; Ralph, Penelope; Thijssen, Dick; Singh, Sally

This article was not externally reviewed at BMJ Open. Protocols that have been independently assessed prior to submission to BMJ Open are usually fast-tracked to publication on the grounds that further substantial changes will not be possible. This independent assessment will usually be external, independent review for both a substantial grant award from a non-commercial or government funder and ethics approval.
